# Large and finite sample properties of a maximum-likelihood estimator for multiplicity of infection

**DOI:** 10.1371/journal.pone.0194148

**Published:** 2018-04-09

**Authors:** Kristan Alexander Schneider

**Affiliations:** Department CB, University of Applied Sciences Mittweida, Mittweida, Germany; Centers for Disease Control and Prevention, UNITED STATES

## Abstract

Reliable measures of transmission intensities can be incorporated into metrics for monitoring disease-control interventions. Genetic (molecular) measures like multiplicity of infection (MOI) have several advantages compared with traditional measures, e.g., *R*_0_. Here, we investigate the properties of a maximum-likelihood approach to estimate MOI and pathogen-lineage frequencies. By verifying regulatory conditions, we prove asymptotical unbiasedness, consistency and efficiency of the estimator. Finite sample properties concerning bias and variance are evaluated over a comprehensive parameter range by a systematic simulation study. Moreover, the estimator’s sensitivity to model violations is studied. The estimator performs well for realistic sample sizes and parameter ranges. In particular, the lineage-frequency estimates are almost unbiased independently of sample size. The MOI estimate’s bias vanishes with increasing sample size, but might be substantial if sample size is too small. The estimator’s variance matrix agrees well with the Cramér-Rao lower bound, even for small sample size. The numerical and analytical results of this study can be used for study design. This is exemplified by a malaria data set from Venezuela. It is shown how the results can be used to determine the necessary sample size to achieve certain performance goals. An implementation of the likelihood method and a simulation algorithm for study design, implemented as an R script, is available as S1 File alongside a documentation (S2 File) and example data (S3 File).

## Introduction

The decline of malaria incidence in sub-Saharan Africa and elsewhere shifted the focus of health authorities in many countries towards elimination. This renders the need to evaluate the effectiveness of control programs to reduce transmission more urgent. Indeed, codifying a set of metrics, suitable to easily and reliably measure the impact of new and existing control interventions on malaria transmission, is highly desirable. Of particular interest are metrics, capable to monitor changes in exposure and transmission intensity. A recent book chapter [1] extensively reviewed 11 metrics of malaria transmission with regard to precision, accuracy, methods of collection and cost efficiency. While the entomological inoculation rate (EIR) and the basic reproduction number *R*_0_ are still the gold standards to measure transmission in malaria, molecular metrics such as multiplicity of infection (MOI) and molecular force of infection (mFOI) emerged as most appropriate. The relevance of identifying suitable metrics to quantify transmission is not restricted to malaria, but applies equally to other infectious diseases. Notably, incidence of MOI or superparasitism per se is epidemiologically an important metric of exposure in infectious diseases [2–11].

MOI refers to the number of super-infections of a disease, typically visible by the occurrence of multiple genetic variants (‘lineages’) within an infection. This is indicative of transmission dynamics as it reflects the overlap of several genetic variants due to multiple infectious contacts. Hence, MOI relates to intra-host dynamics [12], i.e., the dynamics of interactions among different ‘lineages’ within infections, and its derived pathogenic and epidemiological consequences. The concept of MOI is closely related to that of complexity of infection [13, 14].

Intra-host dynamics have been the subject of several theoretical and experimental investigations exploring a broad spectrum of scenarios over the last decades [12, 15–18]. Importantly, intra-host dynamics affect the spread of parasite lineages with adaptive mutations conferring resistance to antimicrobial agents or that allow the evasion of immune and/or vaccine-mediated protection [19, 20]. Currently, this is of particular importance in malaria, as the spread of artemisinin tolerance/resistance is threatening to challenge control efforts [21–23]. In summary, following or measuring MOI is essential whenever epidemiological inferences are influenced by or theoretical models depend on intra-host dynamics.

Although for a given pathogen it is relatively easy to measure the number of distinctive pathogenic lineages in models and experimental settings (e.g., [24]), it is not possible to define a universal framework of MOI that is appropriate for the vast spectrum of genetic architectures observed in pathogen organisms. For instance, viruses like HIV accumulate mutations at a rate that allows for the use of phylogenetic base methods [25]. On the contrary, eukaryotic parasites such as *Plasmodium*, *Trypanosoma*, *Toxoplasma*, and *Schistosoma* [26, 27] and bacteria such as *Mycobacterium* [28] evolve at a rate at which it is possible to determine a stable number of genetically distinct lineages during the course of an infection given a set of genetic markers. Even when restricted to such pathogens, due to difficulties naturally arising from confounding factors in ecological and epidemiological investigations [2, 3, 5, 28, 29], the concept of MOI is enervated. Estimating MOI and frequency spectra was dominated by ad-hoc methods, which are intuitive but typically introduce a bias, which cannot be quantified due to the lack of a statistical framework. E.g., in the context of malaria a patient’s MOI is estimated as the maximum number of distinct alleles detected in a blood sample from one (e.g. [30, 31]), two (e.g. [32]) or several marker loci (e.g. [33]). Being a lower bound for the number of parasite haplotypes in a sample, this underestimates MOI (particularly if only a few markers are considered), with bias depending on the haplotype-frequency spectrum. On the other hand, MOI might by overestimated as a result of data artifacts (sequencing errors, wrong STR calls)—particularly with a large number of genetic markers considered. MOI was also estimated as the cumulative number of lineages (alleles) identified across samples divided by the number of disease-positive samples. This corresponds to the sum of the empirical prevalences of the respective lineages (e.g. [34]). Some authors additionally reported MOI as the average number of observed alleles separately for different marker loci (e.g. [35]). Alternatively, MOI was estimated as the average number of alleles at several marker loci (e.g. [36]). Also the number of polymorphic SNPs was used as an indirect measure for MOI by [37].

Other methods, try to estimate MOI using a statistical framework that does not simultaneously provide frequency estimates but rather suggest the use of ad-hoc frequency estimates (e.g. [13, 38]). Often haplotype (lineage) frequencies are estimated from single-infections (those in which only one haplotype is found; e.g. [37]). This method is justified if MOI is low with most infections being single infections. Increasing MOI raises uncertainty in each sample because super-infections with the same haplotype cannot be distinguished from single infections. Hence, sample size is decreased as multiple infections become more likely, which are excluded from the estimate. This approach is particularly unfortunate because samples containing most information about MOI (multiple infections) are excluded. Similar problems arise if single infections are employed for frequency estimation along those, in which only two distinct haplotypes occur (e.g. [39]). Another ad-hoc approach to estimate allele frequencies, which accounts for multiple infections by giving all alleles found at a marker in a multiple infection the same weight, was employed by [40]. Although sample size is not artificially reduced, this method does not properly consider the interaction of frequency spectra and MOI. A further alternative is to employ only the predominant lineage (haplotype) in infections for frequency estimation (cf. [34]).

Hence, a formal statistical framework that allows the estimation of the actual number of lineages and other approximations to MOI that facilitates and/or considers confounding factors is indispensable to avoid ad-hoc methods.

In the context of malaria (and related diseases) such a framework was introduced by [41] and further developed by [42] and [43]. More precisely, a maximum-likelihood framework was developed to estimate MOI and the frequency of pathogen ‘lineages’ from molecular data obtained from a collection of blood samples of disease-positive patients.

Notably, several alternative methods, based on essentially the same statistical framework, have been proposed. However, all have some limitations or use heuristic approximations. Common to all is that they focus on specific applications and the mathematical properties have not been studied in detail. The computer program MalHaploFreq by [44] uses a maximum-likelihood approach to estimate frequencies of haplotypes consisting of up to 3 SNPs, without providing an estimate of MOI. The latter is also not provided by the Gibbs-sampling method used in [45] to estimate haplotype frequencies, again designed for SNP data, but allowing for more than 3 SNPs. The same is true for the Metropolis-Hastings algorithm used in [46], which is not restricted to SNP data. The EM-algorithm adapted in [47] allows for many SNPs but frequency estimates are based on an approximation considering only the most frequent haplotypes. Heuristic estimates of MOI are required in the Metropolis Hastings algorithm of [48] for frequency estimates not restricted to SNPs. The EM and MCMC algorithms in [49], which are based on several approximations to make them numerically tractable, again focus on SNP data but provide MOI estimates. The program COIL [13] uses a likelihood approach for MOI estimation from SNP data. The algorithm however requires ad-hoc estimates of marginal frequencies, SNPs to be uncorrelated, and assumes a maximum possible MOI of 5. An Improvement, THE REAL McCOIL [14] adapts a Metropolis-Hastings algorithm to estimate MOI and minor-allele frequencies at uncorrelated SNPs in two different ways. The program estMOI [50] requires deep sequencing data. The formal statistical framework in [42] uses an EM algorithm for SNP data.

Here, we will further investigate the framework of [41, 43] and the concept of MOI with regard to the criteria pointed out by [1]. First, we will prove a number of regulatory conditions, which imply asymptotic unbiasedness, strong consistency and efficiency of the maximum-likelihood estimate. In addition to these asymptotic properties we numerically investigate the estimator’s finite sample properties by conducting a systematic simulation study to quantify the estimator’s bias (accuracy) and variance (precision). Importantly, we also investigate the estimator’s robustness to model violations.

First, we summarize the methods and derive analytic results. Then, we will describe the simulation study and summarize its outcome. As an illustration we will take a closer look at a malaria data set from Venezuela, previously published in [51].

An implementation of the likelihood method in R, which can be readily applied to molecular data, is available as supporting information, alongside a simulation algorithm for study design to determine the necessary sample size to achieve given accuracy and precision goals of the estimation (S1, S2 and S3 Files).

## Materials and methods

Here, we briefly summarize the maximum-likelihood method to estimate the average MOI, proposed by [41] and [43].

### Model background

Assume *n* different ‘lineages’ *A*_1_, …, *A*_*n*_ of a pathogen, e.g., *n* alleles at a marker locus (or haplotypes in a non-recombining region), which circulate in a given population and are found in *N* blood samples of infected individuals—or more generally *N* clinical specimens. A blood sample can contain multiple lineages reflecting super-infections. Regarding the lineages, it is assumed that they are characterized by markers (SNPs or STRs), whose frequencies do not change too rapidly in the population. Because the frequency spectrum of markers linked to genes under selection might change rapidly or might be very skewed, we have neutral markers in mind. The *n* lineages considered are those that contribute to infection, not new variants that are generated by mutation inside hosts (and ‘fail’ to participate in transmission). The frequencies of the *n* lineages are denoted by ***p*** = (*p*_1_, …, *p*_*n*_). The frequency vector ***p*** is an element of the (*n* − 1)-dimensional simplex $S_{n}:\{(x_{1},\ldots ,x_{n})|\sum_{i=1}^{n}x_{1}=1\;\text{and}\;x_{i}>0\;\text{for}\;\text{all}\;i\}$.

Infective events are assumed to be rare and independent, i.e., already infected persons are not more or less likely to get infected (super-infected) than uninfected persons. With these assumptions the number of infections per individual is Poisson distributed, or more precisely conditionally Poisson (or positively Poisson) distributed since only infected individuals are considered (Eq S30). The conditional Poisson distribution is characterized by a single parameter λ. Jointly we denote the parameters by $\theta =\left(\lambda ,p\right)\in \Theta ≔R^{+}\times \mathrm{int}S_{n}$, where int $S_{n}$ denotes the interior of the (*n* − 1)-dimensional simplex. In other words the parameters satisfy λ > 0, $\sum_{i=1}^{n}p_{i}=1$ and 0 < *p*_*k*_ < 1 for *k* = 1, …, *n*.

It is further assumed that at each infective event one lineage is drawn randomly from the pathogen population (according to the lineages’ frequencies) to infect the individual. Hence, if an individual is infected exactly *m* times (which is a conditional Poisson random number), *m* lineages are drawn according to a multinomial distribution with parameters *m* and *p*_1_, …, *p*_*n*_. This yields a vector (*m*_1_, …, *m*_*n*_), where *m*_*k*_ is the number of times the individual was infected with lineage *A*_*k*_. Clearly, *m*_1_ + … + *m*_*n*_ = *m*, because the individual is infected exactly *m* times. Looking at a blood sample, only the absence and presence of lineages is observed, but it is impossible to reconstruct the values *m*_*k*_ or even *m*. Even if only one lineage is found, it is unclear how many times the individual was infected with this lineage. Hence, looking at a blood sample only a 0–1 vector ***i*** = (*i*_1_, …, *i*_*n*_) is observed, indicative of the present lineages, i.e., *i*_*k*_ = 1 if *m*_*k*_ > 0 and *i*_*k*_ = 0 if *m*_*k*_ = 0. The probability of observing an infection with configuration ***i*** is

$$\begin{array}{c} Q_{i}=\frac{1}{e^{\lambda }-1}\prod_{j=1}^{n}{\left(e^{\lambda p_{j}}-1\right)}^{i_{j}} \end{array}$$ (1)

according to [43]. Notably, the probabilistic model defined by (1) is identifiable.

**Remark 1** *The probability functions* (1) *defined by any two values of* ***θ*** ∈ Θ *are distinct*.

The proof is presented in Appendix A in S4 File. It is important to point out that *p*_*k*_ is the relative frequency of lineage *A*_*k*_ in not its prevalence. The former refers to the relative abundance of the lineage in the parasite population, the latter to the probability that the lineage is present in an infection (within the population of disease-positive individuals).

**Remark 2** *Lineage A*_*k*_’s *prevalence*, *i*.*e*., *the probability of observing A*_*k*_ *in a disease-positive blood sample*, *is*

$$q_{\left\{k\right\}}≔q_{k}=\sum_{\underset{i_{k}=1}{i\in {\left\{0,1\right\}}^{n}\setminus \left\{0\right\}:}}Q_{i}=\frac{e^{\lambda }\left(e^{\lambda p_{k}}-1\right)}{e^{\lambda p_{k}}\left(e^{\lambda }-1\right)}.$$ (2)

A proof is provided in Appendix B in S4 File.

A data set obtained from *N* blood samples consists of *N* 0-1-vectors, indicating which lineages are detected. We denote the *j*th blood sample by $x_{j}=\left(x_{1j},\ldots ,x_{nj}\right)$. Collectively, the data is denoted by ***X***. Further, *N*_*k*_ is the number of samples in which lineage *A*_*k*_ is detected, i.e., $N_{k}=\sum_{j=1}^{N}x_{kj}$. Under the outlined model, the log-likelihood of observing data ***X*** is given by

$$\begin{array}{c} L=L\left(\lambda ,p\right)=L\left(\lambda ,p|X\right)=-N\;\text{log}\;\left(e^{\lambda }-1\right)+\sum_{k=1}^{n}N_{k}\;\text{log}\;\left(e^{\lambda p_{k}}-1\right) \end{array}$$ (3)

(cf. [43]). Obviously, (*N*, *N*_1_, …, *N*_*k*_) form a sufficient statistic for the data ***X***. The value *N*_*k*_/*N* is the observed prevalence of lineage *k*.

### Maximum-likelihood estimate

The maximum-likelihood estimate (MLE) for (λ, ***p***) exists and is uniquely defined except in two irregular situations. In the first, only one lineage is found in each blood sample, i.e., $\sum_{k=1}^{n}N_{k}=N$, i.e., there is no indication of super-infections. In the second, at least one lineage is found in every blood sample, i.e., *N*_*k*_ = *N* for at least one *k*. We can state (cf. [43]):

**Remark 3** *Except in irregular situations*, *the MLE* $\hat{\theta }=\left(\hat{\lambda },\hat{p}\right)$ *is given by*

$$\begin{array}{c} {\hat{p}}_{k}=-\frac{1}{\hat{\lambda }}\;\text{log}\;(1-\frac{N_{k}}{N}\left(1-e^{-\hat{\lambda }}\right))\;, \end{array}$$ (4a)

*where* $\hat{\lambda }$ *is found by iterating*

$$\begin{array}{c} \lambda_{t+1}=\lambda_{t}-\frac{\lambda_{t}+\sum_{k=1}^{n}\;\text{log}\;(1-\frac{N_{k}}{N}\left(1-e^{-\lambda_{t}}\right))}{1-\sum_{k=1}^{n}\frac{N_{k}}{Ne^{\lambda_{t}}-N_{k}\left(e^{\lambda_{t}}-1\right)}}\;, \end{array}$$ (4b)

*which converges monotonically at quadratic rate from any initial value* $\lambda_{1}\geq \hat{\lambda }$. *Hence*, *it is guaranteed to find the MLE as long as the initial value* λ_1_ *is chosen to be sufficiently large*.

*If* $N=\sum_{k=1}^{n}N_{k}$ *and N*_*k*_ ≠ *N for all k*, $\hat{\lambda }=0$ *and* ${\hat{p}}_{k}=\frac{N_{k}}{N}$. *If N*_*k*_ = *N for at least one k*, *the MLE does not exist* (“$\hat{\lambda }=\infty$”).

An implementation of the algorithm above is provided as an R script (S1 File) alongside a documentation (S2 File).

Because the MLE does not exist if *N*_*k*_ = *N* or $\sum_{k=1}^{n}N_{k}=N$, for study design it is important to minimize the probability of obtaining irregular data. Moreover, one might prefer conditioning the likelihood on a regular data set for analytical investigations. In Appendix B in S4 File the probability of observing irregular data is calculated to be

$$\begin{array}{c} q≔\frac{1}{{\left(1-e^{-\lambda }\right)}^{N}}\left(1-\prod_{j=1}^{n}\left(1-{\left(1-e^{-\lambda p_{j}}\right)}^{N}\right)\right)+{\left(\sum_{j=1}^{n}Q_{e_{j}}\right)}^{N}-\sum_{j=1}^{n}Q_{e_{j}}^{N} \end{array}$$ (5)

(***e***_*j*_ denote the standard base vectors). Clearly, this probability vanishes as *N* → ∞. However, if *N* and λ are small and the lineage frequencies are very skewed, observing irregular data is likely.

The problem of irregular data can be avoided by imposing restrictions on the parameter space, except if only one lineage is observed in the data, i.e., $\sum_{k=1}^{n}N_{k}=N$ and *N*_*j*_ = *N* for some *j*. The MLE can be adapted as follows.

**Result 1** *Assume the true parameter* ***θ***_0_ *lies within the interior of the compact set* $\hat{\Theta }=\left[\lambda_{\min},\lambda_{\max}\right]\times S_{n}$. *The maximum-likelihood estimate is given by Remark 3 if N*_*k*_ ≠ *N for all k*, $\sum_{k=1}^{n}N_{k}>N$ *and* $\hat{\lambda }\in \left[\lambda_{\min},\lambda_{\max}\right]$. *If* $\hat{\lambda }<\lambda_{\min}$ *or* $\sum_{k=1}^{n}N_{k}=N$ (*but N*_*j*_ ≠ *N for all j*), *the MLE is given by* $\hat{\theta }=\left(\lambda_{\min},\hat{p}\right)$, *where*

$$\begin{array}{c} {\hat{p}}_{k}=-\frac{1}{\lambda_{\min}}\;\text{log}\;(1-\frac{N_{k}\lambda_{\min}}{\hat{\beta }})\;, \end{array}$$ (6a)

*where* $\hat{\beta }$ *is found by iterating*

$$\begin{array}{c} \beta_{t+1}=\beta_{t}-\beta_{t}\frac{\lambda_{\min}+\sum_{k=1}^{n}\;\text{log}\;(1-\frac{N_{k}\lambda_{\min}}{\beta_{t}})}{\sum_{k=1}^{n}\frac{N_{k}\lambda_{\min}}{\beta_{t}-N_{k}\lambda_{\min}}}\;, \end{array}$$ (6b)

*which is guaranteed to converge from any initial value β*_1_ *satisfying* $\max_{k=1,\ldots ,n}N_{k}\lambda_{\min}<\beta_{1}<\hat{\beta }$. *If* $\hat{\lambda }>\lambda_{\max}$ *or N*_*k*_ = *N for any k* (*but* $\sum_{k=1}^{n}N_{k}>N$), *the MLE is given by* $\hat{\theta }=\left(\lambda_{\max},\hat{p}\right)$, *where* $\hat{\beta }$ *is given by* (6) *with* λ_max_ *replaced by* λ_min_.

*If only one lineage is present in the data*, *i*.*e*., $\sum_{k=1}^{n}N_{k}=N$ *and N*_*j*_ = *N for some j*, *the MLE is not unique*, *more precisely any estimate* (λ, ***e***_*j*_) *is equally likely*.

A proof is found in Appendix B in S4 File.

## Results

### Large-sample properties

Usually MLEs have attractive limiting properties under relatively weak conditions. To prove these here it is more convenient to regard the admissible parameter space as a subset of $R^{n}$. This is achieved by eliminating one of the redundant frequencies. We set $p_{n}=1-\sum_{k=1}^{n-1}p_{k}$, ***ϑ*** = (λ, *p*_1_, …, *p*_*n*−1_) and $\tilde{\Theta }=\left\{\left(\lambda ,p_{1},\ldots ,p_{n-1}\right)\;|\;\lambda_{\min}\leq \lambda \leq \lambda_{\max},\;0<p_{k}\;\forall \;k\;\text{and}\;\sum_{k=1}^{n-1}p_{k}<1\right\}$. Let $\hat{\vartheta }$ denote the corresponding MLE, which, of course, equals $\hat{\theta }$ with the last component dropped. In the new parameter space the Fisher information matrix is derived as follows.

**Result 2** *The Fisher information matrix*, $I_{N}\left(\vartheta \right)≔-E\frac{\partial^{2}L}{\partial \vartheta^{2}}$, *is given by*

$$\begin{array}{c} I_{1,1}=\frac{-Ne^{\lambda }}{{\left(e^{\lambda }-1\right)}^{2}}+\frac{Ne^{\lambda }}{e^{\lambda }-1}\sum_{k=1}^{n}\frac{p_{k}^{2}}{e^{\lambda p_{k}}-1}\;, \end{array}$$ (7a)

$$\begin{array}{c} I_{1,k+1}=I_{k+1,1}=\frac{N\lambda }{1-e^{-\lambda }}(\frac{p_{k}}{e^{\lambda p_{k}}-1}-\frac{p_{n}}{e^{\lambda p_{n}}-1})\;for\;k=1,\ldots ,n-1\;, \end{array}$$ (7b)

$$\begin{array}{c} I_{k+1,k+1}=\frac{N\lambda^{2}}{1-e^{-\lambda }}(\frac{1}{e^{\lambda p_{k}}-1}+\frac{1}{e^{\lambda p_{n}}-1})\;for\;k=1,\ldots ,n-1\;, \end{array}$$ (7c)

$$\begin{array}{c} I_{k+1,j+1}=\frac{N\lambda^{2}}{1-e^{-\lambda }}\frac{1}{e^{\lambda p_{n}}-1}\;for\;k,\;j=1,\ldots ,n-1,\;k\neq j\;. \end{array}$$ (7d)

The information matrix is derived in Appendix C in S4 File and two of its important properties are proved (cf. Theorem 1 and Theorem 2 in S4 File), namely:

**Remark 4** *The Fisher information matrix is positive definite and satisfies*

$$\begin{array}{c} I_{N}=-E\frac{\partial^{2}L}{\partial \vartheta^{2}}=E\left({\left(\frac{\partial L}{\partial \vartheta }\right)}^{T}\cdot \left(\frac{\partial L}{\partial \vartheta }\right)\right). \end{array}$$ (8)

Indeed for the MLE we can state the following result.

**Result 3** *The MLE specified in Result 1 is*

*(i) strongly consistent*, *i*.*e*. $\hat{\theta }\overset{a.s.}{\to }\theta_{0}$,
*(ii) asymptotically unbiased*, *i*.*e*., $E\hat{\theta }\to \theta_{0}$,
*(iii) efficient*, *i*.*e*., $\left(\text{Var}\;\hat{\theta }\right)V\to I_{n\times n}$,
*(iv) asymptotically normally distributed*, *i*.*e*., $\hat{\theta }∼N\left(\theta_{0},V\right)$,

*where the asymptotic covariance matrix V* = (*v*_*ij*_) *is the Cramér-Rao lower bound given by*

$$\begin{array}{c} v_{11}=\frac{{\left(e^{\lambda }-1\right)}^{2}}{Ne^{\lambda }}\;\;\frac{C}{e^{\lambda }-1-C}\;, \end{array}$$ (9a)

$$\begin{array}{c} v_{1j}=v_{j1}=\frac{{\left(e^{\lambda }-1\right)}^{2}}{\lambda Ne^{\lambda }}\;\;\frac{e^{\lambda p_{j}}-1-p_{j}C}{e^{\lambda }-1-C}\;, \end{array}$$ (9b)

$$\begin{array}{c} v_{ii}=\frac{{\left(e^{\lambda }-1\right)}^{2}}{\lambda^{2}Ne^{\lambda }}(\frac{e^{\lambda p_{i}}-1}{e^{\lambda }-1}+\frac{p_{i}^{2}C-2p_{i}\left(e^{\lambda p_{i}}-1\right)+\frac{{\left(e^{\lambda p_{i}}-1\right)}^{2}}{e^{\lambda }-1}}{e^{\lambda }-1-C})\;, \end{array}$$ (9c)

$$\begin{array}{c} v_{ij}=\frac{{\left(e^{\lambda }-1\right)}^{2}}{\lambda^{2}Ne^{\lambda }}\;\;\frac{p_{i}p_{j}C-p_{i}\left(e^{\lambda p_{j}}-1\right)-p_{j}\left(e^{\lambda p_{i}}-1\right)+\frac{\left(e^{\lambda p_{i}}-1\right)\left(e^{\lambda p_{j}}-1\right)}{e^{\lambda }-1}}{e^{\lambda }-1-C}\;, \end{array}$$ (9d)

*for i*, *j* = 2, …*n* + 1, *i* ≠ *j and*

$$\begin{array}{c} C=\sum_{k=1}^{n}\left(e^{\lambda p_{k}}-1\right) \end{array}$$ (9e)

**Proof**. First, the true parameter ***θ***_0_ lies in the interior of Θ. Hence, a compact subset $\hat{\Theta }⊊\Theta$ exists, such that $\theta_{0}\in \mathrm{int}\hat{\Theta }$. By eliminating the redundant variable *p*_*n*_ this is equivalent to $\vartheta_{0}\in \mathrm{int}\;\Theta^{1}\subseteq R^{n}$, where $\Theta^{1}\subseteq \tilde{\Theta }$ is compact. Second, the model is identifiable according to Remark 1. Third, the first three derivatives of the log-likelihood function with respect to the parameters exist and $\frac{1}{N}\frac{\partial^{3}L}{\partial \vartheta^{3}}$ is uniformly bounded on Θ^1^ according to Remark 1. Fourth, the Fisher information satisfies (8) and is positive definite. Hence, the regulatory conditions given in [52] (Chapter 4, p.118) are satisfied. These imply strong consistency, asymptotical unbiasedness and efficiency of $\hat{\vartheta }$ and hence $\hat{\theta }$. The Cramér-Rao lower bound is derived in Appendix D in S4 File.

The mean MOI is given by $\psi =\frac{\lambda }{1-e^{-\lambda }}$ rather than by the Poisson parameterλ and might be preferable. Since MLEs are transformation respecting, $\hat{\psi }=\frac{\hat{\lambda }}{1-e^{-\hat{\lambda }}}$ holds. Also the Cramér-Rao bound needs some adjustment (see Appendix E in S4 File).

**Remark 5** *The Cramér-Rao bound* $\tilde{V}$ *of the MLE* $\left(\hat{\psi },{\hat{p}}_{1},\ldots ,{\hat{p}}_{n}\right)$ *is given by*

$$\begin{array}{c} {\tilde{v}}_{1,1}=\frac{e^{\lambda }{\left(e^{\lambda }-\lambda -1\right)}^{2}}{N{\left(e^{\lambda }-1\right)}^{2}}\;\;\frac{C}{e^{\lambda }-1-C}\;, \end{array}$$ (10a)

$$\begin{array}{c} {\tilde{v}}_{1,j}=\frac{e^{\lambda }-\lambda -1}{\lambda N}\;\;\frac{e^{\lambda p_{j}}-1-p_{j}C}{e^{\lambda }-1-C}\;, \end{array}$$ (10b)

$$\begin{array}{c} {\tilde{v}}_{ij}=v_{ij}\;, \end{array}$$ (10c)

*where i*, *j* = 2, …*n* + 1 *and v*_*ij*_ *an C are given by* (9c), (9d) *and* (9e).

### Finite sample properties of the MOI estimate

The desirable properties of the MLE hold only in the large-sample limit given that the parametric model (1) is correct. In practice, the MLE’s quality depends on (i) the model’s fit, (ii) the true parameters, and (iii) sample size. To investigate these dependencies, we conduct a systematic numerical study. All numerical investigations were performed in R version 3.1.0 [53]. A detailed description is found in Appendix F in S4 File. The main R code for the simulations is provided as supporting information (S1 File), adapted for users to run their own simulations. All detailed results are provided as S5 File.

#### Mean and median bias

**Fig 1:**
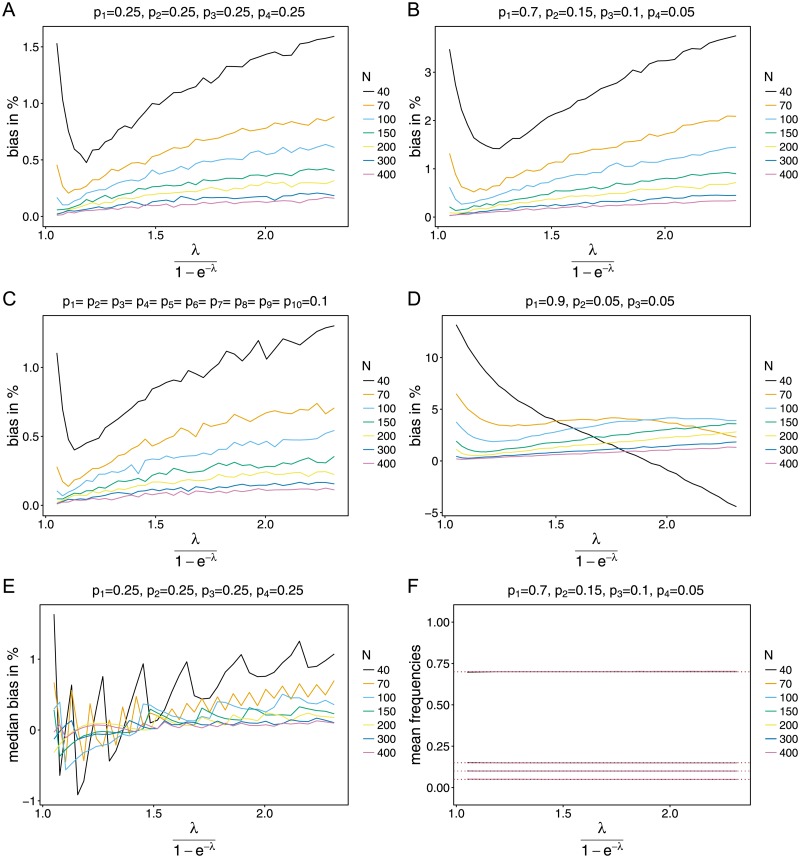
Bias for the conditional Poisson model. (A)-(D) Shown is the bias of the MLE $\hat{\psi }$ in percent as a function of the true parameter *ψ* based on simulated data created by the conditional Poisson model. Each panel assumes different *n* and lineage frequency distributions ***p*** shown at the top of each panel. Each line is for a different sample size *N*. (E) Shown is the median bias rather than the mean bias. (F) Average estimates for the lineage frequencies *p*_1_ = 0.7, *p*_2_ = 0.15, *p*_3_ = 0.1 and *p*_4_ = 0.05 (marked by the red-dotted horizonal lines) for different sample sizes. Since the lineage-frequency estimates are almost unbiased—independently of the sample size—the lines corresponding to different *N* almost coincide. Hence, only the top lines (purple lines for *N* = 200) at *p*_1_ = 0.7, *p*_2_ = 0.15, *p*_3_ = 0.1 and *p*_4_ = 0.05 and the dotted red lines on top are visible.

The MLE for MOI, $\hat{\psi }=\frac{\hat{\lambda }}{1-e^{-\hat{\lambda }}}$ is—as typically for MLEs—biased, however as shown analytically bias vanishes as sample size *N* increases (Fig 1A–1D and S5 File). The maximum bias is about 4%. There is a tendency of overestimating the true parameter in a non-linear fashion. As *ψ* increases, bias first decreases until *ψ* ≈ 1.2, and then starts to increase almost linearly (Fig 1 and S5 File). Overestimation occurs on average because *ψ* is bounded from below by 1, whereas it has no upper bound. Estimates for *ψ* will be occasionally much too large while they cannot be much too small. This is particularly likely for very small and large *ψ*. For these reasons it seems better to use the median bias as a proxy for the estimate’s accuracy (see below).

Bias vanishes quickly as *N* increases. A considerable reduction occurs when sample size is increased from 40 to 50. For *N* = 100 bias is already low, but there is still a remarkable gain by increasing sample size to 150–still a realistic sample size. Bias typically stays below ≈ 0.5% then and almost vanishes for small values of *ψ*. Increasing sample size to 200, yields an improvement mainly for values beyond *ψ* ≈ 1.7, which however is a rather extreme parameter range in practice. Bias almost vanishes for *N* ≥ 300, at least for moderate values of *ψ* (Fig 1 and S5 File).

While the overall pattern described above is valid regardless of the lineage-frequency distribution, higher skewness leads to increased bias (compare Fig 1A with 1B)–particularly, if one lineage is dominating whereas others have frequency of ≈ 5%. The effect is not too strong if *N* ≥ 100, in which case bias stays below 5%. This is not surprising, because a highly frequent lineage super-infects with high probability. In a sample this cannot be detected as a super-infection, and thus leads to a small underestimation of *ψ*. However, sometimes low-frequency lineages are over-represented (they will occur together with high-frequency lineages), leading occasional to huge over-estimates. Consequently, rare outliers create bias. Since the median is robust against outliers, skewed frequency distributions do not affect the median bias.

Bias decreases with an increasing number of lineages (Fig 1 and S5 File) while the qualitative pattern described above remains unchanged. The reason is that for larger *n*, super-infections are more accurately represented. Namely, for given *n* super-infections with *n* + 1 or more lineages cannot be identified. Although this leads more likely to underestimates, occasionally huge over-estimates occur. Over-represented super-infections are interpreted as large MOI due to the underlying Poisson model. For these reasons and those mentioned above, for very skewed frequency spectra (Fig 1 and S5 File), bias increases drastically, especially if sample size is small. For small *ψ* bias increases up to 15%. For extremely large *ψ* bias becomes negative, because samples indicative of super-infections with several lineages become rare, as the same lineages are infecting multiple times.

**Fig 2:**
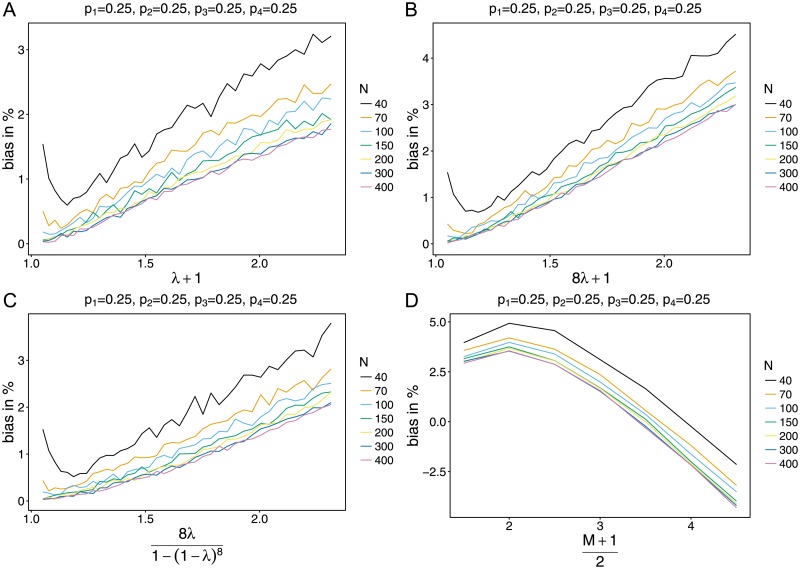
Dependence on true model. Shown is the bias as a function of the true parameter *ψ*, for *n* = 4 and a balanced lineage-frequency distribution. The underlying true models are the shifted Poisson, shifted binomial, conditional binomial and uniform models (cf. Appendix F in S4 File) in panels (A), (B), (C), (D), respectively.

Slight model violations do not change the overall pattern of bias much (cf. Fig 2A–2C). Bias tends to increase faster with *ψ* but typically stays less than 2% for *N* ≥ 150 and realistic values of *ψ*. For the most extreme parameters bias stays below 15%. It tends to be a little higher for the shifted binomial model (cf. Appendix F in S4 File) than for the other two alternatives, which is not surprising since this model constitutes the largest model violation among them. Assuming the uniform model—a radical model violation—changes the overall pattern (cf. Fig 2 and S5 File). As *ψ* increases, bias first increases (from typically 5%–10%) slightly and then becomes strongly negative—however for an extreme parameter range. Bias improves if sample size increases, for more lineages (larger *n*), and more balanced lineage-frequency distributions.

Concluding, bias is moderate if sample size is sufficiently large *N* ≥ 150, which seems to be a reasonable compromise between feasibility and accuracy.

Bias, measured by the median, is usually by an order of magnitude smaller (compare Fig 1A with 1E), for the reasons mentioned above. In fact it is almost absent, particularly if sample size is sufficiently large (*N* ≥ 100) and *ψ* ≤ 1.7. Although the maximum median bias (for small sample size, extremely skewed lineage frequencies, and extremely high MOI) is up to 15%, it is typically much lower. Hence, median bias can be regarded as being absent if sample size is ≥ 100.

It should be pointed out that bias is reported conditional on regular data here. The probability of irregular data satisfying $\sum_{k=1}^{n}N_{k}=N$ increases as λ and *N* decrease. Such data result in estimates of $\hat{\lambda }=0$ or $\hat{\lambda }=\lambda_{\min}$, which are close to the true value. Therefore, if bias is not conditioned on $\sum_{k=1}^{n}N_{k}>N$, it almost vanishes for small λ, but depends on the choice of λ_min_. As the asymptotic results on the estimator assume regular data, we decided to present bias only conditional on regular data.

#### Variance

**Fig 3:**
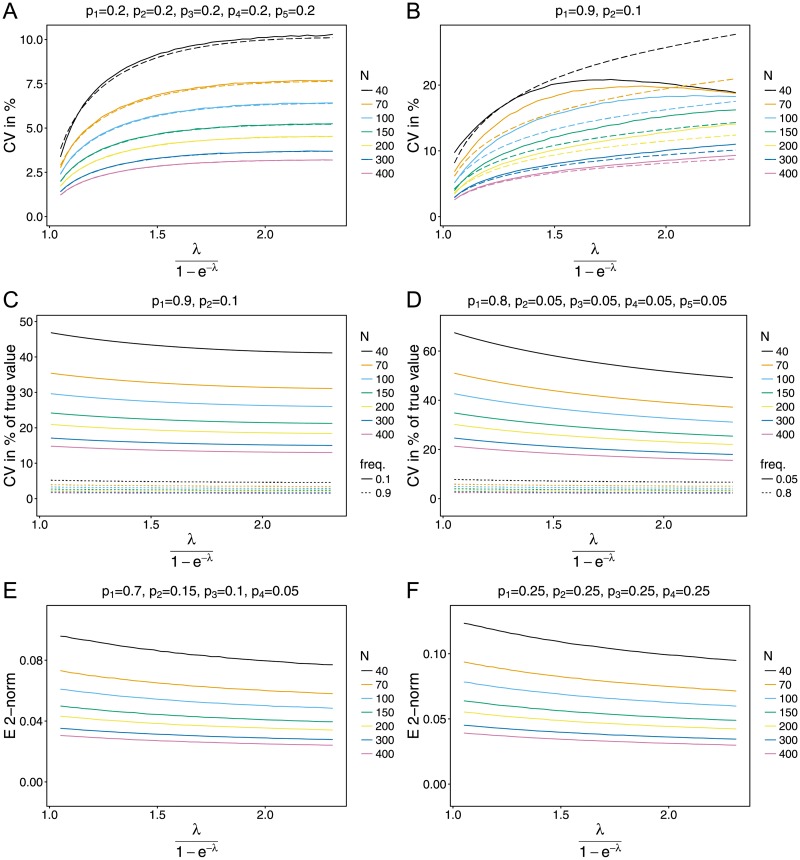
Measures of variation. (A)-(B) CV of $\hat{\psi }$ in % (i.e. ×100) as a function of *ψ* for the conditional Poisson model. The dashed line is the respective prediction based on the Cramér-Rao lower bound. Almost identical pictures are obtained for the other models (conditional Poisson, shifted Poisson, conditional binomial and shifted binomial). Panels are for different ***p*** (with different lineage numbers *n*). (C)-(D) CV for lineage frequencies. Shown is the theoretical prediction which is almost indistinguishable from the curves obtained by simulation, for all models. Panels are for different ***p*** (with different lineage numbers *n*). (E)-(F) Average Euclidian distance of the MLE $\hat{p}$ and the true parameter ***p***. Shown are the curves obtained from the conditional Poisson model. Panels are for different ***p*** (with different lineage numbers *n*).

Variation of the estimator of *ψ* is best measured relative to the mean, i.e., by the coefficient of variation (CV). For most parameter combinations, the CV increases with *ψ* in a Holing-Type-II fashion (see Fig 3 in S5 File). This is particularly true for balanced lineage-frequency distributions. The CV decreases with increasing sample size and typically stays below 20% for *N* = 40 and on the order of 5% for *N* = 400. The CV is smaller for a larger number of lineages *n*. For skewed lineage-frequency distributions, the CV might be largest for intermediate *ψ* and small sample size *N* ≤ 100 (cf. Fig 3D). Not surprisingly, these results are robust against model violations; in fact the CVs cannot be distinguished visually. The observed pattern can be explained from the results concerning bias. If the true *ψ* is small, bias is created mainly by rare large overestimates, while any underestimate will be close to the true value. This hardly affects the (empirical) variance of $\hat{\psi }$, which is small by nature if the true value of *ψ* is small. Thus, relative to the average estimate, which is an overestimate, variance is small. For larger true values of *ψ*, variance increases naturally. Moreover, an underestimate $\hat{\psi }$ will no longer be very close to the true value, which further contributes to an increased variance. Finally, relative to the average $\hat{\psi }$, which now properly reflects the true value, the CV is large than for small *ψ*. This effect does not result in a linear increase of the CV, because the average $\hat{\psi }$ increases disproportionately compared with its variance.

To evaluate the estimator’s efficiency the CV can be compared to its theoretical prediction, i.e., to the square root of the Cramèr-Rao lower bound (given by Eq 10a) divided by the true value of *ψ*. For at least intermediate sample size *N* ≥ 150, the CV is very close to its theoretical prediction unless the lineage-frequencies are extremely skewed. In the latter case the CV is typically much smaller than its prediction for large *ψ* (see Fig 3D).

Notably, variance is rather large compared with bias. An approximate 95% confidence interval for the estimator relative to its true value is obtained as the relative bias ±2×CV.

Concluding, the variation is typically almost identical to its theoretical minimum for *n* ≥ 3, *N* ≥ 100 and balanced lineage-frequencies. For *N* ≥ 150, realistic *ψ* and not too skewed ***p***, the CV is on the order of 5%.

### Finite sample properties of the frequency estimates

#### Bias

The picture for the frequency estimates is much simpler than for the MOI parameter (Fig 1F). They are (almost) unbiased, independently of the sample size (*N*), the number of lineages (*n*), skewness of the lineage-frequency distribution, and true underlying model (S5 File). Whereas the MLE’s performance of the MOI parameter depends on all these (at least to some extend), the frequency estimates are robust against these factors (S5 File).

#### Variance

There are several variance measures for frequency estimates $\hat{p}$. The simplest approach is to consider the variances of each lineage frequency separately. The advantage is that the variances are comparable with the Cramér-Rao bounds, which perfectly matches the empirical variance. However, as the number of lineages *n* increases, these measures become tedious, especially since one is typically interested in functions or summaries of the distribution, rather than in the frequencies separately.

Like the bias, the variances of the estimates do not depend on the true underlying model. Not surprisingly, the variance is typically small, particularly if the true frequencies are small. Hence, it is more appropriate to consider the coefficient of variation, which implies only quantitative but no qualitative changes.

Since the variance is robust to model violations, so is the CV. Fig 3 shows the theoretical prediction of the CV, i.e., $\frac{\sqrt{{\tilde{I}}_{k,k}^{-1}}}{p_{k}}$. The observed CV, i.e., square root of sample variance divided by sample mean of ${\hat{p}}_{k}$, almost perfectly matches the theoretical prediction and it cannot be distinguished between the different underlying models. However, the CV depends on the number of lineages (*n*), sample size (*N*), MOI (*ψ*) and skewness of the allele frequency distribution. The CV decreases with increasing sample size and *ψ*. This is rather intuitive, since the data contains increasingly more information. For large *ψ*, more super-infections occur, which contain more information about the lineage frequencies, hence not only large *N* but also $\sum_{k=1}^{n}N_{k}$ imply more information per lineage. The CV tends to increase with an increasing number of lineage (*n*) and decreasing lineage-frequency. Again this is not surprising, because rare lineages hardly occur in a data set and this lack of information results in an increased variance. The CV is around 15% for balanced lineage frequencies. However, for *n* = 10 and lineage frequencies of 0.01, the CV increase to a substantial amount of 150% (S5 File). Nevertheless, for the major lineage frequencies, the CV typically stays under 10% if *N* ≥ 150.

Considering the CV is conservative, because one is typically interested in summary statistics of the lineage frequencies such as heterozygosity (cf. [54]). Such statistics will be insensitive against various factors, since small lineage frequencies—which have a large relative, but a very small absolute error—hardly affect them. Variance measured by the average Euclidian distance to the true parameter gives similar results (Fig 3E and 3F). The average distance is robust against model violations and decreases with increasing *ψ* and sample size *N* for the reasons mentioned above. The average Euclidian distance also increases with an increasing number of lineages. However, unlike the CV, it decreases for more skewed lineage-frequency distributions (Fig 3E and 3F), because the absolute error of minor lineages hardly affects the distance measures.

### Data application

The malaria dataset from [51] is examined more closely. From 97 malaria positive blood samples, 56 microsatellite loci were assayed, where 12 of the 56 marker loci can be considered selectively neutral. Hence, these are appropriate for the ML methods (cf. [43]).

Four of the 12 selected marker loci violated the assumption $\sum_{k=1}^{n}N_{k}>N$ (irregular data). In other words a third of the neutral markers did not provide sufficient information, a fact which can be avoided in the future by using the results provided here. Namely, it is possible to calculate a proxy of the necessary sample size to avoid irregular data sets.

First, note that typically some STR markers fail to amplify in a number of blood samples. It is not obvious how to proceed with the resulting missing data. Namely, a sample with a missing value cannot be considered disease free, since only disease-positive samples are analyzed. Moreover, it is not clear whether missing values are completely random or if there is a non-ignorable dependence between missing values, markers and repeat length, e.g., certain markers will amplify better than others or the probability for at least one lineage to amplify is higher if several variants super-infect. Hence, missing data depends on many confounding factors, which are difficult or impossible to determine. A pragmatic approach to handle missing data is to treat each marker as a data set and ignore, for each marker, those sample with a missing value. Consequently, this results in a different sample size for each marker.

Having decided on how to proceed with missing values, the MLEs for the lineage frequencies can be used as an estimate of the true frequencies. This can also be done in case that $\sum_{k=1}^{n}N_{k}=N$ (cf. Result 3). The median of the MLEs, λ_*me*_ = 0.7213, (derived from those 8 marker loci to which the method is applicable) can be used as an estimate proxy for the true parameter. This is justified because the median should be almost unbiased. Based on $\hat{p}$ and λ_*me*_ the probabilities of obtaining an irregular data set (Eq 5) is listed in Table 1. Clearly, these probabilities are substantially large. Based on these estimates, the probability that at least four of the 12 markers yield irregular data is ≈ 28% (if all 12 markers are independent, which is a valid assumption for neutral markers). To reduce the probability of obtaining irregular data in every marker (independently) below 5%, sample size needs to be increased to *N* = 300. The probability that four or more markers yield irregular data is then ≈ 3.4 × 10^−6^. However, the probability to obtain at least one irregular data set is still ≈ 13%. Increasing sample size to *N* = 400 reduced this probability to less than 5%.

**Table 1:** Results for Venezuela data. Shown are sample size *N*, MLEs for $\hat{p}$ and $\hat{\lambda }$, an estimate for the square root of the Cramér-Rao lower bound for *ψ*, the probabilities of obtaining an irregular data set derived from (5) using the estimates $\hat{p}$ and λ_*me*_ for the actual sample size *N* (*q*), sample size *N* = 300 (*q*_300_) and *N* = 400 (*q*_400_), respectively. The estimate λ_*me*_ is the median of the 8 estimates for which $\hat{\lambda }>0$ (regular data). In pairwise comparisons these eight estimates were not found to be significantly different at a 5% level based on pairwise likelihood-ratio tests provided in [43].

locus	*N*	$\hat{p}$	$\hat{\lambda }$	$\sqrt{\text{CR}}$	*q*	*q*_300_	*q*_400_	bias
U5	7	(0.78, 0.09, 0.08, 0.01, 0.01, 0.01, 0.01, 0.01)	0.055	0.065	0.265	0.015	0.005	≈ 0.5%
K6	95	(0.785, 0.205, 0.01)	0.06	0.065	0.305	0.025	0.005	≈ 0.5%
L1	96	(0.53, 0.47)	0.085	0.055	0.175	0.005	0.	≈ 0.8%
c4	74	(0.63, 0.36, 0.015)	0.055	0.065	0.275	0.005	0.	≈ 0.5%
b3	88	(0.6, 0.26, 0.13, 0.01)	0.12	0.055	0.165	0	0.	≈ 1%
fr13	97	(0.72, 0.27, 0.01)	0.1	0.06	0.235	0.01	0.005	≈ 0.5%
ps6	97	(0.64, 0.36)	0.09	0.055	0.195	0.005	0.	≈ 0.8%
ps7	81	(0.6, 0.28, 0.11, 0.01)	0.045	0.055	0.195	0	0.	≈ 0.5%
J3	96	(0.625, 0.28, 0.085, 0.01)	0	0.055	0.16	0.005	0.	≈ 0.5%
J6	96	(0.845, 0.085, 0.075)	0	0.075	0.38	0.05	0.02	≈ 1.4%
U6	97	(0.65, 0.28, 0.07)	0	0.055	0.175	0.005	0.	≈ 0.5%
L4	91	(0.69, 0.12, 0.09, 0.075, 0.01, 0.01)	0	0.055	0.195	0.005	0.	≈ 0.8%

Table 1 also shows the square root of the Cramér-Rao lower bound. Notice, that this is the lower bound for *ψ*. Hence, it should be compared with the respective median *ψ*_*me*_ = 1.037. The corresponding coefficient of variation is about 5%. Finally, the bias of the MLEs of *ψ* can be approximately obtained by looking up the bias for similar parameters from the simulation study. The maximum estimate of the bias (1.4%) is obtained for marker *J*6. Mostly, bias is as low as 0.5%.

Summarizing, a sample size of *N* = 97 is sufficient to obtain accurate and precise estimates, as justified by the proxies of bias obtained from the simulation study and the Cramér-Rao lower bounds based on Table 1. The quality of the estimates is also reflected by the fact that all 8 ‘regular markers’ yield estimates which are not statistically significant in pairwise comparisons (cf. [43]). However, this sample size is insufficient to guarantee with high probability that the data for each marker is regular. To guarantee regular data for 12 markers with at least 95% probability a sample size of 400 is necessary. Such calculations can already be performed during study design, if there are some vague ideas about the true parameters. Of note, it might be difficult to collect *N* = 400 samples in a low transmission area like Venezuela. In this case, a sample size of *N* = 100 should be sufficient, but many molecular markers should be assayed.

## Discussion

A central goal of infectious-disease control programs is the reduction of the circulating pathogen’s population size. Understanding the genetic changes associated with diminishing population size may provide valuable metrics to monitor success of control interventions. The reason is that population-genetic parameters reflect transmission intensities more accurately than incidence data—at least they will complement incidence data. Two quantities are starting to be more recognized in this context in epidemiology [1, 55], molecular force of infection and multiplicity of infection (MOI). The potential gain of incorporating such genetic/molecular information to infer transmission compared to traditional measures, e.g., entomological inoculation rate (EIR) or basic reproduction numbers (*R*_0_), which are notoriously difficult to estimate, is starting to be realized [1, 55].

The aim of this article was to obtain a better understanding for the approach of [41] and [43] to estimate MOI and “lineage” frequency spectra in infections. A detailed description on how MOI relates to quantities such as molecular force of infection, EIR or *R*_0_ can be found in [1]. While MOI might be built into a metric for monitoring transmission, accurate estimates of lineage-frequency spectra are desirable for monitoring the evolutionary dynamics of an endemic disease and for calculating frequency-based statistics. The method explored here is applies to diseases, for which infections are rare and independent events, and the course of the disease is relatively short. More precisely, de novo mutations should accumulate rarely within an infection. Hence, the method will not be applicable to pathogens like HIV, but to diseases like malaria.

To further investigate the properties of the maximum-likelihood approach of [43], we conducted a comprehensive numerical robustness study, which was complemented by additional analytical findings. (An implementation of the method and the simulation algorithm that can readily visualize the outcomes is available as S1 File). The study was designed under the criteria outlined in [1]. Particularly, we wanted to quantify the quality of the MOI estimate and the estimates for lineage frequencies in terms of bias and variance—with regard to different parameters and model violations. The method’s advantage is its simplicity. Namely, to calculate the estimates from *N* blood samples or clinical specimens, in which *n* lineages are detected, it suffices to determine the numbers *N*_*k*_ of blood samples in which lineage *k* is found. The MLE can be calculated from the numbers *N*_1_, …, *N*_*n*_ and *N*.

We proved usual attractive properties (asymptotic unbiasedness, strong consistency, efficiency) for the MLE, which also has good finite sample properties. The MLE yields reliable results if sample size is at least moderately large (*N* ≥ 150). The method performs better if the lineage-frequency spectrum is not too skewed, MOI (*ψ*) is small, and more lineages are circulating (larger *n*) for the following reasons. If the same lineage is super-infecting multiple times, information is lost, as it is indistinguishable whether this lineage was infecting just once or more than once. This loss of information occurs with higher probability if the lineage-frequency spectrum is skewed, MOI is high, or the number of different lineages is small. In other words, not just sample size *N*, but also $\sum_{k=1}^{n}N_{k}$ is a measure of how much information is available. With balanced frequency spectra $\sum_{k=1}^{n}N_{k}$ will tend to be larger than for unbalanced ones, resulting in more reliable estimates.

The MLE is only asymptotically unbiased. This is true particularly for the MLE for MOI (as measured by the average bias), whereas the estimates for the lineage frequencies are (almost) unbiased—even under model violations. The MOI parameter is biased, because it has a lower but no upper bound and unlikely data lead to disproportionately large estimates. However, the MOI estimate is almost unbiased, if bias is measured by the median. This is not true with model violations, although the median bias is still smaller than the mean bias. Therefore, the method appears nevertheless applicable if sample size is sufficiently large (*N* ≤ 150).

In general, the variance of the estimates is small—however it is large compared with bias. Especially, the Cramér-Rao bounds are good predictors for the estimator’s variance, again regardless of model violations. Particularly, if there is some prior idea of the true parameters’ ranges, these bounds are extremely helpful for effective study design (with respect to sample size and properties of the genetic/molecular markers) to achieve a certain precision goal. Since the estimator’s variance is close to the Cramér-Rao bounds, there is not much room for improvement for the estimator. However, the estimator might be generalized to include information from several genetic markers at the same time. A simple ad-hoc approach to reduce variance would be to average several MOI estimates from different (uncorrelated) markers. This and more sophisticated methods are subject to future work.

The fact that the lineage frequencies’ estimates are unbiased and the small variances seem promising to use these estimates for genetic statistics. Note, however, that the coefficient of variation might be rather large for minor lineages, which is not surprising since they are likely either under- or over-represented. Hence, it might be problematic to use such estimates in genetic statistics, which rely on minor allele frequencies. Statistics such as heterozygosity or statistics that use ranked frequency spectra should nevertheless be rather robust.

Unfortunately, the method will not always be applicable. It is required that the data contains at least one detectable super-infection $\sum_{k=1}^{n}N_{k}>N$ and that no lineage is found in all samples 0 < *N*_*k*_ < *N* for all *k*. Violations of these requirements cannot be easily resolved by just adding pseudo-counts, because estimates would be very sensitive to the exact details of the adjustment. Nevertheless, we calculated the probability to obtain a sample to which the method is inapplicable. These results can again be used for study design to guarantee with high probability that the method will be applicable.

This issue was demonstrated with a malaria data set from Venezuela [51]. Particularly, the MLE for MOI was obtained from several neutral microsatellite markers as well as their respective allele frequency spectra. The method was not applicable to some markers since super-infections were not observed (irregular data sets). This is not surprising although sample size is moderate (*N* = 97) considering that the samples were taken in an area of low transmission. A sample size of at least *N* = 300 would have been necessary to guarantee regular data with 95% probability for each marker separately. To guarantee with 95% probability that no marker yields irregular data, sample size needs to be at least *N* = 400. Such considerations should be taken into account during study design.

A drawback of the maximum-likelihood-approach studied here is its dependence on the Poisson assumption. This assumption can in principal be relaxed by assuming e.g. a negative binomial distribution for the number of infectious events (infective contacts that cause an infection), which was in fact done by [41]. A negative binomial distribution arises if the number of infections is distributed heterogeneously across the population. More precisely, if the population consists of infinitely many patches, within which the number of infectious events are Poisson distributed and the Poisson parameters across patches follow a Γ-distribution. This is justified for malaria by the queuing model of [56]. However, even if the number of infectious events is negatively-binomially and not Poisson distributed, as suggested by empirical evidence (e.g. [57]), taking into account the fact that not every infectious event is infective, the number of infective events might be accurately approximated by a Poisson distribution. So far, an analytical treatment as presented here and in [43] has not be established under the assumption of a negative binomial distribution and is subject to future research.

Finally, it should be mentioned that an alternative to the frequentist method further investigated here is provided by the Bayesian framework of [13, 14]. Agreement of both approaches would underline the quality of both approaches. However, the comparison lies beyond the scope of the present study.

## Supporting information

S1 File R script. Programming code and implementation of likelihood method as R script.
(R) Click here for additional data file.

S2 File Documentation. Documentation of the R script (S1 File).
(PDF)

S3 File Example data. ZIP archive containing example data sets in various formats.
(ZIP)

S4 File Appendix. Appendix A-F.
(PDF)

S5 File Additional figures. Additional figures showing detailed results.
(PDF)
