# Supplementary material for: Large and finite sample properties of a maximum-likelihood estimator for multiplicity of infection
Source: PLoS One. 2018 Apr 9;13(4):e0194148. doi: 10.1371/journal.pone.0194148 (PMC5890990; doi:10.1371/journal.pone.0194148)
Supplement: S2 File — Documentation of the R script (S1 File). (PDF) [file pone.0194148.s002.pdf]

Supporting Information
Description of R Script “MOI-MLE.R” (S1 R Script)

Kristan Alexander Schneider

1 Calculating the MLE

Three simple functions need to be run in order to calculate the MLE from a molecular-data-set. First data needs to be imported using the function “DatImp”, then the lineage counts need to be derived using the function “Nk” and finally the MLE is calculated using the function “MLE”.

1.1 Importing data using “DatImp”

The function “DatImp(path)” imports a file containing molecular data. Here “path” is the location where the file is stored. Data needs to be stored in a standardized way (see section 1.2) as either ".xlsx", “.xls”, “.csv” or “.txt”.

Code example: The following code imports the file “STR.xlsx” (available in S3 Example Data), which is stored in “C:\Documents\Molecular Data”, and reads it into a data array. Only the first 10 lines of the output are shown.

```
> DatImp("C:/Documents/Molecular Data/STR.xlsx")
         ID  marker
1    MCP001     132
2      <NA>     144
3    MCP002     132
4      <NA>     126
5    MCP003     180
6    MCP003     144
7    MCP004      NA
8    MCP005     144
9    MCP006      NA
10     <NA>     132
```

To read the data into an array named “dat” use the code:

```
> dat <- DatImp("C:/Documents/Molecular Data/STR.xlsx")
```

1.2 Data format

Molecular data needs to be stored either as “.xlsx”-, “.xls”-, “.csv”- or “.txt”-file in a specific format. Examples can be found in the archive file S3 Example Data. We describe the format for “.xlsx”-files. A data set consists of two columns, the first contains the sample IDs, the second molecular information from samples. Each sample is stored in a $2 \times k$ block. In the first column at least the first row must contain the sample ID. The lineages found in a sample are stored in the second column in consecutive rows in any arbitrary order. Below are four alternative schematic descriptions of a sample in which lineages 1, 2 and 4 were found. Note that missing values can occur, that the same lineage might be entered multiple times for a sample (but it is counted only once) and that the sample ID must only occur in the first row. Missing values must be left empty. Examples:

ID1	lineage 2
	lineage 4
	lineage 1

ID1	lineage 1
ID1	lineage 2
	lineage 4

ID1	lineage 1
	lineage 2
	lineage 4
	lineage 4

ID1	lineage 1
	lineage 2
	lineage 4
ID1	lineage 4

Sample IDs and lineages are entered as numbers or strings. See the additional file STR.xlsx for an example of microsatellite data and SNP.xlsx for SNP data provided in S3 Example Data. The first row is reserved for column labels. It can be left empty, but this row must not be omitted. The table below shows the first 10 rows of the example data set STR.xlsx. It contains 6 samples.

ID	marker
MCP001	132
	144
MCP002	132
	126
MCP003	180
MCP003	144
MCP004	
MCP005	144
MCP006	
	132

The first sample (MCP003) contains two lineages, "132" and "144", corresponding to repeat lengths. Sample MCP003 contains the lineages "144" and "180". Note that the sample ID is entered in both rows for sample MCP003 but only in the first row for sample MCP001. Sample MCP004 has missing values. Such a sample will be excluded from the estimations. It will not contribute to sample size. Sample MCP006 contains only lineage "132", however the data is entered in an awkward way.

If the data is stored as a ".txt"-file, columns are separated by a tab stop. If it is entered as a ".csv" file columns, are separated by a semicolon (examples are found in S3 Example Data).

1.3 Frequency counts using the function "Nk"

The function "Nk(dat)" takes a $2 \times s$ array containing the molecular data and yields sample size (excluding samples with missing data) and lineage-frequency counts as a list object. The first element of the list is the sample size N, the second a matrix with the frequency counts $N_1, \dots, N_n$. The column names of the matrix are the respective lineages.

Code example: This code takes the array "dat" and calculates sample size and lineage frequency counts. The data corresponds to the sample data set "STR.xlsx" (see S3 Example Data):

```
> Nk(dat)
[[1]]
[1] 97

[[2]]
     126 132 144 150 180
[1,]  22  25  49  32  18
```

The data contains $N = 97$ samples that contain molecular information (two samples, namely MCP004, MCP008 have missing values). Five different STR repeats (lineages) are found in the data, namely, 126, 132, 144, 150, 180. Their respective counts are $N_1 = 22$, $N_2 = 25$, $N_3 = 49$, $N_4 = 32$ and $N_5 = 18$.

1.4 The function "MLE"

The function "MLE(Nk,N,la)" calculates the maximum likelihood estimate (MLE) $(\hat{\lambda}, \hat{p}_1, \ldots, \hat{p}_n)$ for data $(N_1, \ldots, N_n)$ derived from N blood samples. The output is a list containing five elements: (1) the value of the log-likelihood function evaluated at the MLE, (2) the estimate $\hat{\lambda}$, (3) the estimated frequencies $(\hat{p}_1, \ldots, \hat{p}_n)$, (4) sample size N, and (5) the lineage counts $(N_1, \ldots, N_n)$.

Code example: This code calculates the MLE for data from $N = 97$ samples with frequency counts $N_1 = 22$, $N_2 = 25$, $N_3 = 49$, $N_4 = 32$ and $N_5 = 18$:

```
> MLE(c(22,25,49,32,18),97)
[[1]]
[1] -259.8194

[[2]]
[1] 1.218736

[[3]]
[1] 0.1428264 0.1643813 0.3608585 0.2169937 0.1149401

[[4]]
[1] 97

[[5]]
     126 132 144 150 180
[1,]  22  25  49  32  18
```

Here, $L(\hat{\lambda}, \hat{\boldsymbol{p}}) = -259.8194$, $\hat{\lambda} = 1.218736$, $\hat{p}_1 = 0.1428264$, $\hat{p}_2 = 0.1643813$, $\hat{p}_3 = 0.3608585$, $\hat{p}_4 = 0.2169937$, and $\hat{p}_5 = 0.1149401$. Moreover, $N = 97$ and $(N_1, N_2, N_3, N_4, N_5) = (22, 25, 49, 32, 18)$.

Code example: This code shows how to calculate the MLE for the date stored in "STR.xlsx" in the

"/Users/Darth Vader/Documents":

```
> dat <- DatImp("/Users/Darth Vader/Documents/STR.xlsx")
> MLE(Nk(dat)[[2]],Nk(dat)[[1]])
[[1]]
[1] -259.8194

[[2]]
[1] 1.218736

[[3]]
[1] 0.1428264 0.1643813 0.3608585 0.2169937 0.1149401

[[4]]
[1] 97

[[5]]
     126 132 144 150 180
[1,]  22  25  49  32  18
```

The output is just the same as in the previous example.

2 Simulations to ascertain precision and accuracy goals

2.1 The function "cpoiss"

The function "cpoiss(λ, n)" generates n random numbers from a conditional poisson distribution with parameter λ.

Code example: This code generates 10 random numbers from a conditional Poisson variable with parameter $\lambda = 1.5$:

```
> cpoiss(1.5,10)
[1] 3 1 3 3 1 1 1 1 1 1
```

2.2 The function "mnom"

The function "mnom(M,$\boldsymbol{p}$)" generates a random vector $(m_1, \ldots, m_n)$ from a multinomial distribution with parameters M and $\boldsymbol{p} = (p_1, \ldots, p_n)$. The argument M is either a positive integer or a vector of positive integers, $M = (M_1, \ldots, M_k)$, in which case the output is a $k \times n$ matrix, where the ith row $(m_{i1}, \ldots, m_{in})$ follows a multinomial distribution with parameters M_i and $\boldsymbol{p}$.

Code example: This code generates a multinomial random vector with parameters $M = 8$ and $\boldsymbol{p} = (\frac{1}{4}, \frac{1}{4}, \frac{1}{4}, \frac{1}{4})$:

```
> mnom(8,c(0.25,0.25,0.25,0.25))
     [,1] [,2] [,3] [,4]
[1,]    1    6    1    0
```

Code example: This code generates a multinomial random vector with parameters $M = (8, 5, 6)$ and $\boldsymbol{p} = (\frac{1}{4}, \frac{1}{4}, \frac{1}{4}, \frac{1}{4})$:

```
> mnom(c(8,5,6),c(0.25,0.25,0.25,0.25))
     [,1] [,2] [,3] [,4]
[1,]    1    4    0    3
[2,]    0    2    1    2
[3,]    4    1    0    1
```

2.3 The function "run1"

The function "run1(λ,$\boldsymbol{p}$, N, K,path)" generates randomly K data sets consisting of N samples. Each data set is generated by first drawing MOI m randomly from a conditional Poisson distribution with parameter λ, then picking m lineages from a multinomial distribution with parameters m and $\boldsymbol{p}$, and deleting duplicates.

The function then calculates the MLE $(\hat{\lambda}, \hat{p}_1, \ldots, \hat{p}_n)$ for each of the K data sets and stores summary statistics of the K MLEs in a text file, specified by "path". The arguments of the function are the true parameters λ and $\boldsymbol{p}$, sample size N, the number K of simulation runs, i.e., the number of regular

data sets of sample size N created from a conditional Poisson distribution, and a path to an output text file.

The output text file stores the following information. The true parameters λ, $p_1, \ldots, p_n$, N, K, the number of data sets (regular and irregular) that were created (irregular data sets are discarded in all calculations), minimum, 1st quartile, median, mean, 3rd quartile, maximum and variance of the K MLE estimates $\frac{\hat{\lambda}}{1-e^{-\hat{\lambda}}}$ followed by the same statistics for each estimate $\hat{p}_1, \ldots, \hat{p}_n$, followed by the same statistics for the 1-norm, 2-norm, supreme norm for the K differences $\boldsymbol{p} - \hat{\boldsymbol{p}}$ of the true parameter vector and the MLEs, as well as the Jensen-Shannon divergence and Hellinger- and Bhattacharyy-distances of the true frequencies and the estimates $\hat{\boldsymbol{p}}$.

Code example: The following code generates $K = 10000$ regular data sets (see main text) of sample size $N = 150$ with true parameters $\lambda = 1.5$ and frequencies $\boldsymbol{p} = (\frac{1}{4}, \frac{1}{4}, \frac{1}{4}, \frac{1}{4})$, hence there are four lineages. The output is stored as "C:\Documents\simdata4.txt". The extension of the output file will automatically be generated as well as the number 4, which corresponds to the number of lineages. Note that data corresponding to different lineage numbers must not be stored in the same text file! Hence, the extension of the output file is automatically generated.

```
> run1(1.5,c(0.25,0.25,0.25,0.25),150,10000,"C:/Documents/simdata")
```

The function uses the function "pstat" to calculate 1-norm, 2-norm, supreme norm for the K differences $\boldsymbol{p} - \hat{\boldsymbol{p}}$ of the true parameter vector and the MLEs, as well as for the Jensen-Shannon divergence and Hellinger- and Bhattacharyy-distances of the true frequencies and the estimates $\hat{\boldsymbol{p}}$.

2.4 The function "runsim"

The function "runsim($\lambda_{\min}, \lambda_{\max}, \lambda_{\text{inc}}, \boldsymbol{P}, \boldsymbol{N}, K$, path)" is used to run the function "run1" over a range of parameters, namely all combinations of values of λ, frequencies vectors $\boldsymbol{p}$ and sample sizes N specified by the arguments of the function. It will assume every value of λ ranging from $\lambda_{\min}$ to $\lambda_{\max}$ in steps of λ_{inc}, the frequencies are entered in the matrix $\boldsymbol{P}$, where each row corresponds to one frequency distribution $\boldsymbol{p}$ (the number of lineages must be the same for each distribution specified), and the sample sizes are entered in the vector $\boldsymbol{N}$. Moreover, K is an optional argument with the default values $K = 10000$ and "path" is the path to the output text file. The same information as described for the function "run1" will be stored in a text file. Each parameter combination will generate one

line in the output file.

Code example: This code generates $K = 100000$ regular data sets (see main text) for each combination of the following parameters: $\lambda = 0.1, 0.2, 0.3, 0.4, 0.5$, $\boldsymbol{p} = (\frac{1}{4}, \frac{1}{4}, \frac{1}{4}, \frac{1}{4})$, $\boldsymbol{p} = (\frac{1}{2}, \frac{1}{4}, \frac{1}{8}, \frac{1}{8})$, $\boldsymbol{p} = (0.8, 0.1, 0.05, 0.05)$ and $N = 50, 100, 150, 200$. Output is stored as “C:\Documents\simdata4.txt”. The extension of the output file will automatically be generated as well as the number 4, which corresponds to the number of lineages.

```
> Nvec <- c(50,100,150,200)
> pmat <- t(array(c(0.25,0.25,0.25,0.25,0.5,0.25,0.125,0.125,0.8,0.1,0.05,0.05),
    c(4,3)))
> runsim(0.1,0.5,0.1,pmat,Nvec,K=100000,"C:/Documents/simdata")
```

3 Visualizing simulated data using the function “runsim”

The function “Glpe(inpath,n,quant,sta,outpath)” is used to visualize data generated with the function “runsim”. The arguments are as follows: “inpath” is the location of the .txt-file created using “runsim”, and n is the number of lineages used to generate the simulated data set to be visualized. Moreover, “quant” is the quantity to be visualized, it can take the string values “MLE”, “p1”,. . . , “pn”, “1-norm”, “2-norm”, “sup-norm”, “JS-div”, “H-div”, “B-div” corresponding to $\frac{\hat{\lambda}}{1-e^{-\hat{\lambda}}}$, the frequency estimates, the 1-norm, 2-norm, supremum-norm of the differences of the frequency estimates and the true frequencies, the Jensen-Shannon divergence and Hellinger- and Bhattacharyy-distances. The argument “sta” specifies which statistic of the quantity of interest is meant to be visualized. It can take the string values “min”,“Q1”,“me”,“E”,“Q3”,“max”, “var” and “CV”, corresponding to the minimum, 1st quartile, median, mean, 3rd quartile, maximum, variance and coefficient of variation. Finally, “outpath” is the path where the output files are meant to be stored as PDF images. The filenames are generated randomly. The plots will look similar as in the main text. One plot will be generated for each frequency distribution in the input file. Each plot will be grouped by sample size N.

If the input argument for “sta” is “CV”, the argument “quant” must be either one of “MLE”, “p1”,. . . , “pn”. In this case the plots will show the corresponding theoretical coefficients of variation calculated from the Cramér-Rao lower bounds as dashed lines.

The function "runsim" uses the two functions "labels", which assign labels to the imported data and "Lpe" which creates the plots using the package ggplot2.

Code example: This code generates plots saved as PDF-files in the folder "C:\Documents\plots" showing the mean bias of the MLE $\frac{\hat{\lambda}}{1-e^{-\hat{\lambda}}}$ for the data "C:\Documents\simdata4.txt", which was generated using the function "runsim" (see example above) for $n = 4$ lineages:

```
> Glpe("C:/Documents/simdata4.txt",4,"MLE","E","C:/Documents/plots/")
```
