# Supplementary material for: Large and finite sample properties of a maximum-likelihood estimator for multiplicity of infection
Source: PLoS One. 2018 Apr 9;13(4):e0194148. doi: 10.1371/journal.pone.0194148 (PMC5890990; doi:10.1371/journal.pone.0194148)
Supplement: S4 File — Appendix A-F. (PDF) [file pone.0194148.s004.pdf]

S4 Appendix

A Identifiability

The probability of observing an infection with configuration $\boldsymbol{i}$ is

$$Q_{\boldsymbol{i}} = \frac{1}{e^{\lambda} - 1} \prod_{j=1}^{n} (e^{\lambda p_j} - 1)^{i_j} \,. \tag{S1}$$

Proof of Remark 1. Let $Q_{\boldsymbol{i}}^{(1)}$ and $Q_{\boldsymbol{i}}^{(2)}$ denote the distributions (S1) for two parameter vectors $\boldsymbol{\theta}_1, \boldsymbol{\theta}_2 \in \Theta$. Suppose $Q_{\boldsymbol{i}}^{(1)} = Q_{\boldsymbol{i}}^{(2)}$ for all $\boldsymbol{i}$.

First, assume $\boldsymbol{\theta}_1 = (\lambda, \boldsymbol{p}_1)$ and $\boldsymbol{\theta}_2 = (\lambda, \boldsymbol{p}_2)$ with $\boldsymbol{p}_1 \neq \boldsymbol{p}_2$. Without loss of generality let $p_1^{(1)} \neq p_1^{(2)}$, i.e., the first components of $\boldsymbol{p}_1$ and $\boldsymbol{p}_2$ differ. Then clearly $Q_{\boldsymbol{e}_1}^{(1)} \neq Q_{\boldsymbol{e}_1}^{(2)}$, where $\boldsymbol{e}_1 = (1, 0, \ldots, 0)$, a contradiction.

Now, suppose $\boldsymbol{\theta}_1 = (\lambda_1, \boldsymbol{p}_1)$ and $\boldsymbol{\theta}_2 = (\lambda_2, \boldsymbol{p}_2)$ with $\lambda_1 \neq \lambda_2$ and possibly $\boldsymbol{p}_1 \neq \boldsymbol{p}_2$. Without loss of generality let $\lambda_1 < \lambda_2$, $p_1^{(1)} \geq p_1^{(2)}$ and $p_2^{(1)} \leq p_2^{(2)}$. This implies $e^{\lambda_1 p_2^{(1)}} - 1 < e^{\lambda_2 p_2^{(2)}} - 1$. Because $Q_{\boldsymbol{e}_1}^{(1)} = Q_{\boldsymbol{e}_1}^{(2)}$ it follows that $Q_{\boldsymbol{e}_1+\boldsymbol{e}_2}^{(1)} < Q_{\boldsymbol{e}_1+\boldsymbol{e}_2}^{(2)}$ ($\boldsymbol{e}_2 = (0, 1, 0, \ldots, 0)$), a contradiction. Hence, $Q_{\boldsymbol{i}}^{(1)} = Q_{\boldsymbol{i}}^{(2)}$ for all $\boldsymbol{i}$ only if $\boldsymbol{\theta}_1 = \boldsymbol{\theta}_2$. $\square$

B Constraining the data and the parameter space

Here, we derive the lineages' prevalences and the probability of observing irregular data, i.e., $N_k = N$ for at least one k or $\sum_{k=1}^{n} N_k = N$. Remember that a blood sample is represented by a 0-1 vector $\boldsymbol{i} = (i_1, \ldots, i_n)$. The probability of observing $\boldsymbol{i}$ is denoted by $Q_{\boldsymbol{i}}$ and given by (S1).

First, we derive the prevalence of lineage j, i.e., the probability to observe lineage j in a blood sample. Note that by using (S1)

$$\begin{aligned}
1 &= \sum_{\boldsymbol{i} \in \{0,1\}^n \setminus \{\boldsymbol{0}\}} Q_{\boldsymbol{i}} = \sum_{\substack{\boldsymbol{i} \in \{0,1\}^n \setminus \{\boldsymbol{0}\}: \\ i_n = 1}} Q_{\boldsymbol{i}} + \sum_{\substack{\boldsymbol{i} \in \{0,1\}^n \setminus \{\boldsymbol{0}\}: \\ i_n = 0}} Q_{\boldsymbol{i}} \\
&= Q_{\boldsymbol{e}_n} + (e^{\lambda p_n} - 1) \sum_{\substack{\boldsymbol{i} \in \{0,1\}^n \setminus \{\boldsymbol{0}\}: \\ i_n = 0}} Q_{\boldsymbol{i}} + \sum_{\substack{\boldsymbol{i} \in \{0,1\}^n \setminus \{\boldsymbol{0}\}: \\ i_n = 0}} Q_{\boldsymbol{i}} \\
&= \frac{e^{\lambda p_n} - 1}{e^{\lambda} - 1} + e^{\lambda p_n} \sum_{\substack{\boldsymbol{i} \in \{0,1\}^n \setminus \{\boldsymbol{0}\}: \\ i_n = 0}} Q_{\boldsymbol{i}} \\
&= \frac{e^{\lambda p_n} - 1}{e^{\lambda} - 1} + e^{\lambda p_n} \left(1 - \sum_{\substack{\boldsymbol{i} \in \{0,1\}^n \setminus \{\boldsymbol{0}\}: \\ i_n = 1}} Q_{\boldsymbol{i}} \right).
\end{aligned}$$

Hence,

$$\sum_{\substack{\boldsymbol{i}\in\{0,1\}^n\setminus\{\boldsymbol{0}\}:\\ i_n=1}} Q_{\boldsymbol{i}} = \frac{e^{\lambda}(e^{\lambda p_n}-1)}{e^{\lambda p_n}(e^{\lambda}-1)},$$

and by replacing n with j we obtain the probability that lineage j is observed in a blood sample as

$$q_{\{j\}} := q_j = \sum_{\substack{\boldsymbol{i}\in\{0,1\}^n\setminus\{\boldsymbol{0}\}:\\ i_j=1}} Q_{\boldsymbol{i}} = \frac{e^{\lambda}(e^{\lambda p_j}-1)}{e^{\lambda p_j}(e^{\lambda}-1)}. \tag{S2}$$

Note, that p_j is the frequency of lineage j in the population of infective agents and differs from the prevalence q_j. However, in the limit $\lambda \to 0$, these probabilities coincide as is easily seen by applying de l'Hospitals rule. This is not surprising, because in this limit every host is infected by exactly one lineage.

Next, we show by induction that the probability of observing lineages $j_1, \ldots, j_k$ together in a blood sample is given by

$$q_{\{j_1,\ldots,j_k\}} := \sum_{\substack{\boldsymbol{i}\in\{0,1\}^n\setminus\{\boldsymbol{0}\}:\\ i_{j_1}=\ldots=i_{j_k}=1}} Q_{\boldsymbol{i}} = \frac{e^{\lambda}}{e^{\lambda}-1}\prod_{s=1}^{k}(1-e^{-\lambda p_{j_s}}). \tag{S3}$$

This probability corresponds to the prevalence of the combination of lineages $j_1, \ldots, j_k$.

If $k = 1$ the formulas (S3) and (S2) obviously coincide. By relabelling it suffices to show that (S3) holds for lineages $n-k, \ldots, n$. Assume $q_{\{n-k+1,\ldots,n\}}$ is given by (S3). Hence,

$$\begin{aligned}
q_{\{n-k+1,\ldots,n\}} &= \sum_{\substack{\boldsymbol{i}\in\{0,1\}^n\setminus\{\boldsymbol{0}\}:\\ i_{n-k+1}=\ldots=i_n=1}} Q_{\boldsymbol{i}} = \sum_{\substack{\boldsymbol{i}\in\{0,1\}^{n-k}:\\ i_{n-k}=1}} Q_{(\boldsymbol{i},1,\ldots,1)} + \sum_{\substack{\boldsymbol{i}\in\{0,1\}^{n-k}:\\ i_{n-k}=0}} Q_{(\boldsymbol{i},1,\ldots,1)} \\
&= (e^{\lambda p_{n-k}}-1) \sum_{\substack{\boldsymbol{i}\in\{0,1\}^{n-k}:\\ i_{n-k}=0}} Q_{(\boldsymbol{i},1,\ldots,1)} + \sum_{\substack{\boldsymbol{i}\in\{0,1\}^{n-k}:\\ i_{n-k}=0}} Q_{(\boldsymbol{i},1,\ldots,1)} \\
&= e^{\lambda p_{n-k}} \sum_{\substack{\boldsymbol{i}\in\{0,1\}^{n-k}:\\ i_{n-k}=0}} Q_{(\boldsymbol{i},1,\ldots,1)} \\
&= e^{\lambda p_{n-k}} \Bigg(q_{\{n-k+1,\ldots,n\}} - \sum_{\substack{\boldsymbol{i}\in\{0,1\}^{n-k}:\\ i_{n-k}=1}} Q_{(\boldsymbol{i},1,\ldots,1)} \Bigg) \\
&= e^{\lambda p_{n-k}} \big(q_{\{n-k+1,\ldots,n\}} - q_{\{n-k,\ldots,n\}} \big).
\end{aligned}$$

Hence,

$$q_{\{n-k,\ldots,n\}} = \frac{e^{\lambda}}{e^{\lambda}-1}\prod_{j=n-k}^{n}(1-e^{-\lambda p_j})$$

follows, which proves (S3).

Now, the probability that $N_k = N$ for at least on k is found by a standard inclusion exclusion argument and is given by

$$\begin{aligned}\sum_{A\subseteq\{1,\ldots,n\}}(-1)^{|A|+1}\Big(\sum_{\substack{\boldsymbol{i}\in\{0,1\}^n\setminus\{\boldsymbol{0}\}:\\ i_k=1 \text{ if } k\in A}} Q_{\boldsymbol{i}}\Big)^N &= \sum_{\substack{A\subseteq\{1,\ldots,n\}\\ A\neq\emptyset}}(-1)^{|A|+1}q_A^N \\ &= \frac{1}{(1-e^{-\lambda})^N}\sum_{\substack{A\subseteq\{1,\ldots,n\}\\ A\neq\emptyset}}(-1)^{|A|+1}\prod_{j\in A}(1-e^{-\lambda p_j})^N \\ &= \frac{1}{(1-e^{-\lambda})^N}\left(1-\prod_{j=1}^{n}\left(1-(1-e^{-\lambda p_j})^N\right)\right).\end{aligned} \tag{S4}$$

Next, we will derive the probability of observing only single infections. A single infection with lineage k is represented by the standard base vectors $\boldsymbol{e}_k$. The probability of a single infection is therefore $Q_{\boldsymbol{e}_1}+\ldots+Q_{\boldsymbol{e}_n}$. Hence, the probability of observing only single infections is given by $\big(\sum_{k=1}^{n} Q_{\boldsymbol{e}_k}\big)^N$.

Summarizing, the probability that $N_k = N$ for at least one k or $\sum_{k=1}^{n} N_k = N$, is given by

$$q := \frac{1}{(1-e^{-\lambda})^N}\left(1-\prod_{j=1}^{n}\left(1-(1-e^{-\lambda p_j})^N\right)\right)+\Big(\sum_{j=1}^{n} Q_{\boldsymbol{e}_j}\Big)^N-\sum_{j=1}^{n} Q_{\boldsymbol{e}_j}^N\,,$$

where the probabilities that all samples are only infected by lineage j, i.e., $Q_{\boldsymbol{e}_j}^N$, are subtracted because they are accounted for in both irregular cases, i.e., N single infections with the same lineage ($N_j = N$ for exactly one j implying $\sum_{k=1}^{n} N_k = N$).

Clearly, the probability q vanishes as $N\to\infty$. However, if N and λ are small and the lineage frequencies are very skewed, it might be rather larger.

Next we present the proof of Result 1.

Proof of Result 1. For regular data a unique MLE $(\hat\lambda,\hat{\boldsymbol{p}})$ exists and $\lambda = 0$ is neither the MLE nor the true parameter (cf. [1]).

Because $\hat\Theta$ is compact, $L(\lambda,\boldsymbol{p})$ attains a global maximum at $(\tilde\lambda,\tilde{\boldsymbol{p}})\in\hat\Theta$. If $\hat\lambda\notin[\lambda_{\min},\lambda_{\max}]$, then $(\hat\lambda,\hat{\boldsymbol{p}})\notin\hat\Theta$. Hence, $(\tilde\lambda,\tilde{\boldsymbol{p}})\neq(\hat\lambda,\hat{\boldsymbol{p}})$. Therefore, $\tilde\lambda=\lambda_{\min}$ or $\tilde\lambda=\lambda_{\max}$ must hold.

Let $c = L(\hat\lambda,\hat{\boldsymbol{p}})-L(\tilde\lambda,\tilde{\boldsymbol{p}})>0$. The parameter vector $(\tilde\lambda,\tilde{\boldsymbol{p}})$ maximizes $L(\lambda,\boldsymbol{p})$ subject to the equality constraint $L(\hat\lambda,\hat{\boldsymbol{p}})-L(\lambda,\boldsymbol{p})-c=0$. This corresponds exactly to finding the profile-likelihood confidence interval $(\underline{\lambda},\overline{\lambda})$ for $\hat\lambda$ at the $(1-\alpha)$-level, where α is chosen such that $2c$ is the $(1-\alpha)$-quantile of the χ^2-distribution with 1 degree of freedom.

As shown in [1], unique confidence points $\underline{\lambda}<\hat\lambda<\overline{\lambda}$ exists, implying $\tilde\lambda=\underline{\lambda}$ or $\tilde\lambda=\overline{\lambda}$.

Assume $\hat\lambda<\lambda_{\min}$. Then $\tilde\lambda=\lambda_{\max}$ is impossible, because the uniqueness of $\overline{\lambda}>\hat\lambda$ maximizing the profile-likelihood subject to the constraint $L(\hat\lambda,\hat{\boldsymbol{p}})-L(\lambda,\boldsymbol{p})=c$ would imply $L(\hat\lambda,\hat{\boldsymbol{p}})-\max_{\boldsymbol{p}} L(\lambda_{\min},\boldsymbol{p})<c$. The latter is a contradiction to $(\tilde\lambda,\tilde{\boldsymbol{p}})$ being the

point, where the log-likelihood function attains its global maximum on $\hat{\Theta}$. Thus, $\tilde{\lambda} = \lambda_{\min}$. The same reasoning yields $\tilde{\lambda} = \lambda_{\max}$ if $\hat{\lambda} > \lambda_{\max}$.

In the case that only one lineage is found in the data, i.e., $\sum_{k=1}^{n} N_k = N$ and $N_j = N$ for some j, it is immediately clear from the log-likelihood function that any estimate of the form $(\lambda, \boldsymbol{e}_j)$ is equally likely. Hence, we further assume that at least two lineages are present in the data.

If $\sum_{k=1}^{n} N_k = N$, $\hat{\lambda} = 0$ is the MLE. The argument with the profile-likelihood still applies (however only the upper confidence point $\overline{\lambda}$ exists), yielding $\tilde{\lambda} = \lambda_{\min}$.

Next, we consider λ as a fixed constant and maximize the log-likelihood function (eq. 3 in the main text) over the simplex. It is more convenient to introduce a nuisance parameter β and maximize the function

$$\Lambda(\boldsymbol{p}, \beta) = L(\lambda, \boldsymbol{p}) - \beta\Big(1 - \sum_{k=1}^{n} p_k\Big),$$

over $\mathbb{R}^{n+1}$ because

$$\frac{\partial \Lambda}{\partial \beta} = 0 \quad \Leftrightarrow \quad \sum_{i=1}^{n} p_i = 1.$$

The equations $\frac{\partial \Lambda}{\partial p_k} = 0$ yields $N_k \frac{\lambda e^{\lambda p_k}}{e^{\lambda p_k} - 1} = \beta$ for all k (cf. eq. S17b in Appendix D below). Solving this equation with respect to p_k yields

$$p_k = -\frac{1}{\lambda} \log\Big(1 - \frac{\lambda N_k}{\beta}\Big),$$

which is in essence eq. 6 in the main text. Substituting this into $\sum_{k=1}^{n} p_k = 1$, yields after a little rearrangement,

$$0 = \lambda + \sum_{k=1}^{n} \log\Big(1 - \frac{\lambda N_k}{\beta}\Big) =: f(\beta).$$

Clearly, $f(\beta)$ is defined only for $\beta > \max_k \lambda N_k =: \beta_0$, $\lim_{\beta \to \beta_0} f(\beta) = -\infty$ and $\lim_{\beta \to \infty} f(\beta) = \lambda$. Taking the derivative yields

$$f'(\beta) = \frac{1}{\beta} \sum_{k=1}^{n} \frac{\lambda N_k}{\beta - \lambda N_k} > 0.$$

Thus, f is strictly monotonically increasing and consequently $f(\hat{\beta}) = 0$ for exactly one $\hat{\beta} > \beta_0$. This solution is found by applying Newton's method, which yields exactly eq. 6b in the main text for $\lambda = \lambda_{\min}$. Choosing $\lambda = \lambda_{\max}$ yields the rest of the proof.

□

C Fisher information matrix

For the derivations here it is convenient to eliminate one redundant parameter by substituting $p_n = 1 - \sum_{k=1}^{n-1} p_k$. The first order derivatives of the log-likelihood function

are

$$\frac{\partial L}{\partial \lambda} = -N\frac{e^{\lambda}}{e^{\lambda}-1} + \sum_{k=1}^{n} N_k \frac{p_k e^{\lambda p_k}}{e^{\lambda p_k}-1}\,, \tag{S5a}$$

$$\frac{\partial L}{\partial p_k} = N_k\frac{\lambda e^{\lambda p_k}}{e^{\lambda p_k}-1} - N_n\frac{\lambda e^{\lambda p_n}}{e^{\lambda p_n}-1}\,, \tag{S5b}$$

and the second order derivatives are

$$\frac{\partial^2 L}{\partial \lambda^2} = N\frac{e^{\lambda}}{(e^{\lambda}-1)^2} - \sum_{k=1}^{n} N_k \frac{p_k^2 e^{\lambda p_k}}{(e^{\lambda p_k}-1)^2}\,, \tag{S6a}$$

$$\frac{\partial^2 L}{\partial \lambda \partial p_k} = \frac{\partial^2 L}{\partial p_k \partial \lambda} = N_k\frac{e^{\lambda p_k}}{e^{\lambda p_k}-1}\Big(1-\frac{\lambda p_k}{e^{\lambda p_k}-1}\Big) - N_n\frac{e^{\lambda p_n}}{e^{\lambda p_n}-1}\Big(1-\frac{\lambda p_n}{e^{\lambda p_n}-1}\Big)\,, \tag{S6b}$$

$$\frac{\partial^2 L}{\partial p_k^2} = -\frac{N_k\lambda^2 e^{\lambda p_k}}{(e^{\lambda p_k}-1)^2} - \frac{N_n\lambda^2 e^{\lambda p_n}}{(e^{\lambda p_n}-1)^2}\,, \tag{S6c}$$

$$\frac{\partial^2 L}{\partial p_k \partial p_j} = -\frac{N_n\lambda^2 e^{\lambda p_n}}{(e^{\lambda p_n}-1)^2} \quad \text{for } k \neq j\,. \tag{S6d}$$

Clearly, the third-order derivatives exist for any admissible parameter value $\boldsymbol{\vartheta} \in \tilde{\Theta}$ and could be calculated analogously. Obviously, these are linear in N and the N_k's. Therefore, by the theorem of maximum and minimum we obtain:

Remark 1 *The third-order derivatives of the log-likelihood function satisfy that $\frac{1}{N}\frac{\partial^3 L}{\partial \boldsymbol{\vartheta}^3}$ is uniformly bounded on any compact subset $\hat{\Theta} \subsetneq \tilde{\Theta}$.*

Notably, $\tilde{\Theta}$ is not compact and the third-order derivatives are indeed not bounded on $\tilde{\Theta}$. In order to derive the information matrix and prove its properties we need the following lemma.

Lemma 1 *The following identities hold for k, $\ell = 1, \ldots, n$:*

$$\sum_{\boldsymbol{i}\in\{0,1\}^n\setminus\{\boldsymbol{0}\}} i_k Q_{\boldsymbol{i}} = \sum_{\boldsymbol{i}\in\{0,1\}^n\setminus\{\boldsymbol{0}\}} i_k^2 Q_{\boldsymbol{i}} = \frac{1-e^{-\lambda p_k}}{1-e^{-\lambda}}\,, \tag{S7a}$$

$$\sum_{\boldsymbol{i}\in\{0,1\}^n\setminus\{\boldsymbol{0}\}} i_k i_\ell Q_{\boldsymbol{i}} = \frac{(1-e^{-\lambda p_k})(1-e^{-\lambda p_\ell})}{1-e^{-\lambda}} \quad \textit{for } k \neq \ell\,, \tag{S7b}$$

$$\mathbb{E}\, N_k = N\frac{1-e^{-\lambda p_k}}{1-e^{-\lambda}}\,, \tag{S7c}$$

$$\mathbb{E}\, N_k N_\ell = \frac{(1-e^{-\lambda p_k})(1-e^{-\lambda p_\ell})}{1-e^{-\lambda}}\left(\frac{N^2-N}{1-e^{-\lambda}}+N\right) + \delta_{k\ell} N \frac{e^{-\lambda p_k}(1-e^{-\lambda p_k})}{1-e^{-\lambda}}\,, \tag{S7d}$$

where $\delta_{k\ell}$ denotes the Kronecker delta.

Proof. Clearly, (S7a) is equivalent to (S2). The second identity is a special case of (S3).

Denoting a 0-1 vector by $\boldsymbol{i} = (i_1, \ldots, i_n)$ and by $n_{\boldsymbol{i}}$ the number of blood samples corresponding to $\boldsymbol{i}$ we clearly have $N_k = \sum_{\boldsymbol{i}\in\{0,1\}^n\setminus\{\boldsymbol{0}\}} i_k n_{\boldsymbol{i}}$. Moreover, the $n_{\boldsymbol{i}}$ are

multinomially distributed with parameters N and $Q_{\boldsymbol{i}}$, where $\boldsymbol{i} \in \{0,1\}^n \setminus \{\mathbf{0}\}$. Therefore, $\mathbb{E}\, n_{\boldsymbol{i}} = NQ_{\boldsymbol{i}}$ and hence

$$\mathbb{E}\, N_k = \mathbb{E}\Bigg(\sum_{\boldsymbol{i}\in\{0,1\}^n\setminus\{\mathbf{0}\}} i_k n_{\boldsymbol{i}}\Bigg) = \sum_{\boldsymbol{i}\in\{0,1\}^n\setminus\{\mathbf{0}\}} i_k\, \mathbb{E}\, n_{\boldsymbol{i}} = N \sum_{\boldsymbol{i}\in\{0,1\}^n\setminus\{\mathbf{0}\}} i_k Q_{\boldsymbol{i}} = N\frac{1-e^{-\lambda p_k}}{1-e^{-\lambda}}$$

according to the first identity.

From the multinomial distribution of the $n_{\boldsymbol{i}}$ we obtain $\mathbb{E}\, n_{\boldsymbol{i}} n_{\boldsymbol{j}} = (N^2-N)Q_{\boldsymbol{i}}Q_{\boldsymbol{j}}$ for $\boldsymbol{i} \neq \boldsymbol{j}$ and $\mathbb{E}\, n_{\boldsymbol{i}}^2 = (N^2-N)Q_{\boldsymbol{i}}^2 + NQ_{\boldsymbol{i}}$. Next, we aim to derive $\mathbb{E}\, N_k N_\ell$. Hence,

$$\begin{aligned}
\mathbb{E}\, N_k N_\ell =& \mathbb{E}\Bigg(\sum_{\boldsymbol{i},\boldsymbol{j}\in\{0,1\}^n\setminus\{\mathbf{0}\}} i_k j_\ell n_{\boldsymbol{i}} n_{\boldsymbol{j}}\Bigg) = \sum_{\boldsymbol{i},\boldsymbol{j}\in\{0,1\}^n\setminus\{\mathbf{0}\}} i_k j_\ell\, \mathbb{E}\, n_{\boldsymbol{i}} n_{\boldsymbol{j}} \\
=&(N^2-N) \sum_{\boldsymbol{i},\boldsymbol{j}\in\{0,1\}^n\setminus\{\mathbf{0}\}} i_k j_\ell Q_{\boldsymbol{i}} Q_{\boldsymbol{j}} + N \sum_{\boldsymbol{i}\in\{0,1\}^n\setminus\{\mathbf{0}\}} i_k i_\ell Q_{\boldsymbol{i}} \\
=&(N^2-N)\Bigg(\sum_{\boldsymbol{i}\in\{0,1\}^n\setminus\{\mathbf{0}\}} i_k Q_{\boldsymbol{i}}\Bigg)\Bigg(\sum_{\boldsymbol{j}\in\{0,1\}^n\setminus\{\mathbf{0}\}} j_\ell Q_{\boldsymbol{j}}\Bigg) + N \sum_{\boldsymbol{i}\in\{0,1\}^n\setminus\{\mathbf{0}\}} i_k i_\ell Q_{\boldsymbol{i}}\,.
\end{aligned}$$

Using (S7a)-(S7c) we obtain

$$\mathbb{E}\, N_k N_\ell = \frac{(1-e^{-\lambda p_k})(1-e^{-\lambda p_\ell})}{1-e^{-\lambda}}\left(\frac{N^2-N}{1-e^{-\lambda}} + N\right)$$

for $k \neq \ell$ and

$$\begin{aligned}
\mathbb{E}\, N_k^2 &= \frac{1-e^{-\lambda p_k}}{1-e^{-\lambda}}\left((N^2-N)\frac{1-e^{-\lambda p_k}}{1-e^{-\lambda}} + N\right) \\
&= \frac{(1-e^{-\lambda p_k})^2}{1-e^{-\lambda}}\left(\frac{N^2-N}{1-e^{-\lambda}} + N\right) + N\frac{(1-e^{-\lambda p_k})^2}{1-e^{-\lambda}}\left(\frac{1}{1-e^{-\lambda p_k}} - 1\right) \\
&= \frac{(1-e^{-\lambda p_k})^2}{1-e^{-\lambda}}\left(\frac{N^2-N}{1-e^{-\lambda}} + N\right) + N\frac{e^{-\lambda p_k}(1-e^{-\lambda p_k})}{1-e^{-\lambda}}
\end{aligned}$$

for $k = \ell$, finishing the proof. □

Lemma 1 enables us to derive the information matrix immediately from (S6), which is presented in Result 2 in the Main Text. Moreover, we are now able to prove an important regulatory condition.

Theorem 1 *The Fisher information matrix satisfies*

$$I = -\,\mathbb{E}\,\frac{\partial^2 L}{\partial \boldsymbol{\vartheta}^2} = \mathbb{E}\left(\left(\frac{\partial L}{\partial \boldsymbol{\vartheta}}\right)^T \cdot \left(\frac{\partial L}{\partial \boldsymbol{\vartheta}}\right)\right). \tag{S8}$$

Proof. From (S5) one obtains

$$\begin{aligned}
\frac{\partial L}{\partial \lambda}\frac{\partial L}{\partial \lambda} =& N^2\frac{1}{(1-e^{-\lambda})^2} - \frac{2N}{1-e^{-\lambda}}\sum_{k=1}^{n} N_k \frac{p_k}{1-e^{-\lambda p_k}} \\
&+ \sum_{k,\ell=1}^{n} N_k N_\ell \frac{p_k p_\ell}{(1-e^{-\lambda p_k})(1-e^{-\lambda p_\ell})}\,.
\end{aligned}$$

Hence, Lemma 1 yields

$$\begin{aligned}\mathbb{E}\,\frac{\partial L}{\partial\lambda}\frac{\partial L}{\partial\lambda} =&\frac{N^2}{(1-e^{-\lambda})^2}-\frac{2N}{1-e^{-\lambda}}\sum_{k=1}^{n}N\frac{p_k}{1-e^{-\lambda}}+\sum_{k,\ell=1}^{n}\frac{p_kp_\ell(N^2-N)}{(1-e^{-\lambda})^2}\\ &+\sum_{k,\ell=1}^{n}\frac{p_kp_\ell N}{1-e^{-\lambda}}+\sum_{k=1}^{n}\frac{p_k^2Ne^{-\lambda p_k}}{(1-e^{-\lambda p_k})(1-e^{-\lambda})}\\ =&\frac{N^2}{(1-e^{-\lambda})^2}-\frac{-2N^2}{(1-e^{-\lambda})^2}+\frac{N^2-N}{(1-e^{-\lambda})^2}+\frac{N}{1-e^{-\lambda}}+\frac{N}{1-e^{-\lambda}}\sum_{k=1}^{n}\frac{p_k^2e^{-\lambda p_k}}{1-e^{-\lambda p_k}}\\ =&\frac{N}{1-e^{-\lambda}}\left(1-\frac{1}{1-e^{-\lambda}}\right)+\frac{Ne^{\lambda}}{e^{\lambda}-1}\sum_{k=1}^{n}\frac{p_k^2}{e^{\lambda p_k}-1}\\ =&-\frac{Ne^{\lambda}}{(e^{\lambda}-1)^2}+\frac{Ne^{\lambda}}{e^{\lambda}-1}\sum_{k=1}^{n}\frac{p_k^2}{e^{\lambda p_k}-1}=I_{1,1}\,.\end{aligned}$$

Straightforward calculation yields,

$$\begin{aligned}\frac{\partial L}{\partial\lambda}\frac{\partial L}{\partial p_k}=&-\frac{N\lambda}{1-e^{-\lambda}}\left(\frac{N_k}{1-e^{-\lambda p_k}}-\frac{N_n}{1-e^{-\lambda p_k}}\right)\\ &+\sum_{j=1}^{n}\frac{\lambda p_j}{1-e^{-\lambda p_j}}\left(\frac{N_jN_k}{1-e^{-\lambda p_k}}-\frac{N_jN_n}{1-e^{-\lambda p_n}}\right).\end{aligned}$$

By using Lemma 1 we obtain

$$\begin{aligned}\mathbb{E}\,\frac{\partial L}{\partial\lambda}\frac{\partial L}{\partial p_k}=&\frac{-N^2\lambda}{(1-e^{-\lambda})^2}(1-1)+\frac{\lambda}{1-e^{-\lambda}}\left(\frac{N^2-N}{1-e^{-\lambda}}+N\right)\sum_{j=1}^{n}p_j(1-1)\\ &+\frac{N\lambda p_ke^{-\lambda p_k}}{(1-e^{-\lambda p_k})(1-e^{-\lambda})}-\frac{N\lambda p_ne^{-\lambda p_n}}{(1-e^{-\lambda p_n})(1-e^{-\lambda})}\\ =&\frac{N\lambda}{1-e^{-\lambda}}\left(\frac{p_k}{e^{\lambda p_k}-1}-\frac{p_n}{e^{\lambda p_n}-1}\right)=I_{1,k+1}=I_{k+1,1}\,.\end{aligned}$$

Next, note that

$$\frac{\partial L}{\partial p_k}\frac{\partial L}{\partial p_k}=\lambda^2\left(\frac{N_k^2}{(1-e^{-\lambda p_k})^2}-\frac{2N_kN_n}{(1-e^{-\lambda p_k})(1-e^{-\lambda p_n})}+\frac{N_n^2}{(1-e^{-\lambda p_n})^2}\right)$$

yielding

$$\begin{aligned}\mathbb{E}\,\frac{\partial L}{\partial p_k}\frac{\partial L}{\partial p_k}=&\frac{\lambda^2(1-2-1)}{1-e^{-\lambda}}\left(\frac{N^2-N}{1-e^{-\lambda}}+N\right)^2+\frac{\lambda^2N}{1-e^{-\lambda}}\left(\frac{e^{-\lambda p_k}}{1-e^{-\lambda p_k}}+\frac{e^{-\lambda p_n}}{1-e^{-\lambda p_n}}\right)\\ =&\frac{\lambda^2N}{1-e^{-\lambda}}\left(\frac{1}{e^{\lambda p_k}-1}+\frac{1}{e^{\lambda p_n}-1}\right)=I_{k+1,k+1}\,.\end{aligned}$$

Furthermore, we have

$$\begin{aligned}\frac{\partial L}{\partial p_k}\frac{\partial L}{\partial p_j}=&\frac{N_kN_j\lambda^2}{(1-e^{-\lambda p_k})(1-e^{-\lambda p_j})}\\ &+\frac{1}{1-e^{-\lambda p_n}}\lambda^2\left(\frac{N_n^2}{1-e^{-\lambda p_n}}-\frac{N_kN_n}{1-e^{-\lambda p_k}}-\frac{N_jN_n}{1-e^{-\lambda p_j}}\right)\end{aligned}$$

so that

$$\mathbb{E}\,\frac{\partial L}{\partial p_k}\frac{\partial L}{\partial p_j} = \left(\frac{N^2-N}{1-e^{-\lambda}}+N\right)\frac{\lambda^2}{1-e^{-\lambda}}(1+1-1-1)+\frac{\lambda^2 N}{(e^{\lambda p_k}-1)(1-e^{-\lambda})}$$
$$= I_{k+1,j+1}\,.$$

Finally, we derive

$$\frac{\partial L}{\partial p_k}\frac{\partial L}{\partial p_j} = \frac{N_k N_j \lambda^2}{(1-e^{-\lambda p_k})(1-e^{-\lambda p_j})} + \frac{1}{1-e^{-\lambda p_n}}\lambda^2\left(\frac{N_n^2}{1-e^{-\lambda p_n}}-\frac{N_k N_n}{1-e^{-\lambda p_k}}-\frac{N_j N_n}{1-e^{-\lambda p_j}}\right)$$

which gives

$$\mathbb{E}\,\frac{\partial L}{\partial p_k}\frac{\partial L}{\partial p_j} = \left(\frac{N^2-N}{1-e^{-\lambda}}+N\right)\frac{\lambda^2}{1-e^{-\lambda}}(1+1-1-1)+\frac{\lambda^2 N}{(e^{\lambda p_k}-1)(1-e^{-\lambda})}$$
$$= I_{k+1,j+1}\,,$$

and finishes the proof. □

To prove positive definiteness of the information matrix, we need the following lemma first.

Lemma 2 *For $\lambda > 0$ and $0 < p < 1$ the inequality $\frac{e^{\lambda p}-1}{e^{\lambda}-1} < p$ holds, for $p = 0,\, 1$ equality holds.*

Proof. The statement is clear for $p = 0,\, 1$. Let $x = e^{\lambda}$, hence $x > 1$. Moreover, let $f(x,p) := \frac{x^p-1}{x-1}$ for $x > 1$ and $0 \le p \le 1$. We have $\lim_{x\to 0} f(x,p) = p$ by de l'Hospital's rule, $\lim_{x\to\infty} f(x,p) = 0$ for $0 \le p < 1$ and $\lim_{x\to\infty} f(x,1) = 1$. Hence, the prove the statement it suffices to show that $f(x,p)$ is strictly monotonically decreasing for any fixed $p \in (0,1)$.

We have $\frac{\partial f}{\partial x} = \frac{1-px^{p-1}-(1-p)x^p}{(x-1)^2}$. To show that $\frac{\partial f}{\partial x} < 0$ it suffices to consider the numerator. Let $g(p) = 1 - px^{p-1} - (1-p)x^p$ for any fixed x. We have $g'(p) = -(1-x)x^{p-1} - x^{p-1}(x(1-p)+p)\log x$. Clearly, $g(0) = g(1) = 0$. Solving $g'(p) = 0$ yields the unique solution $\hat{p} = -\frac{1-x+x\log x}{(1-x)\log x}$. Since $g(0) = g(1) = 0$ the mean value theorem implies $0 < \hat{p} < 1$. Moreover, a local minimum is attained at $\hat{p}$, because $g'(1) = x - 1 - \log x > 0$ for $x > 1$. Hence, $g(p) < 0$ for $0 < p < 1$. Therefore, $\frac{\partial f}{\partial x} < 0$ is shown, which finishes the proof.

□

Now, the next important property of the information matrix can be proved.

Theorem 2 *The Fisher information is positive definite.*

Proof. Positive definiteness is proved by the Ruth-Hurwitz criterium. The (leading) minors of the Fisher information need to have positive determinants. For convenience we define the leading minors from right to left. The first leading minor, $I_{n,n}$, is clearly positive. The kth leading minor ($k < n$), by first adding a redundant row and column and then successively using that adding multiples of columns does not change the

determinant, becomes

$$\det M_k = \begin{vmatrix} I_{n-k+1,n-k+1} & \dots & I_{n-k+1,n} \\ \vdots & \ddots & \vdots \\ I_{n,n-k+1} & \dots & I_{n,n} \end{vmatrix} = \begin{vmatrix} I_{n-k+1,n-k+1} & \dots & I_{n-k+1,n} & 0 \\ \vdots & \ddots & \vdots & \vdots \\ I_{n,n-k+1} & \dots & I_{n,n} & 0 \\ 0 & \dots & 0 & 1 \end{vmatrix}$$

$$= \frac{1}{a} \begin{vmatrix} I_{n-k+1,n-k+1} & \dots & I_{n-k+1,n} & 0 \\ \vdots & \ddots & \vdots & \vdots \\ I_{n,n-k+1} & \dots & I_{n,n} & 0 \\ 0 & \dots & 0 & a \end{vmatrix} = \frac{1}{a} \begin{vmatrix} I_{n-k+1,n-k+1} & \dots & I_{n-k+1,n} & a \\ \vdots & \ddots & \vdots & \vdots \\ I_{n,n-k+1} & \dots & I_{n,n} & a \\ 0 & \dots & 0 & a \end{vmatrix}$$

$$= \frac{1}{a} \begin{vmatrix} I_{n-k+1,n-k+1} - a & \dots & I_{n-k+1,n} - a & a \\ \vdots & \ddots & \vdots & \vdots \\ I_{n,n-k+1} - a & \dots & I_{n,n} - a & a \\ -a & \dots & -a & a \end{vmatrix}.$$

By choosing $a = \frac{N\lambda^2}{1-e^{-\lambda}} \frac{1}{e^{\lambda p_n}-1}$ we obtain

$$\det M_k = \frac{1}{a} a^k \begin{vmatrix} D & B^T \\ -B & 1 \end{vmatrix}, \tag{S9}$$

where $D = (e^{\lambda p_n} - 1)\,\mathrm{diag}((e^{\lambda p_{n-k+1}} - 1)^{-1}, \dots, (e^{\lambda p_{n-1}} - 1)^{-1})$, and $B = (1, \dots, 1)$. Successively adding multiples of all but the last to the last row yields an upper triangular matrix. Hence, one obtains

$$\det M_k = a^{k-1} \left(1 + \sum_{j=n-k+1}^{n-1} \frac{e^{\lambda p_j} - 1}{e^{\lambda p_n} - 1}\right) \prod_{j=n-k+1}^{n} \frac{e^{\lambda p_n} - 1}{e^{\lambda p_j} - 1} > 0. \tag{S10}$$

By similar steps as above, the determinant of the Fisher information is

$$\det I = \frac{1}{a\lambda} \begin{vmatrix} \lambda I_{1,1} + b p_n & I_{1,2} - b & \dots & I_{1,n} - b & b \\ \lambda I_{2,1} + a p_n & I_{2,2} - a & \dots & I_{2,n} - a & a \\ \vdots & \vdots & \ddots & \vdots & \vdots \\ \lambda I_{n,1} + a p_n & I_{n,2} - a & \dots & I_{n,n} - a & a \\ a p_n & -a & \dots & -a & a \end{vmatrix}. \tag{S11}$$

Choosing $a = \frac{N\lambda^2}{1-e^{-\lambda}} \frac{1}{e^{\lambda p_n}-1}$ and $b = -\frac{N\lambda p_n}{1-e^{-\lambda}} \frac{1}{e^{\lambda p_n}-1}$ yields

$$\det I = \frac{a^{n-1}}{\lambda^2} \begin{vmatrix} f & V & -p_n \\ V^T & D & B^T \\ p_n & -B & 1 \end{vmatrix}, \tag{S12}$$

where $D = (e^{\lambda p_n} - 1)\,\mathrm{diag}((e^{\lambda p_1} - 1)^{-1}, \dots, (e^{\lambda p_{n-1}} - 1)^{-1})$, $B = (1, \dots, 1)$, $V = (e^{\lambda p_n} - 1)(p_1(e^{\lambda p_1} - 1)^{-1}, \dots, p_{n-1}(e^{\lambda p_{n-1}} - 1)^{-1})$, and $f = -\frac{e^{\lambda p_n}-1}{e^{\lambda}-1} + \sum_{k=1}^{n-1} \frac{p_k^2(e^{\lambda p_n}-1)}{e^{\lambda p_k}-1}$. Sunstracting p_k times column $k+1$ from the first

column for $k = 1, \dots n-1$ gives

$$\frac{\lambda^2}{a^{n-1}} \det I = \begin{vmatrix} g & V & -p_n \\ \mathbf{0}^T & D & B^T \\ \sum_{k=1}^{n} p_k & -B & 1 \end{vmatrix} = \begin{vmatrix} g & V & -p_n \\ \mathbf{0}^T & D & B^T \\ 1 & -B & 1 \end{vmatrix}, \tag{S13}$$

where $g = -\frac{e^{\lambda p_n}-1}{e^{\lambda}-1}$. Subtracting $\frac{e^{\lambda p_k}-1}{e^{\lambda p_n}-1}$ times colum $k+1$ from the last column yields

$$\frac{\lambda^2}{a^{n-1}} \det I = \begin{vmatrix} g & V & -\sum_{k=1}^{n} p_k \\ \mathbf{0}^T & D & \mathbf{0}^T \\ 1 & -B & h \end{vmatrix} = \begin{vmatrix} g & \mathbf{0} & -1 \\ \mathbf{0}^T & D & \mathbf{0}^T \\ 1 & -B & h \end{vmatrix} = (-1)^{n-1} \begin{vmatrix} g & \mathbf{0} & -1 \\ 1 & -B & h \\ \mathbf{0}^T & D & \mathbf{0}^T \end{vmatrix} \tag{S14}$$

$$= (-1)^{2n-2} \begin{vmatrix} g & -1 & \mathbf{0} \\ 1 & h & -B \\ \mathbf{0}^T & \mathbf{0}^T & D \end{vmatrix} = (gh+1) \prod_{k=1}^{n-1} \frac{e^{\lambda p_k}-1}{e^{\lambda p_n}-1}, \tag{S15}$$

where $h = 1 + \sum_{k=1}^{n-1} \frac{e^{\lambda p_k}-1}{e^{\lambda p_n}-1} = \sum_{k=1}^{n} \frac{e^{\lambda p_k}-1}{e^{\lambda p_n}-1}$. We have $gh + 1 = 1 - \sum_{k=1}^{n} \frac{e^{\lambda p_k}-1}{e^{\lambda}-1} > 1 - \sum_{k=1}^{n} p_k = 0$ according to Lemma 2. Thus, $\det I > 0$, implying that the Fisher information is positive definite.

□

D Derivation of the Cramér-Rao lower bound

To derive the Fisher information and especially its properties it was convenient to reduce the parameter space Θ to the lower dimensional parameter space $\tilde{\Theta}$ by eliminating a redundant parameter. However, inverting the information given by Result 2 is cumbersome. To derive the Cramér-Rao lower bound, it is more convenient to embed the parameter space Θ into a higher dimensional space by introducing a nuisance parameter. Namely, consider the log-likelihood function

$$\Lambda = \Lambda(\boldsymbol{p}, \lambda, \beta) = \Lambda(\boldsymbol{p}, \lambda, \beta | \boldsymbol{X}) = L(\lambda, \boldsymbol{p} | \boldsymbol{X}) + \beta(1 - \sum_{k=1}^{n} p_k). \tag{S16}$$

Note that $\Lambda(\boldsymbol{p}, \lambda, \beta) = L(\lambda, \boldsymbol{p}|\boldsymbol{X})$ since the term that has been added vanishes for any admissible set of parameters. The advantage of Λ is that $\boldsymbol{p}$ can be regarded as an element of $\mathbb{R}^n$ rather than of the $(n-1)$-dimensional simplex when calculating the derivatives. However, the parameter space itself does not change. Moreover, β is regarded as a scalar, although we impose that the true value is given by $\beta = N\frac{\lambda e^{\lambda}}{e^{\lambda}-1}$.

Straightforward calculation yields

$$\frac{\partial \Lambda}{\partial \lambda} = -N\frac{e^{\lambda}}{e^{\lambda}-1} + \sum_{k=1}^{n} N_k \frac{p_k e^{\lambda p_k}}{e^{\lambda p_k}-1}\,, \tag{S17a}$$

$$\frac{\partial \Lambda}{\partial p_k} = N_k \frac{\lambda e^{\lambda p_k}}{e^{\lambda p_k}-1} - \beta\,, \tag{S17b}$$

$$\frac{\partial \Lambda}{\partial \beta} = 1 - \sum_{i=1}^{n} p_i\,. \tag{S17c}$$

The second derivatives become

$$\frac{\partial^2 \Lambda}{\partial \lambda^2} = N\frac{e^{\lambda}}{(e^{\lambda}-1)^2} - \sum_{k=1}^{n} N_k \frac{p_k^2 e^{\lambda p_k}}{(e^{\lambda p_k}-1)^2}\,, \tag{S18a}$$

$$\frac{\partial^2 \Lambda}{\partial \lambda \partial p_k} = \frac{\partial^2 \Lambda}{\partial p_k \partial \lambda} = N_k \frac{e^{\lambda p_k}}{e^{\lambda p_k}-1}\Big(1 - \frac{\lambda p_k}{e^{\lambda p_k}-1}\Big)\,, \tag{S18b}$$

$$\frac{\partial^2 \Lambda}{\partial p_k^2} = \frac{-N_k \lambda^2 e^{\lambda p_k}}{(e^{\lambda p_k}-1)^2}\,, \tag{S18c}$$

$$\frac{\partial^2 \Lambda}{\partial p_k \partial p_j} = 0 \quad \text{for } k \neq j, \tag{S18d}$$

$$\frac{\partial^2 \Lambda}{\partial p_k \partial \beta} = \frac{\partial^2 \Lambda}{\partial \beta \partial p_k} = -1 \quad \text{for } k \neq j, \tag{S18e}$$

$$\frac{\partial^2 \Lambda}{\partial \beta \partial \lambda} = \frac{\partial^2 \Lambda}{\partial \lambda \partial \beta} = 0\,, \tag{S18f}$$

$$\frac{\partial^2 \Lambda}{\partial \beta^2} = 0\,. \tag{S18g}$$

Application of Lemma 1 yields the following entries of the information matrix:

$$-\mathbb{E}\left(\frac{\partial^2 \Lambda}{\partial \lambda^2}\right) = -\frac{Ne^{\lambda}}{e^{\lambda}-1}\Big(\frac{1}{e^{\lambda}-1} - \sum_{k=1}^{n} \frac{p_k^2}{e^{\lambda p_k}-1}\Big)\,, \tag{S19a}$$

$$-\mathbb{E}\left(\frac{\partial^2 \Lambda}{\partial \lambda \partial p_k}\right) = -\mathbb{E}\left(\frac{\partial^2 \Lambda}{\partial p_k \partial \lambda}\right) = -\frac{Ne^{\lambda}}{e^{\lambda}-1}\Big(1 - \frac{\lambda p_k}{e^{\lambda p_k}-1}\Big)\,, \tag{S19b}$$

$$-\mathbb{E}\left(\frac{\partial^2 \Lambda}{\partial p_k^2}\right) = \frac{Ne^{\lambda}}{e^{\lambda}-1}\,\frac{\lambda^2}{e^{\lambda p_k}-1}\,, \tag{S19c}$$

$$-\mathbb{E}\left(\frac{\partial^2 \Lambda}{\partial p_k \partial p_j}\right) = 0 \quad \text{for } k \neq j, \tag{S19d}$$

$$-\mathbb{E}\left(\frac{\partial^2 \Lambda}{\partial p_k \partial \beta}\right) = -\mathbb{E}\left(\frac{\partial^2 \Lambda}{\partial \beta \partial p_k}\right) = 1 \quad \text{for } k \neq j, \tag{S19e}$$

$$-\mathbb{E}\left(\frac{\partial^2 \Lambda}{\partial \beta \partial \lambda}\right) = -\mathbb{E}\left(\frac{\partial^2 \Lambda}{\partial \lambda \partial \beta}\right) = 0\,, \tag{S19f}$$

$$-\mathbb{E}\left(\frac{\partial^2 \Lambda}{\partial \beta^2}\right) = 0\,. \tag{S19g}$$

Hence, the Fisher information has the following structure

$$\tilde{I}_N = \begin{pmatrix} D & B^T \\ B & A \end{pmatrix} \tag{S20a}$$

with

$$A = \begin{pmatrix} a & 0 \\ 0 & 0 \end{pmatrix}, \quad B = \begin{pmatrix} b_1 & \dots & b_n \\ 1 & \dots & 1 \end{pmatrix} \quad \text{and} \quad D = \operatorname{diag}(d_1, \dots, d_n), \tag{S20b}$$

where

$$a = -\,\mathbb{E}\left(\frac{\partial^2 \Lambda}{\partial \lambda^2}\right), \quad b_k = -\,\mathbb{E}\left(\frac{\partial^2 \Lambda}{\partial \lambda \partial p_k}\right) \quad \text{and} \quad d_k = -\,\mathbb{E}\left(\frac{\partial^2 \Lambda}{\partial p_k^2}\right). \tag{S20c}$$

The inverse Fisher information can be derived using a blockwise inversion formula, namely

$$\tilde{I}_N^{-1} = \begin{pmatrix} D^{-1} + D^{-1}B^T(A - BD^{-1}B^T)^{-1}BD^{-1} & -D^{-1}B^T(A - BD^{-1}B^T)^{-1} \\ -(A - BD^{-1}B^T)^{-1}BD^{-1} & (A - BD^{-1}B^T)^{-1} \end{pmatrix}.$$

The formula applies whenever, $d_i \neq 0$ and the 2×2 matrix $A - BD^{-1}B^T$ is invertible. Blockwise inversion is particularly simple here because D is diagonal. This is the reward of introducing the nuisance parameter β. Without β, D would not be diagonal and inverting the Fisher information would be more involved. Note that

$$A - BD^{-1}B^T = \begin{pmatrix} a_{11} & a_{12} \\ a_{21} & a_{22} \end{pmatrix}, \tag{S21}$$

where $a_{11} = a - \sum_{k=1}^{n} \frac{b_k^2}{d_k}$, $a_{12} = a_{21} = -\sum_{k=1}^{n} \frac{b_k}{d_k}$ and $a_{22} = -\sum_{k=1}^{n} \frac{1}{d_k}$. Its inverse is given by

$$(A - BD^{-1}B^T)^{-1} = \frac{1}{a_{11}a_{22} - a_{12}^2} \begin{pmatrix} a_{22} & -a_{12} \\ -a_{12} & a_{11} \end{pmatrix} =: \begin{pmatrix} m_{11} & m_{12} \\ m_{12} & m_{22} \end{pmatrix}.$$

Moreover,

$$(A - BD^{-1}B^T)^{-1}BD^{-1} = \begin{pmatrix} \left(\frac{m_{11}b_1}{d_1} + \frac{m_{12}}{d_1}\right) & \cdots & \left(\frac{m_{11}b_n}{d_n} + \frac{m_{12}}{d_n}\right) \\ \left(\frac{m_{12}b_1}{d_1} + \frac{m_{22}}{d_1}\right) & \cdots & \left(\frac{m_{12}b_n}{d_n} + \frac{m_{22}}{d_n}\right) \end{pmatrix}$$

and

$$D^{-1}B^T(A - BD^{-1}B^T)^{-1}BD^{-1} = \left(\frac{1}{d_i d_j}\Big(m_{11}b_i b_j + m_{12}(b_i + b_j) + m_{22}\Big)\right)_{ij}.$$

Hence, the entries of the inverse Fisher information $\tilde{I}_N^{-1}$ are

$$\begin{aligned}
\left(\tilde{I}_N^{-1}\right)_{ii} &= \frac{1}{d_i} + \frac{a_{22}b_i^2 - 2a_{12}b_i + a_{11}}{(a_{11}a_{22} - a_{12}^2)d_i^2} && \text{for} \quad i = 1, \dots, n, \\
\left(\tilde{I}_N^{-1}\right)_{ij} &= \frac{a_{22}b_i b_j - a_{12}(b_i + b_j) + a_{11}}{(a_{11}a_{22} - a_{12}^2)d_i d_j} && \text{for} \quad i, j = 1, \dots, n \text{ and } i \neq j, \\
\left(\tilde{I}_N^{-1}\right)_{n+1,j} &= \frac{a_{12} - a_{22}b_j}{(a_{11}a_{22} - a_{12}^2)d_j} && \text{for} \quad j = 1, \dots, n, \\
\left(\tilde{I}_N^{-1}\right)_{n+1,n+1} &= \frac{1}{a_{11} - a_{12}^2/a_{22}},
\end{aligned}$$

where we do not need to calculate its last row and column because these correspond to the nuisance parameter β. After some algebraic manipulation we obtain for $i,j=1,\ldots,n$ and $i\neq j$:

$$\left(\tilde{I}_N^{-1}\right)_{ii} = \frac{(e^\lambda-1)^2}{\lambda^2 N e^\lambda}\left(\frac{e^{\lambda p_i}-1}{e^\lambda-1} + \frac{p_i^2 C - 2p_i(e^{\lambda p_i}-1) + \frac{(e^{\lambda p_i}-1)^2}{e^\lambda-1}}{e^\lambda-1-C}\right), \tag{S22a}$$

$$\left(\tilde{I}_N^{-1}\right)_{ij} = \frac{(e^\lambda-1)^2}{\lambda^2 N e^\lambda}\;\frac{p_i p_j C - p_i(e^{\lambda p_j}-1) - p_j(e^{\lambda p_i}-1) + \frac{(e^{\lambda p_i}-1)(e^{\lambda p_j}-1)}{e^\lambda-1}}{e^\lambda-1-C}, \tag{S22b}$$

$$\left(\tilde{I}_N^{-1}\right)_{n+1,j} = \frac{(e^\lambda-1)^2}{\lambda N e^\lambda}\;\frac{e^{\lambda p_j}-1-p_j C}{e^\lambda-1-C}, \tag{S22c}$$

$$\left(\tilde{I}_N^{-1}\right)_{n+1,n+1} = \frac{(e^\lambda-1)^2}{N e^\lambda}\;\frac{C}{e^\lambda-1-C}, \tag{S22d}$$

where

$$C = \sum_{k=1}^{n}(e^{\lambda p_k}-1). \tag{S22e}$$

Since we do not need the last row and column of the inverse Fisher information, let W denote the matrix obtained by deleting the last row and column of $\left(\tilde{I}_N^{-1}\right)$. (Note that this is different from first deleting the last row and column of the information matrix and then inverting it.) The Cramér-Rao bound V presented in Result 3 is obtained by switching rows and columns in V.

Let $T(\boldsymbol{X}) = (T_{p_1}(\boldsymbol{X}),\ldots,T_{p_n}(\boldsymbol{X}),T_\lambda(\boldsymbol{X}))$ be an estimator for $(\boldsymbol{p},\lambda)$ with expectation $\mathbb{E}(T(\boldsymbol{X})) = \phi(\boldsymbol{X})$. Then, $\mathrm{Cov}(T(\boldsymbol{X})) \geq \frac{\partial\phi}{\partial(\boldsymbol{p},\lambda)} W \left(\frac{\partial\phi}{\partial(\boldsymbol{p},\lambda)}\right)^T$. (Note, that one can still incorporate the nuisance parameter β and use $\tilde{I}_N^{-1}$ by estimating $T_\beta(\boldsymbol{X})=0$.)

E Variance of average MOI

MOI is the average number of super-infections, which – assuming the conditional Poisson distribution – equals

$$\psi = f(\lambda) = \frac{\lambda}{1-e^{-\lambda}}\,, \tag{S23}$$

a 1-1 map between λ and average MOI. Since $\Lambda(\lambda,\beta,\boldsymbol{p}) = \Lambda(f^{-1}(\psi),\beta,\boldsymbol{p})$, the chain rule implies

$$\frac{\partial\Lambda}{\partial\psi} = \frac{\partial\Lambda}{\partial\lambda}\frac{\partial f^{-1}}{\partial\psi} \tag{S24}$$

and the derivative with respect to the other parameters do not change. Further application of the chain rule yields

$$\frac{\partial^2 \Lambda}{\partial \psi^2} = \frac{\partial^2 \Lambda}{\partial \lambda^2}\left(\frac{\partial f^{-1}}{\partial \psi}\right)^2 + \frac{\partial \Lambda}{\partial \lambda}\frac{\partial^2 f^{-1}}{\partial \psi^2}\,, \tag{S25a}$$

$$\frac{\partial^2 \Lambda}{\partial p_k \partial \psi} = \frac{\partial^2 \Lambda}{\partial \psi \partial p_k} = \frac{\partial^2 \Lambda}{\partial p_k \partial \lambda}\frac{\partial f^{-1}}{\partial \psi} + \frac{\partial \Lambda}{\partial \lambda}\frac{\partial^2 f^{-1}}{\partial p_k \partial \psi} = \frac{\partial^2 \Lambda}{\partial p_k \partial \lambda}\frac{\partial f^{-1}}{\partial \psi}\,, \tag{S25b}$$

$$\frac{\partial^2 \Lambda}{\partial \beta \partial \psi} = \frac{\partial^2 \Lambda}{\partial \psi \partial \beta} = \frac{\partial^2 \Lambda}{\partial \beta \partial \lambda}\frac{\partial f^{-1}}{\partial \psi} + \frac{\partial \Lambda}{\partial \lambda}\frac{\partial^2 f^{-1}}{\partial \beta \partial \psi} = \frac{\partial^2 \Lambda}{\partial \beta \partial \lambda}\frac{\partial f^{-1}}{\partial \psi}\,, \tag{S25c}$$

while the remaining second derivatives remain unchanged as in (S18). Note that the score function has mean zero and particularly satisfies $\mathbb{E}\,\frac{\partial \Lambda}{\partial \lambda} = 0$. (That the score function has mean zero can be seen directly from (S17) and Lemma 1. Here it is important to keep in mind that $\boldsymbol{p}$ is an element of the simplex and $\beta = N\frac{\lambda e^{\lambda}}{e^{\lambda}-1}$ for the true parameter and the MLE.) This fact and the inverse function theorem yield

$$-\,\mathbb{E}\,\frac{\partial^2 \Lambda}{\partial \psi^2} = -\left(\frac{\partial f^{-1}}{\partial \psi}\right)^2 \mathbb{E}\,\frac{\partial^2 \Lambda}{\partial \lambda^2} = -\left(\frac{\partial f}{\partial \lambda}\right)^{-2} \mathbb{E}\,\frac{\partial^2 \Lambda}{\partial \lambda^2}\,, \tag{S26a}$$

$$-\,\mathbb{E}\,\frac{\partial^2 \Lambda}{\partial p_k \partial \psi} = -\,\mathbb{E}\,\frac{\partial^2 \Lambda}{\partial \psi \partial p_k} = -\frac{\partial f^{-1}}{\partial \psi}\,\mathbb{E}\,\frac{\partial^2 \Lambda}{\partial p_k \partial \lambda} = -\left(\frac{\partial f}{\partial \lambda}\right)^{-1} \mathbb{E}\,\frac{\partial^2 \Lambda}{\partial p_k \partial \lambda}\,, \tag{S26b}$$

$$-\,\mathbb{E}\,\frac{\partial^2 \Lambda}{\partial \beta \partial \psi} = -\,\mathbb{E}\,\frac{\partial^2 \Lambda}{\partial \psi \partial \beta} = -\frac{\partial f^{-1}}{\partial \psi}\,\mathbb{E}\,\frac{\partial^2 \Lambda}{\partial \beta \partial \lambda} = -\left(\frac{\partial f}{\partial \lambda}\right)^{-1} \mathbb{E}\,\frac{\partial^2 \Lambda}{\partial \beta \partial \lambda}\,. \tag{S26c}$$

The Fisher information for the parameters $(\boldsymbol{p}, \psi, \beta)$ is denoted by $\tilde{J}_N$, and given by

$$\tilde{J}_N = \operatorname{diag}(1, \ldots, 1, \left(\frac{\partial f}{\partial \lambda}\right)^{-1}, 1)\tilde{I}_N \operatorname{diag}(1, \ldots, 1, \left(\frac{\partial f}{\partial \lambda}\right)^{-1}, 1). \tag{S27}$$

Hence,

$$\tilde{J}_N^{-1} = \operatorname{diag}(1, \ldots, 1, \frac{\partial f}{\partial \lambda}, 1)\tilde{I}_N^{-1} \operatorname{diag}(1, \ldots, 1, \frac{\partial f}{\partial \lambda}, 1). \tag{S28}$$

Clearly, $f'(\lambda) = \frac{\partial f}{\partial \lambda} = e^{\lambda}\frac{e^{\lambda}-\lambda-1}{(e^{\lambda}-1)^2}$, so that for $i, j = 1, \ldots, n$ and $i \neq j$:

$$(\tilde{J}_N^{-1})_{ii} = (\tilde{I}_N^{-1})_{ii}\,, \quad (\tilde{J}_N^{-1})_{ij} = (\tilde{I}_N^{-1})_{ij}\,, \tag{S29a}$$

$$(\tilde{J}_N^{-1})_{n+1,j} = f'(\lambda)(\tilde{I}_N^{-1})_{n+1,j} = \frac{e^{\lambda}-\lambda-1}{\lambda N}\;\frac{e^{\lambda p_j}-1-p_j C}{e^{\lambda}-1-C}\,, \tag{S29b}$$

$$(\tilde{J}_N^{-1})_{n+1,n+1} = f'(\lambda)^2(\tilde{I}_N^{-1})_{n+1,n+1} = \frac{e^{\lambda}(e^{\lambda}-\lambda-1)^2}{N(e^{\lambda}-1)^2}\;\frac{C}{e^{\lambda}-1-C}\,, \tag{S29c}$$

where C is given by (S22e). The last row and column of $\tilde{J}_N^{-1}$ are irrelevant as they correspond to the nuisance parameter. By deleting the last row and column and rearranging the remaining rows and columns, the Cramér-Rao bound of Remark 5 is obtained.

F Constructing data

F.1 Dependence on true parameters and sample size

To investigate the method's sensitivity on the true parameters we proceeded as follows. For a fixed set of parameters $\boldsymbol{\theta} = (\lambda, \boldsymbol{p})$ we simulated K data sets $\boldsymbol{X}^{(1)}, \ldots, \boldsymbol{X}^{(K)}$ of

sample size N as described below. For each data set $\boldsymbol{X}^{(k)}$, we derived the MLE $\hat{\boldsymbol{\theta}}^{(k)}$. (Only regular data sets, i.e. those satisfying $N_k \neq N$ for all k and $\sum_{k=1}^{n} N_k > N$ were constructed, so the MLE was well defined.) We then attained the MLE's performance as described below. This was repeated for several parameter choices $\boldsymbol{\theta}$. Particularly, the parameters' dimension, n, might also affect the quality of the MLE. Therefore, parameters of different dimension were carefully chosen.

F.2 Dependence on sample size and dimension

To investigate the method's dependency on sample size we repeated the procedure described above for several choices of sample size N. This provides not only a proxy for the method's overall performance under varying sample size, but also allows identification of the necessary sample size for different parameter regions to achieve certain performance goals.

F.3 Model fit

The performance of the methods are justified only if the true model that generates the data equals the model used to derive the likelihood function. To investigate a potential dependence on model violations, we generated the data sets $\boldsymbol{X}^{(k)}$ under alternative models, and repeated the procedures described above for each of them. This gives a proxy for the method's sensitivity to model violations. Particularly, we assumed that the number of (super-)infections follows a conditional Poisson (correct model), shifted Poisson, conditional and shifted binomial, and uniform distribution as described below.

F.4 Constructing the data

We first describe how a data set $\boldsymbol{X}$ is constructed under the conditional Poisson model, which is the model underlying the MLE. We then describe adaptations to construct data under different models.

Conditional Poisson model. The number Y of parasites infecting a host follows a conditional Poisson distribution, i.e.,

$$P(Y = m) = \frac{1}{e^{\lambda} - 1} \frac{\lambda^m}{m!} \quad \text{for } m \geq 1. \tag{S30}$$

MOI is the average number of infecting lineages and is given by

$$\psi = \psi_{CP} = \mathbb{E}\,Y = \frac{\lambda}{1 - e^{-\lambda}}. \tag{S31}$$

Moreover, conditional on being infected by m parasites, the infecting lineages are drawn from a multinomial distribution with parameters m and $\boldsymbol{p}$, i.e.,

$$P(\boldsymbol{m} = (m_1, \ldots, m_n) | Y = m) = \frac{m!}{m_1! \ldots m_n!} p_1^{m_1} \ldots p_n^{m_n}, \tag{S32}$$

where $m_j \in \{0, \ldots, m\}$ is the number of times the host is infected with lineage j. Clearly, $m_1 + \ldots + m_n = m$. The corresponding blood sample is hence $(\operatorname{sign} m_1, \ldots, \operatorname{sign} m_n)$.

Summarizing, first a random number is generated according to (S30). Then, an integer vector $\boldsymbol{m} = (m_1, \ldots, m_n)$ is generated according to (S32), which yields the first blood sample as $\boldsymbol{x}_1 = (\operatorname{sign} m_1, \ldots, \operatorname{sign} m_n)$. By repeating this procedure N times a dataset $\boldsymbol{X}$ containing N blood samples is obtained.

Shifted Poisson model. The difference to the conditional Poisson model is the distribution of super-infecting lineages. Namely, the number of super-infecting lineages follow a Poisson distribution that is shifted by 1, i.e.,

$$P(Y = m) = \frac{1}{e^{\lambda}} \frac{\lambda^{m-1}}{(m-1)!} \quad \text{for } m \geq 1. \tag{S33}$$

The mean number of super-infections (MOI) is

$$\psi = \psi_{SP} = \mathbb{E}\, Y = \lambda + 1. \tag{S34}$$

Binomial model. Here, the number of super-infecting lineages is conditionally binomially distributed with parameters M and p, i.e.,

$$P(Y = m) = \binom{M}{m} \frac{p^m (1-p)^{M-m}}{1-(1-p)^M} \quad \text{for } m \in \{1, \ldots, M\}. \tag{S35}$$

MOI in this case is

$$\psi = \psi_{CB} = \mathbb{E}\, Y = \frac{Mp}{1-(1-p)^M}. \tag{S36}$$

For large M, small p and $Mp \sim \lambda$ this model approximates the conditional Poisson model.

Shifted binomial model. As an approximation to the shifted Poisson model, the number of super-infecting lineages follows a shifted binomial distribution, namely,

$$P(Y = m) = \binom{M}{m-1} p^{m-1} (1-p)^{M-m+1} \qquad \text{for} \qquad m = 1, \ldots, M+1, \tag{S37}$$

for which MOI is

$$\psi = \psi_{SB} = \mathbb{E}\, Y = Mp + 1. \tag{S38}$$

Uniform model. The assumption here is that a host is infected by 1 to M lineages with the same probability, i.e.,

$$P(Y = m) = \frac{1}{M} \tag{S39}$$

for which MOI is

$$\psi = \psi_{UN} = \mathbb{E}\, Y = \frac{M+1}{2}. \tag{S40}$$

Parameter values. We simulated data sets for all models for a wide range of parameters. Importantly, these were chosen so that the values of the MOI parameters were matching, i.e., $\psi_{CP} = \psi_{SP} = \psi_{CB} = \psi_{SB}$, except for the uniform model, for which ψ_{UN} can attain only a limited number of values. The parameters are summarized in Table A.

F.5 Measuring MLE's performance

The first aim is to measure the performance of the MLE for the Poisson parameter λ, or rather for MOI, $\psi = \frac{\lambda}{1-e^{-\lambda}}$. (This is only the correct parameter for the conditional Poisson model, not for the alternative models used to simulate data.) We refer to a combination of parameters and a given model as a parameter set. For each parameter set $K = 10\,000$ data sets $\boldsymbol{X}^{(k)}$ were randomly generated as described above. For each data set the MLE $\hat{\lambda}^{(k)}$ is derived. We then derived the empirical mean ($\mathbb{E}_K\,\hat{\lambda}$) and variance ($\mathrm{Var}_K\,\hat{\lambda}$) of the $\hat{\lambda}^{(k)}$s, as well as the 0.5%, 2.5%, 5%, 95%, 97.5%, and 99.5% percentiles. These measures are informative about the distribution of bias and variance of the MLE.

To assess quality of the estimate for the lineage frequencies, we obtained the empirical means, variances and 0.5%, 2.5%, 5%, 95%, 97.5%, and 99.5% quantiles for each lineage frequency, i.e., for $p_1, \ldots, p_n$. Although these measures are important, usually the focus does not lie on single lineage frequencies but on the overall distribution, particularly because the parameters are correlated. Moreover, as n increases it becomes increasingly tedious to look at measures for single frequencies, rather than on a single quantity. Therefore, we also calculated the distance $d(\boldsymbol{p}, \hat{\boldsymbol{p}})$ from the true and estimated parameters. For each set of parameters we then obtained the empirical means and variances of the distance, i.e., $\mathbb{E}_K\, d(\boldsymbol{p}, \hat{\boldsymbol{p}})$ and $\mathrm{Var}_K\, d(\boldsymbol{p}, \hat{\boldsymbol{p}})$. Since $\boldsymbol{p}$ is multidimensional, it is difficult to decide on an appropriate distance. Because we do not want to decide on a "best" distance measure, we used several distance measures (Euclidian-norm, 1-norm, supreme norm, Jensen-Shannon divergence, Hellinger and Bhattacharyy distances). Qualitatively they all yielded the same results.

Tables

summary of parameters		
par.	description	values
K	number of data sets per par. combination	10 000 or 100 000
λ	par. of conditional Poisson model	$0.1, \ldots, 2$ in steps of 0.05
ψ_{CP}	MOI parameter	$\frac{\lambda}{1-e^{-\lambda}}$ with λ as above
ψ_{SP}	MOI parameter	$\lambda + 1$ with λ such that $\psi_{SP} = \psi_{CP}$
ψ_{CB}	MOI parameter	$\frac{Mp}{1-(1-p)^M}$ with $M = 8$, p such that $\psi_{CB} = \psi_{CP}$
ψ_{SB}	MOI parameter	$Mp + 1$ with $M = 8$, p such that $\psi_{CB} = \psi_{CP}$
ψ_{UN}	MOI parameter	$\frac{M+1}{2}$ with $M = 2, 3, 4, 5, 7, 8$
N	sample size	40, 50, 60, 70, 80, 90, 100, 150, 200, 250, 300, 350, 400
n	number of lineages	2, 3, 4, 5, 10
$\boldsymbol{p}$	lineage-frequencies for	
	$n = 2$	$(0.5, 0.5)$, $(0.6, 0.4)$, $(0.7, 0.3)$, $(0.8, 0.2)$, $(0.9, 0.1)$
	$n = 3$	$(1/3, 1/3, 1/3)$, $(0.45, 0.45, 0.1)$, $(0.6, 0.3, 0.1)$, $(0.7, 0.2, 0.1)$, $(0.8, 0.15, 0.05)$, $(0.9, 0.05, 0.05)$
	$n = 4$	$(0.25, 0.25, 0.25, 0.25)$, $(0.4, 0.2, 0.2, 0.2)$, $(0.4, 0.4, 0.1, 0.1)$ $(0.5, 0.25, 0.2, 0.05)$, $(0.6, 0.3, 0.05, 0.05)$, $(0.7, 0.15, 0.1, 0.05)$
	$n = 5$	$(0.2, 0.2, 0.2, 0.2, 0.2)$, $(0.25, 0.2, 0.2, 0.2, 0.15)$, $(0.3, 0.2, 0.2, 0.15, 0.15)$, $(0.4, 0.2, 0.2, 0.1, 0.1)$, $(0.5, 0.2, 0.2, 0.05, 0.05)$, $(0.6, 0.2, 0.1, 0.05, 0.05)$, $(0.7, 0.1, 0.1, 0.05, 0.05)$, $(0.8, 0.05, 0.05, 0.05, 0.05)$
	$n = 10$	$(0.1, 0.1, 0.1, 0.1, 0.1, 0.1, 0.1, 0.1, 0.1, 0.1)$, $(0.4, 0.4, 0.09, 0.05, 0.01, 0.01, 0.01, 0.01, 0.01, 0.01)$, $(0.7, 0.13, 0.1, 0.01, 0.01, 0.01, 0.01, 0.01, 0.01, 0.01)$, $(0.91, 0.01, 0.01, 0.01, 0.01, 0.01, 0.01, 0.01, 0.01, 0.01)$

Table A. Summary of parameters. Displayed are the parameter values used to construct the data sets for the simulation study. Parameters were chosen so that the same MOI parameters were obtained for the conditional Poisson, shifted Poisson, conditional binomial, and shifted binomial model. In the case of the uniform model it is impossible to accomplish this. K data sets were created for each resulting combination of values of ψ, $\boldsymbol{p}$, N.

References

1. Schneider KA, Escalante AA. A Likelihood Approach to Estimate the Number of Co-Infections. PLoS ONE. 2014;9(7):e97899. doi:10.1371/journal.pone.0097899.
