# Supplementary material for: Large and finite sample properties of a maximum-likelihood estimator for multiplicity of infection
Source: PLoS One. 2018 Apr 9;13(4):e0194148. doi: 10.1371/journal.pone.0194148 (PMC5890990; doi:10.1371/journal.pone.0194148)
Supplement: S5 File — Additional figures showing detailed results. (PDF) [file pone.0194148.s005.pdf]

Supporting Information
Additional Figures

Kristan Alexander Schneider

Contents

A Bias of $\hat{\lambda}$ **2**

B Median bias of $\hat{\lambda}$ **7**

C Variance of $\hat{\lambda}$ **12**

D Bias of frequency estimates **17**

E Variance of frequency estimates **22**

F Model violations **37**

A Bias of $\hat{\lambda}$

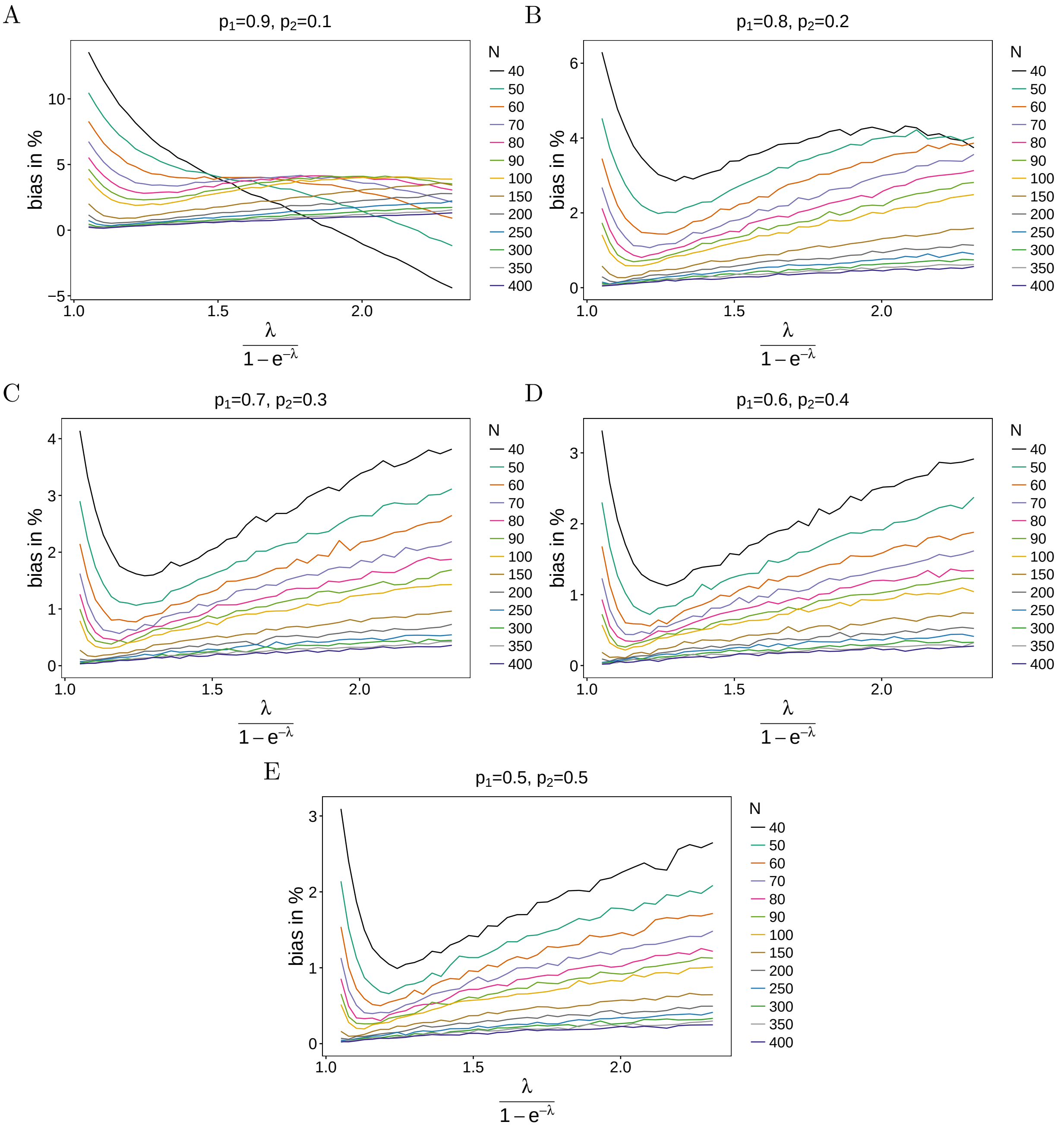

Figure 1: **Bias for the conditional Poisson model.** (A)-(E) Shown is the bias of the MLE $\hat{\psi}$ in percent of the true parameter as a function of the true parameter $\psi = \frac{\lambda}{1-e^{-\lambda}}$ based on simulated data created by the conditional Poisson model. For each parameter combination $K = 100\,000$ data sets were simulated. Each panel assumes different lineage-frequency distributions $\boldsymbol{p}$ shown at the top of each panel. Colored lines correspond to different sample sizes N.

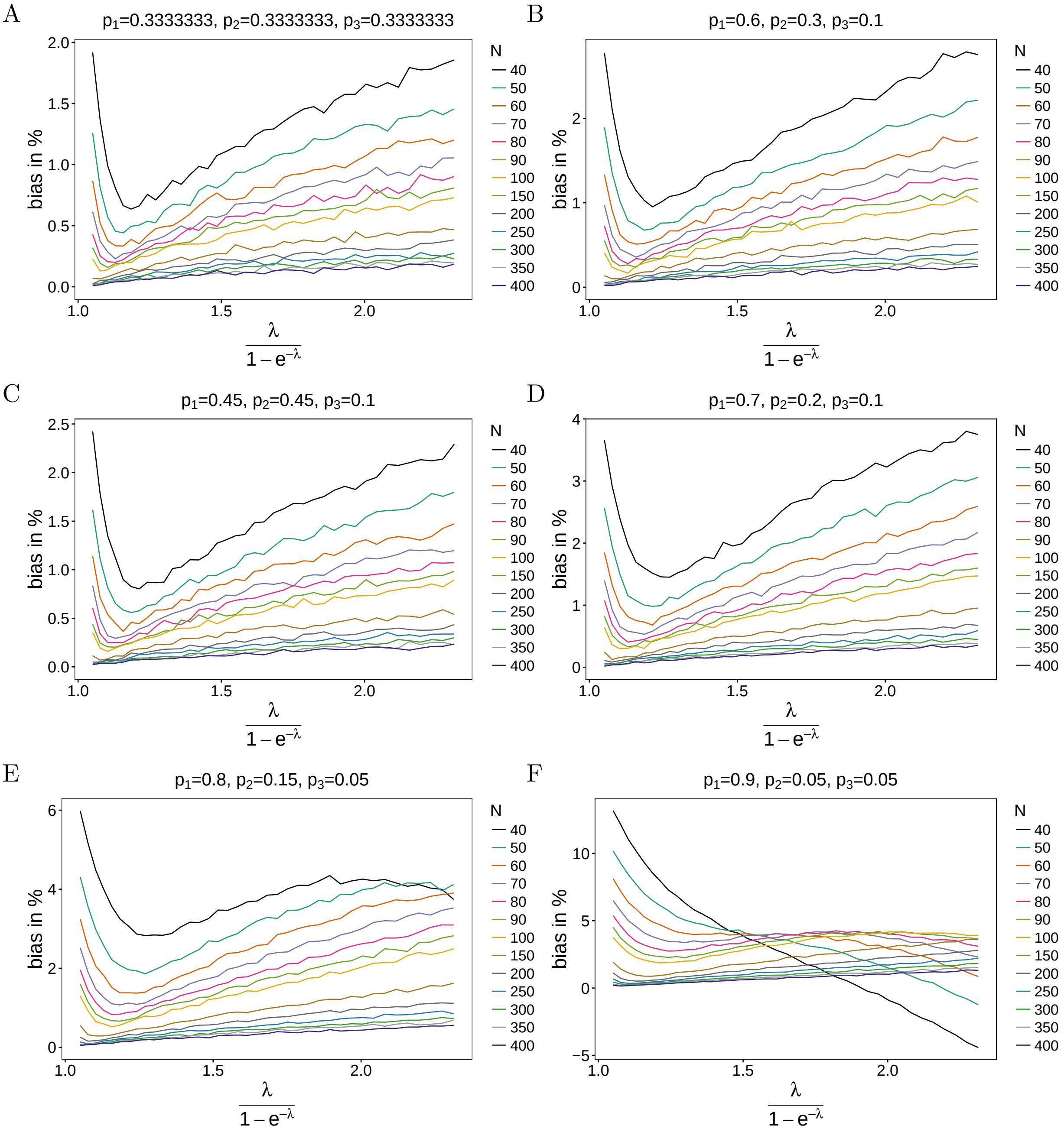

Figure 2: See Fig 1.

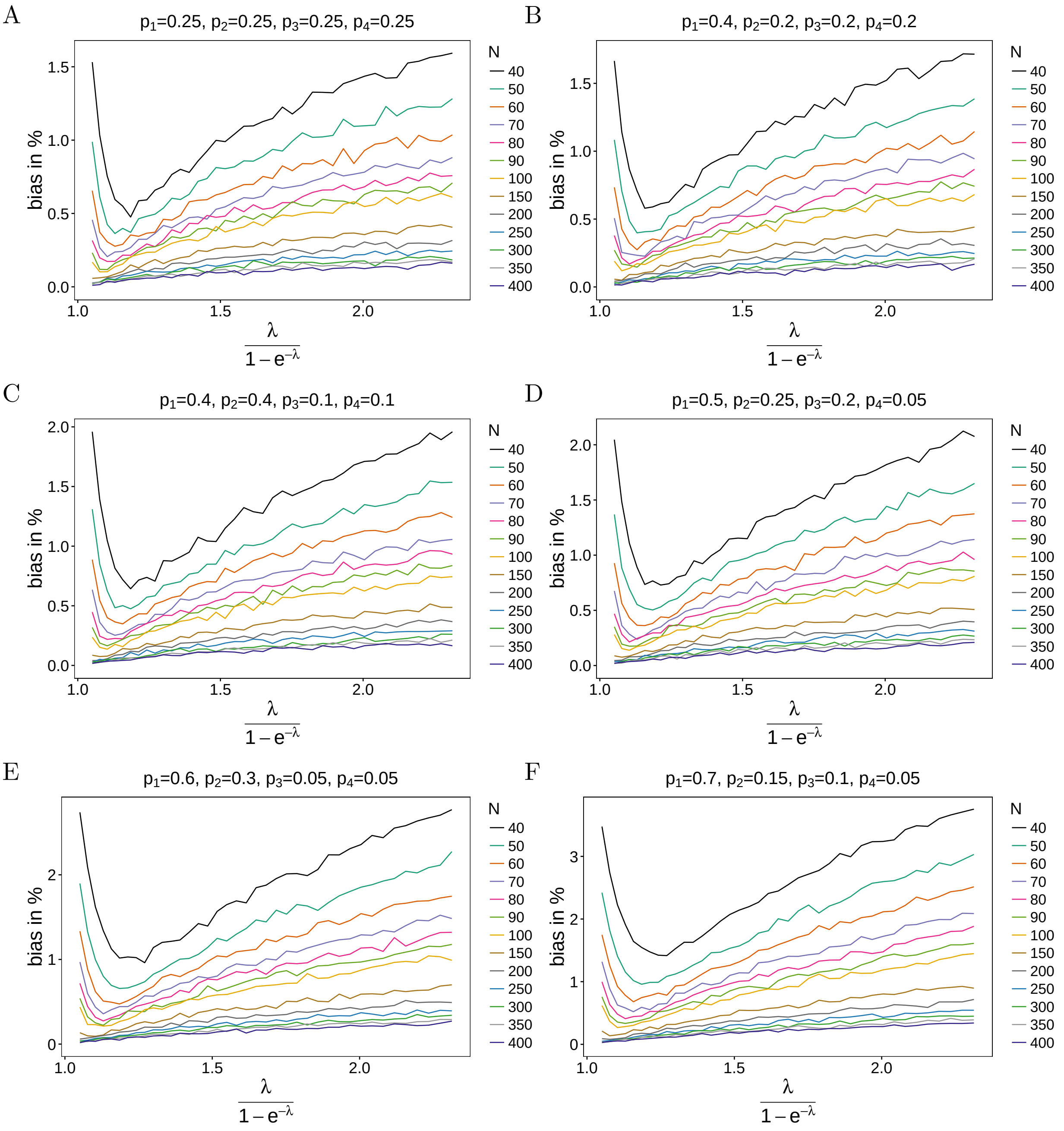

Figure 3: See Fig 1.

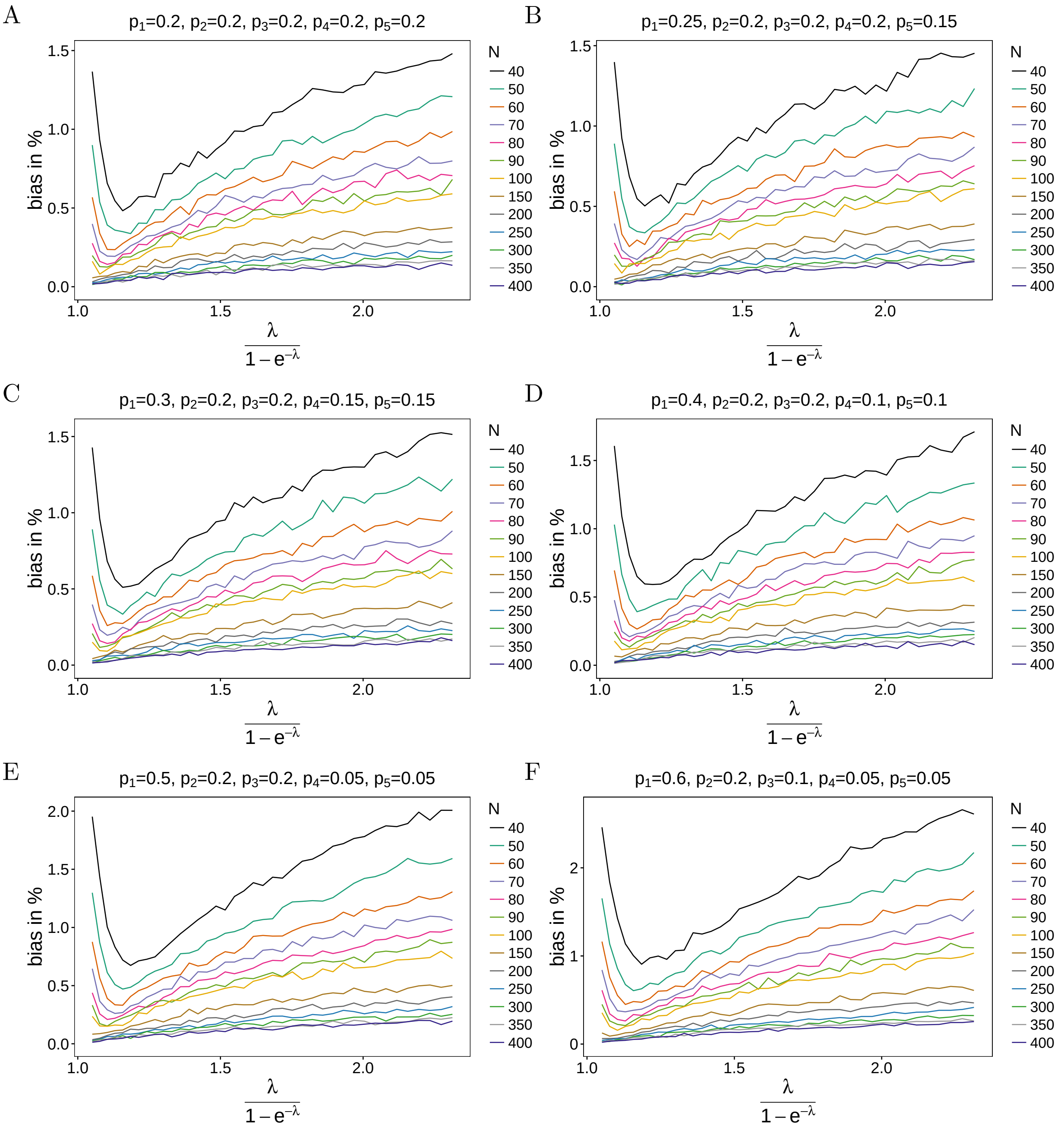

Figure 4: See Fig 1.

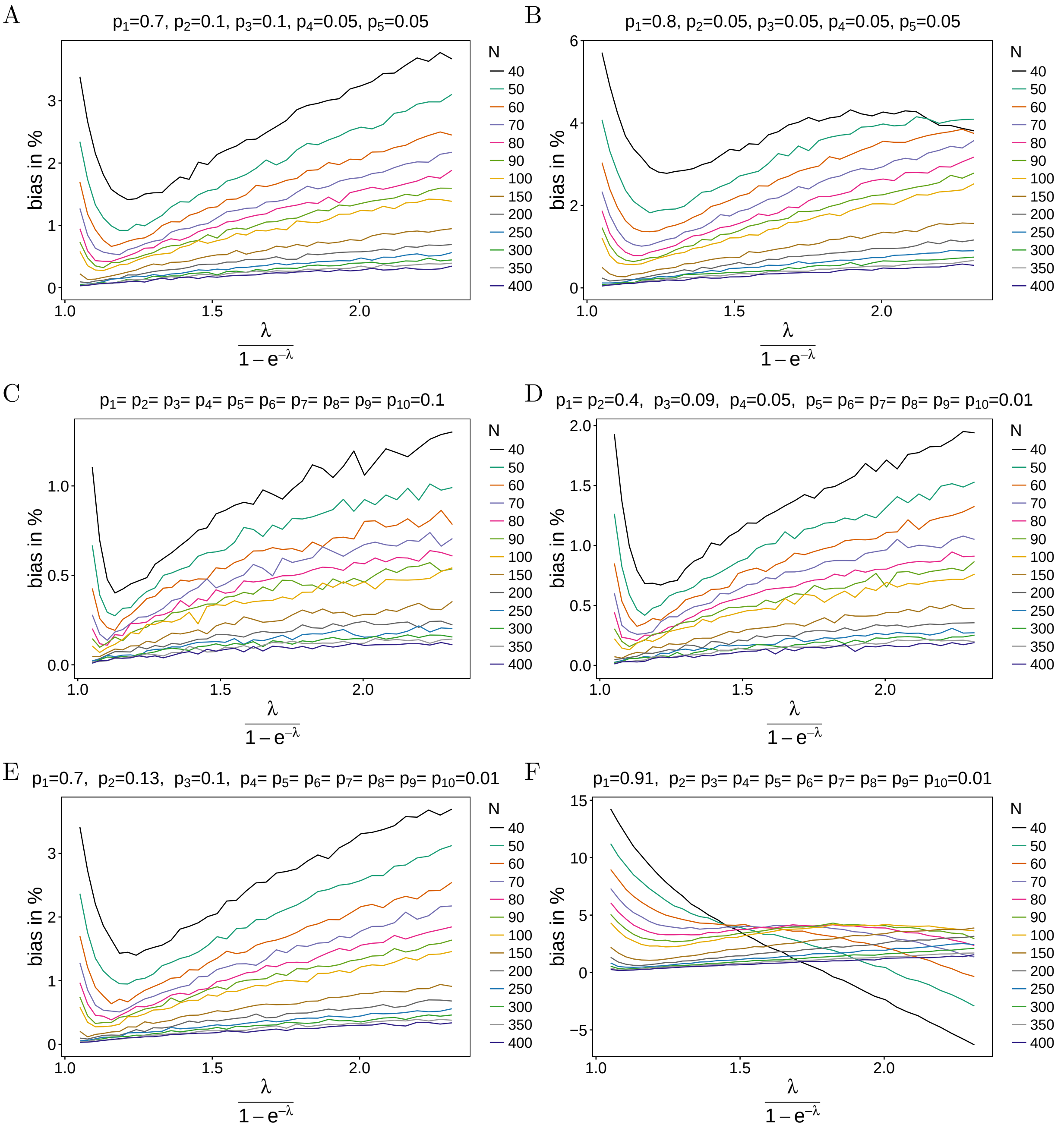

Figure 5: See Fig 1.

B Median bias of $\hat{\lambda}$

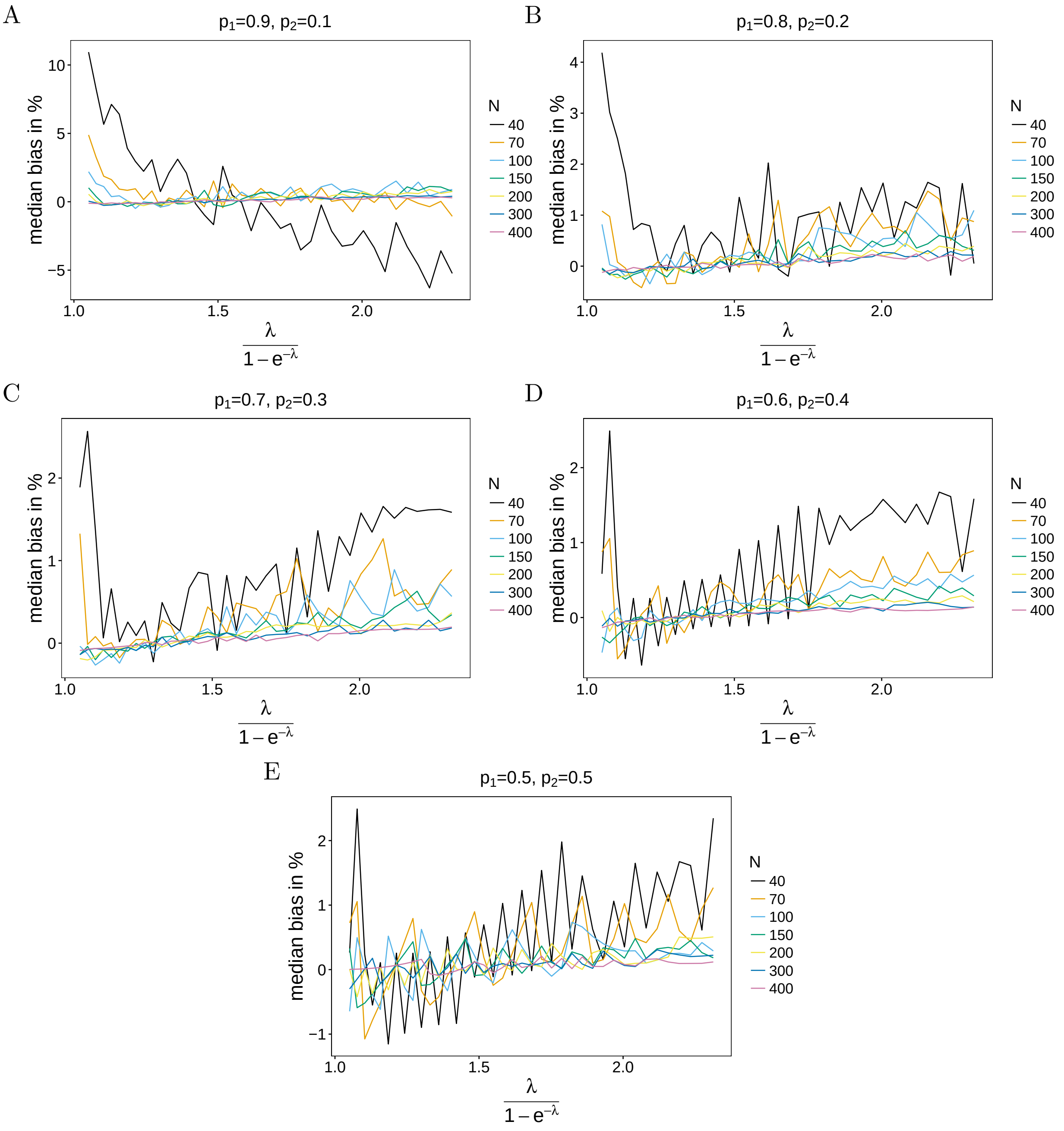

Figure 6: **Median bias for the conditional Poisson model.** (A)-(E) Shown is the media bias of the MLE $\hat{\psi}$ in percent of the true parameter as a function of the true parameter $\psi = \frac{\lambda}{1-e^{-\lambda}}$ based on simulated data created by the conditional Poisson model.For each parameter combination $K = 100\,000$ data sets were simulated. Each panel assumes different lineage-frequency distributions $\boldsymbol{p}$ shown at the top of each panel. Colored lines correspond to different sample sizes N.

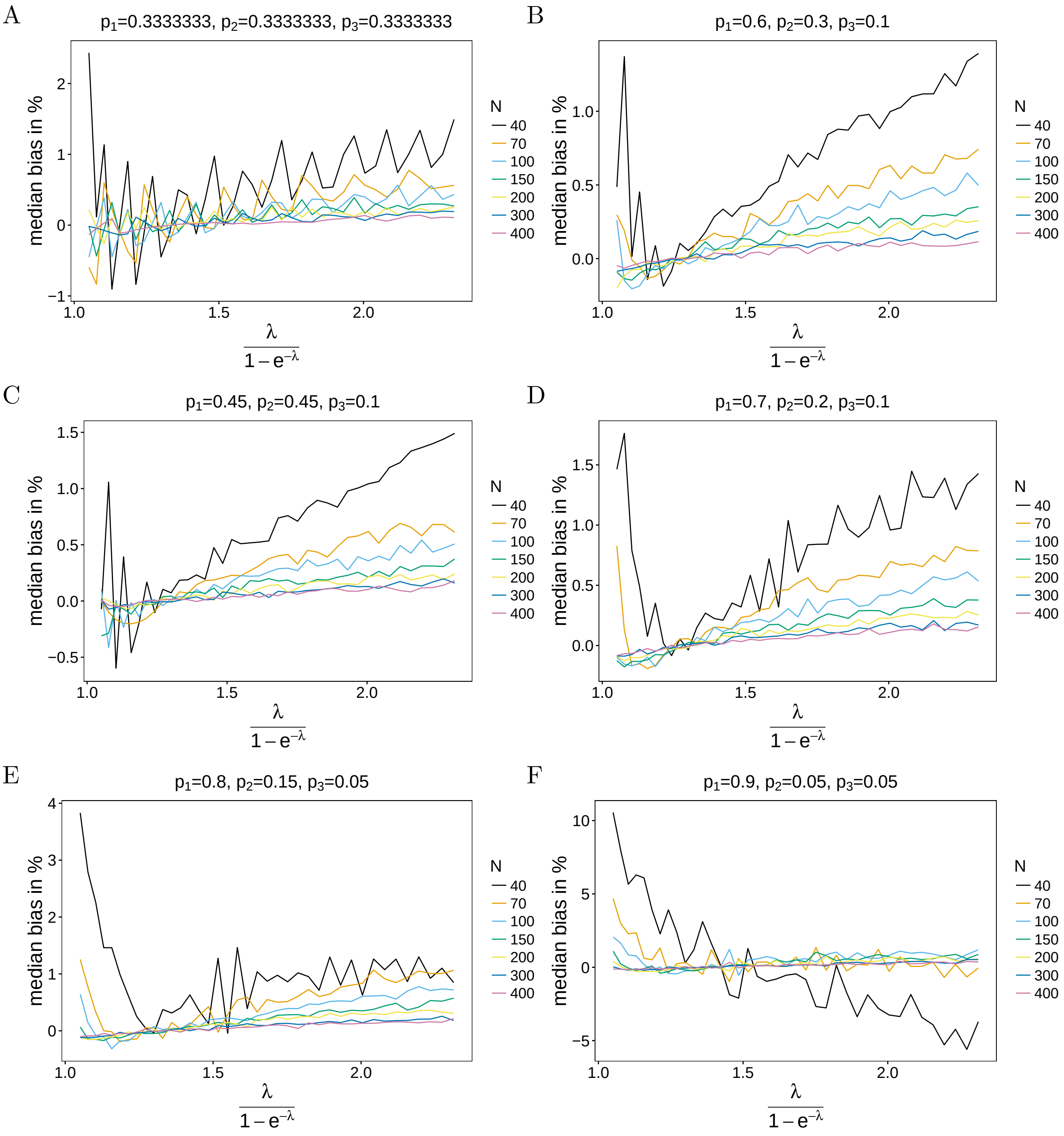

Figure 7: See Fig 6.

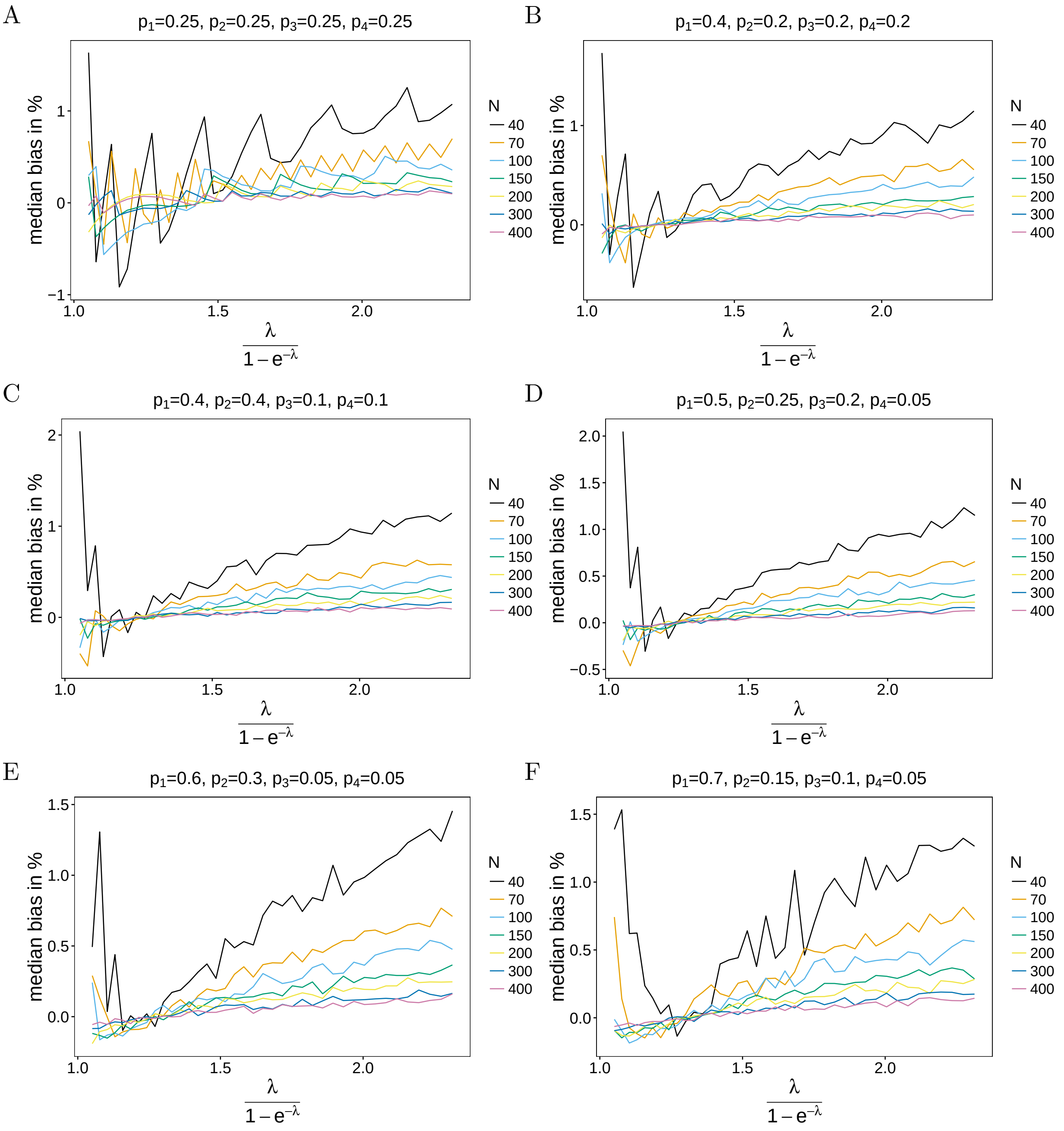

Figure 8: See Fig 6.

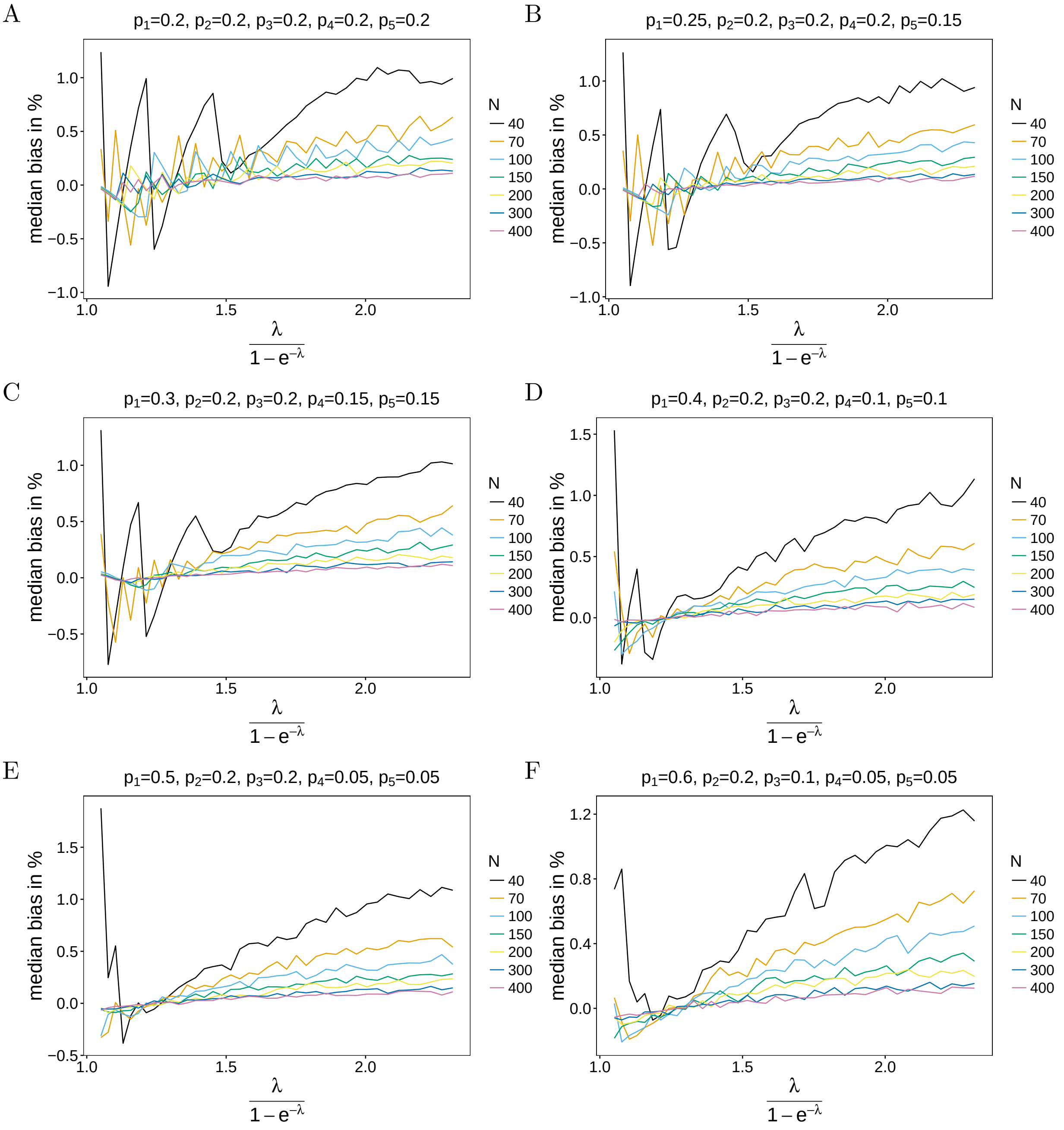

Figure 9: See Fig 6.

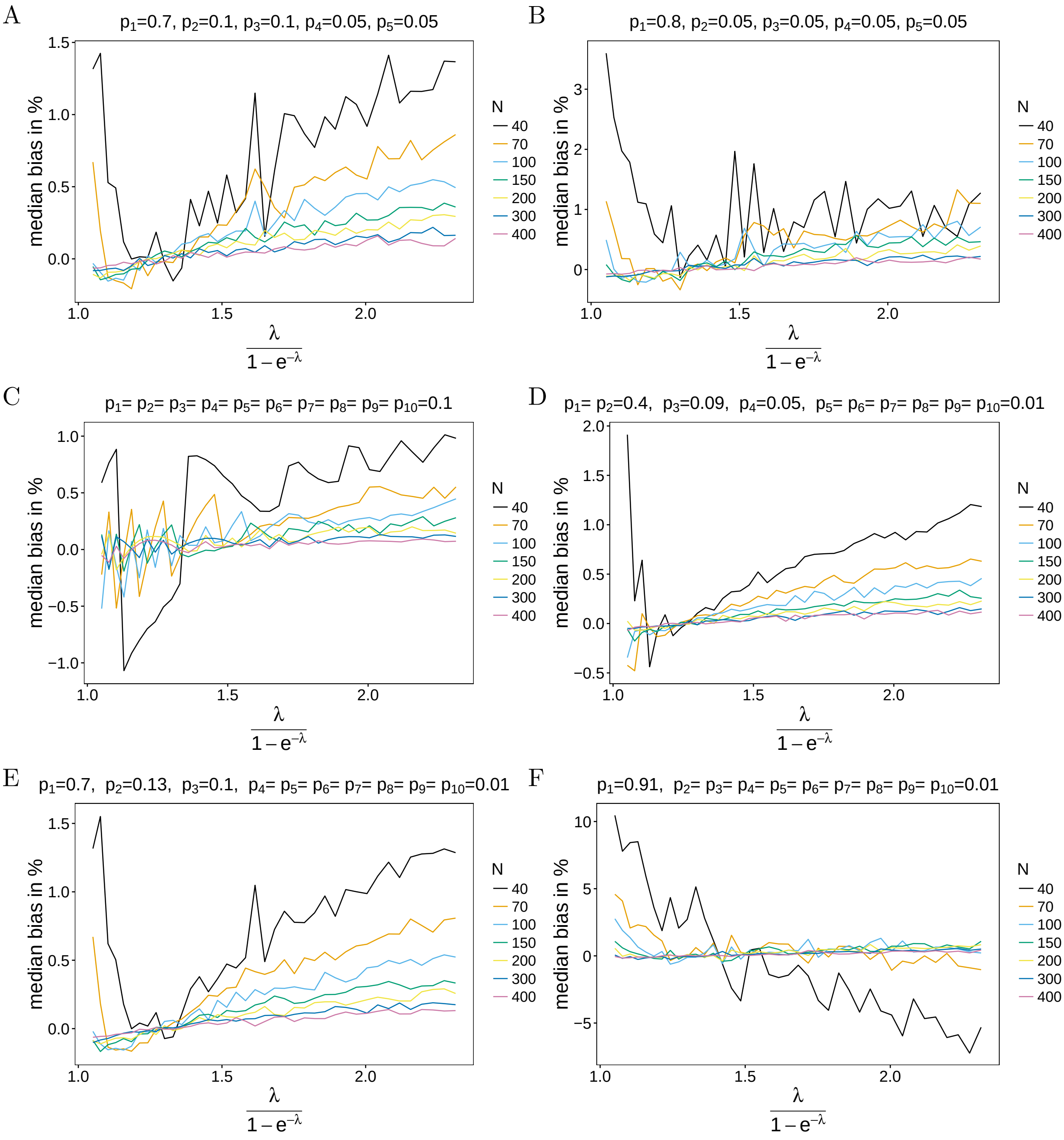

Figure 10: See Fig 6.

C Variance of $\hat{\lambda}$

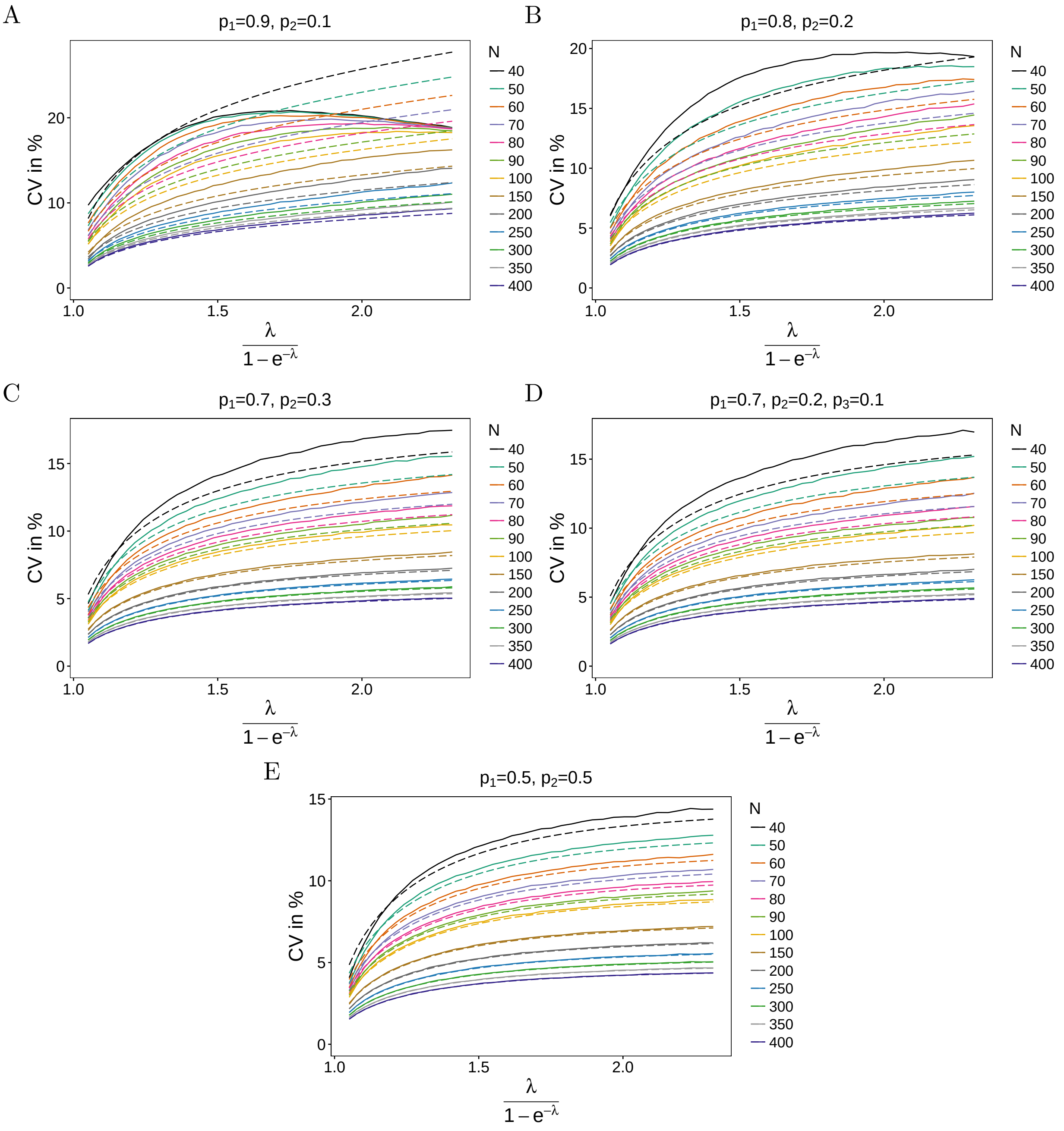

Figure 11: **Coefficient of variance in 100% of $\hat{\psi}$ for the conditional Poisson model.** (A)-(E) Shown is the coefficient of variation in %, i.e., $\times 100$, of the MLE $\hat{\psi}$ based on simulated data created by the conditional Poisson model (solid lines) and its theoretical prediction based on the Cramér-Rao lower bound evaluated at the true parameters (dashed lines). For each parameter combination $K = 100\,000$ data sets were simulated. Each panel assumes different lineage-frequency distributions $\boldsymbol{p}$ shown at the top of each panel. Colored lines correspond to different sample sizes N.

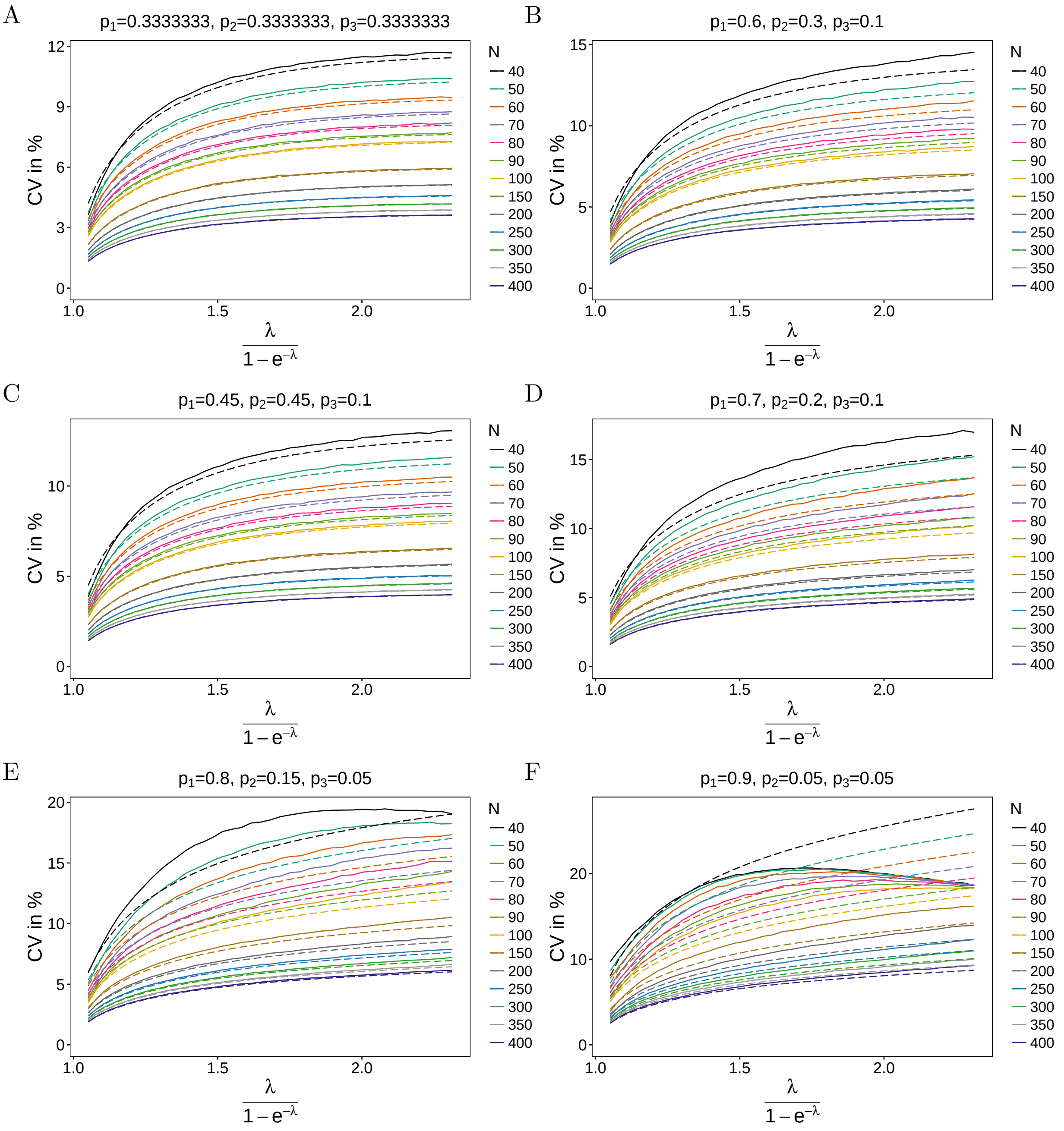

Figure 12: See Fig 11.

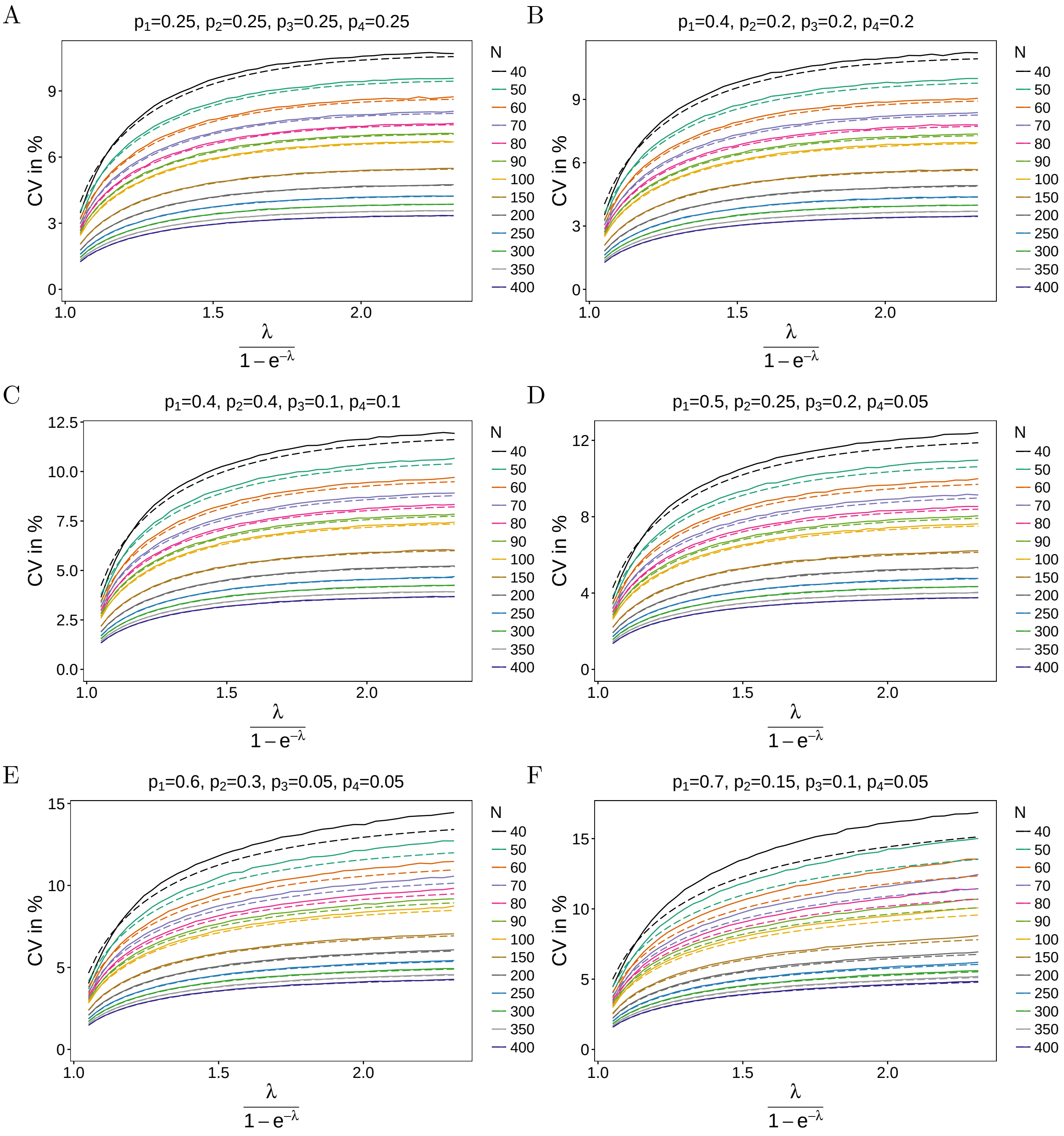

Figure 13: See Fig 11.

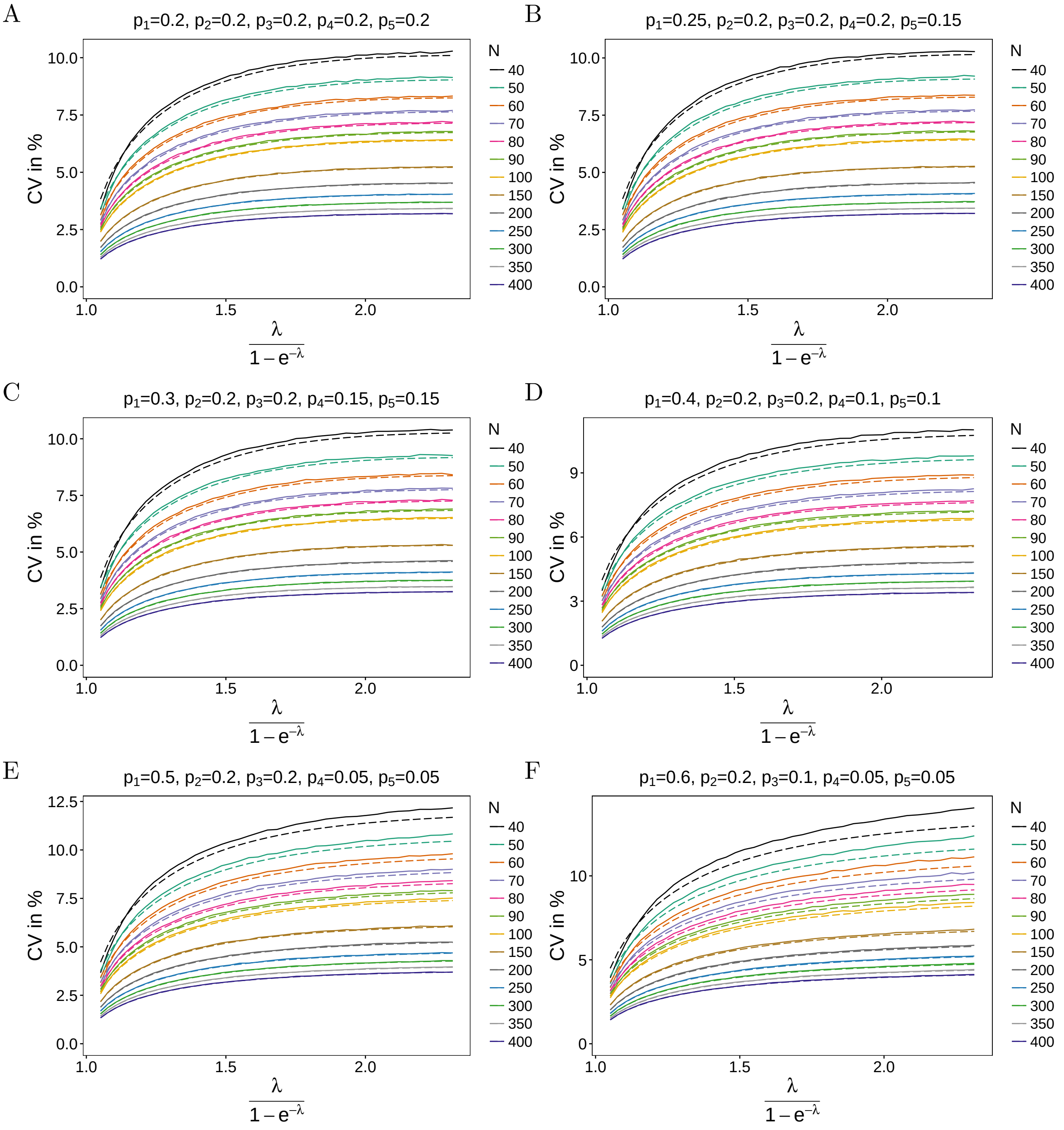

Figure 14: See Fig 11.

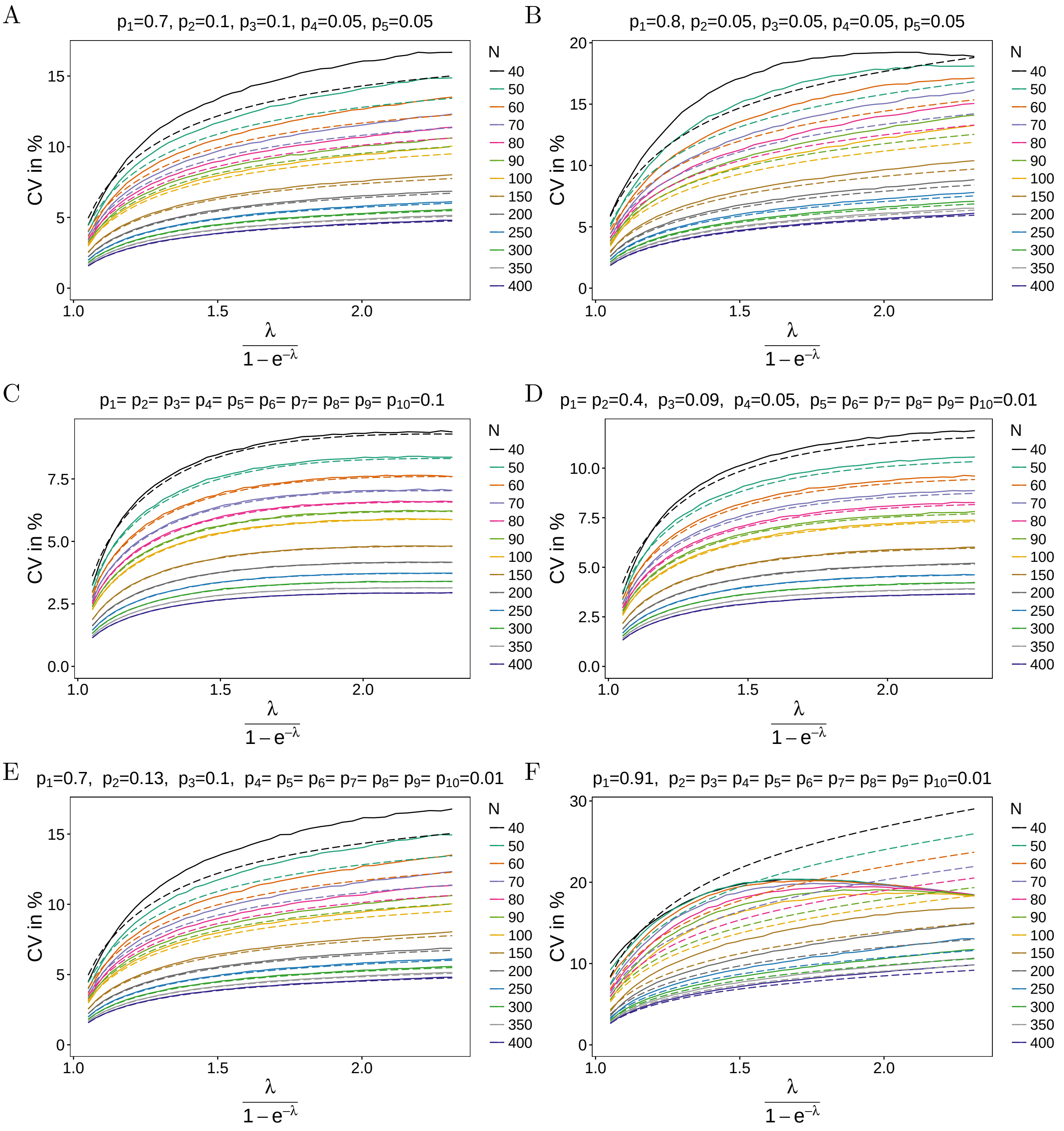

Figure 15: See Fig 11.

D Bias of frequency estimates

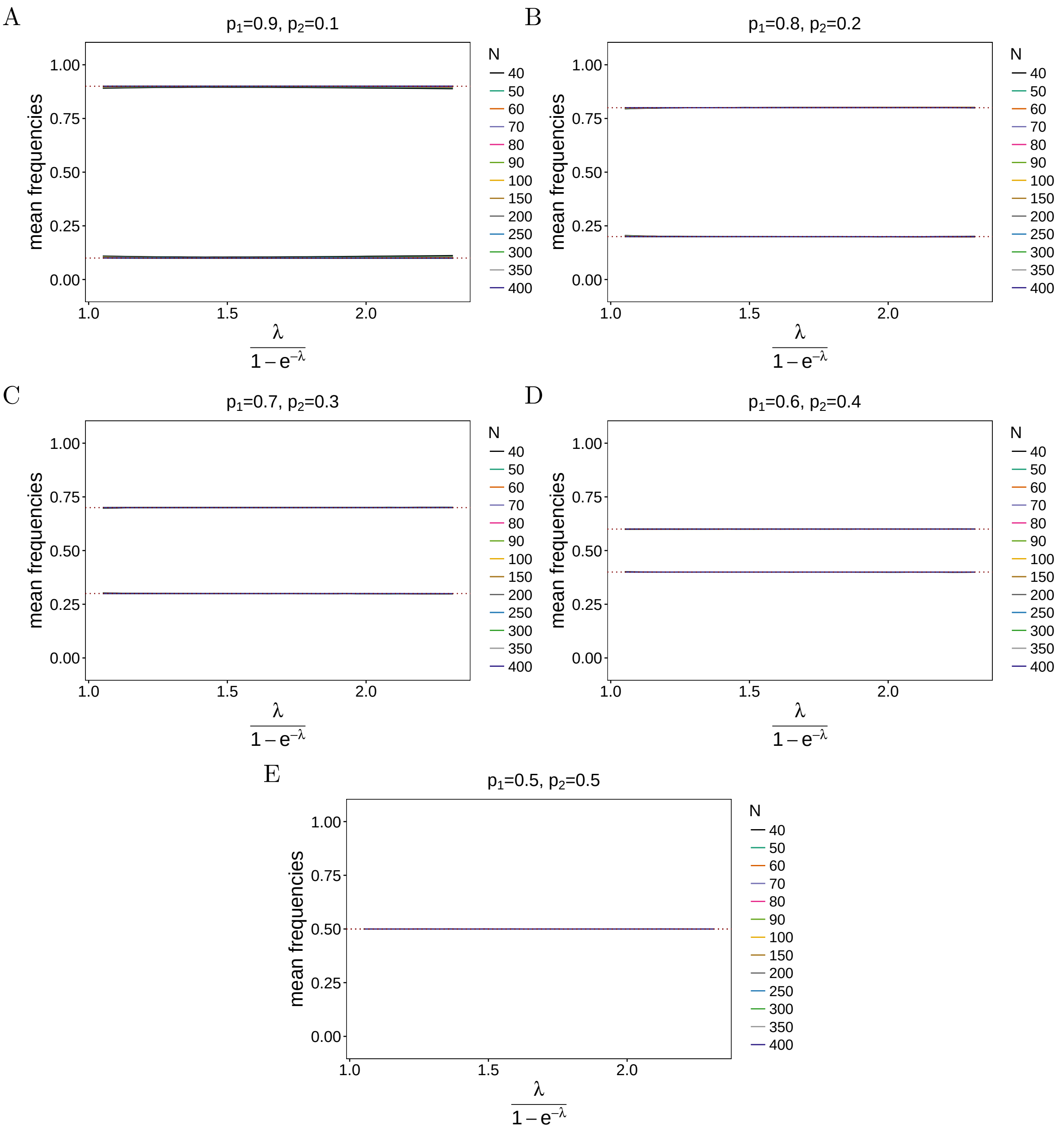

Figure 16: **Bias of $\hat{\boldsymbol{p}}$.** (A)-(E) Shown are the averages of the frequency estimates $\hat{p}_1, \ldots, \hat{p}_n$ of the K simulated data sets created by the conditional Poisson model. For each parameter combination $K = 100\,000$ data sets were simulated. Each panel assumes different lineage-frequency distributions $\boldsymbol{p}$ shown at the top of each panel. Colored lines correspond to different sample sizes N. Red dotted horizontal lines mark the true frequencies $\hat{p}_1, \ldots, \hat{p}_n$. Because the frequency estimates are almost unbiased independently of sample size, the lines for different N stack on top of each other.

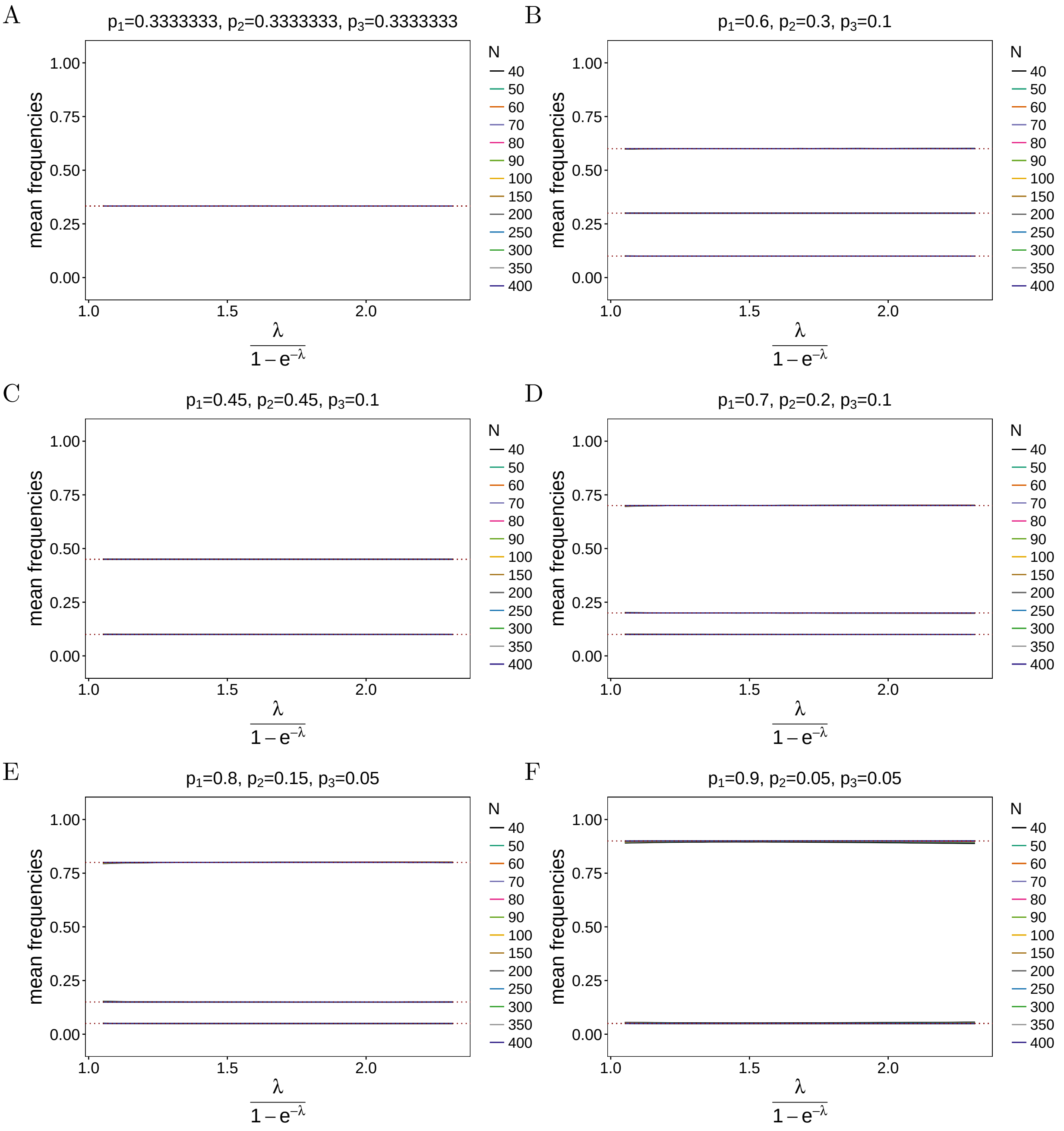

Figure 17: See Fig 16.

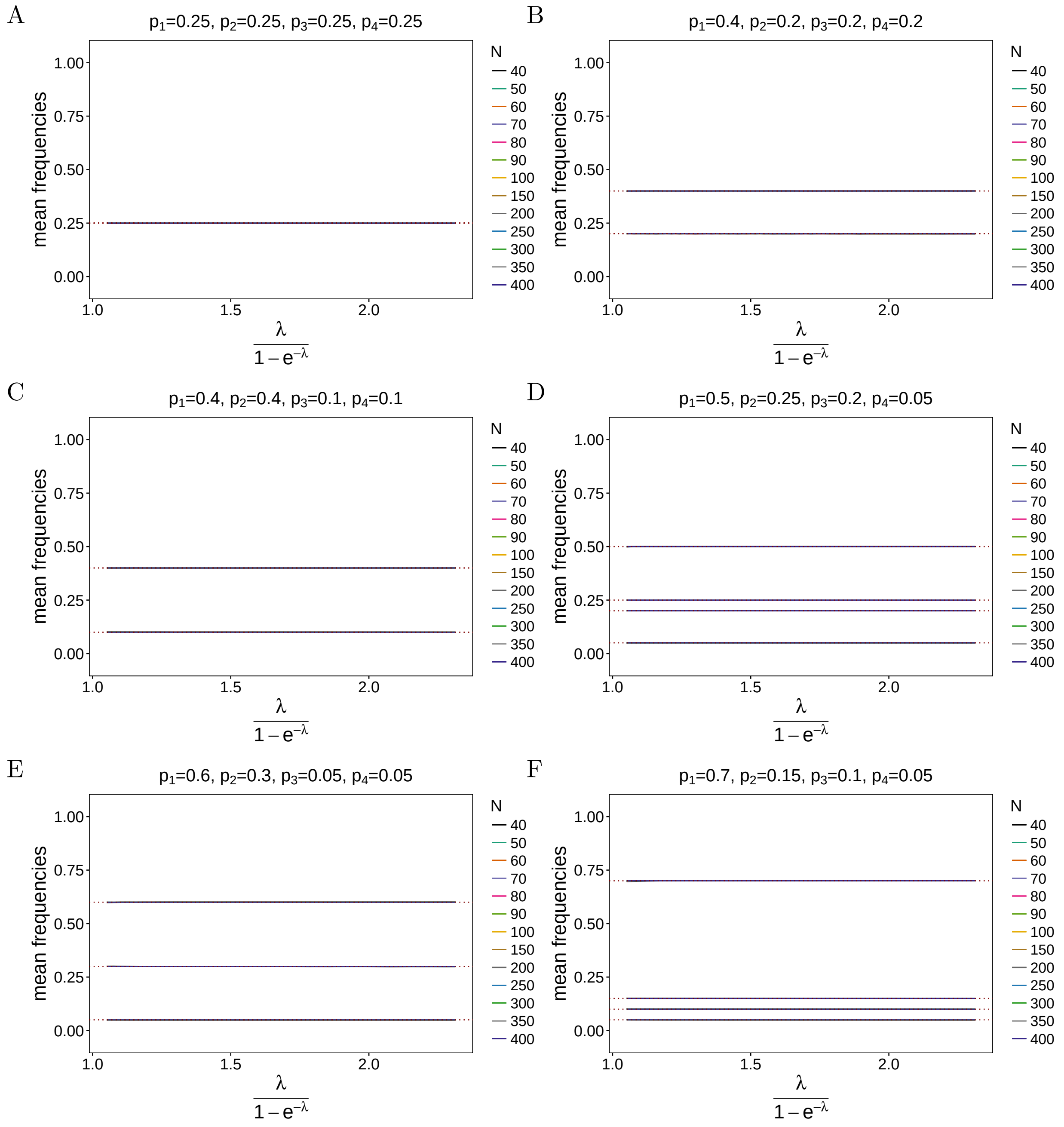

Figure 18: See Fig 16.

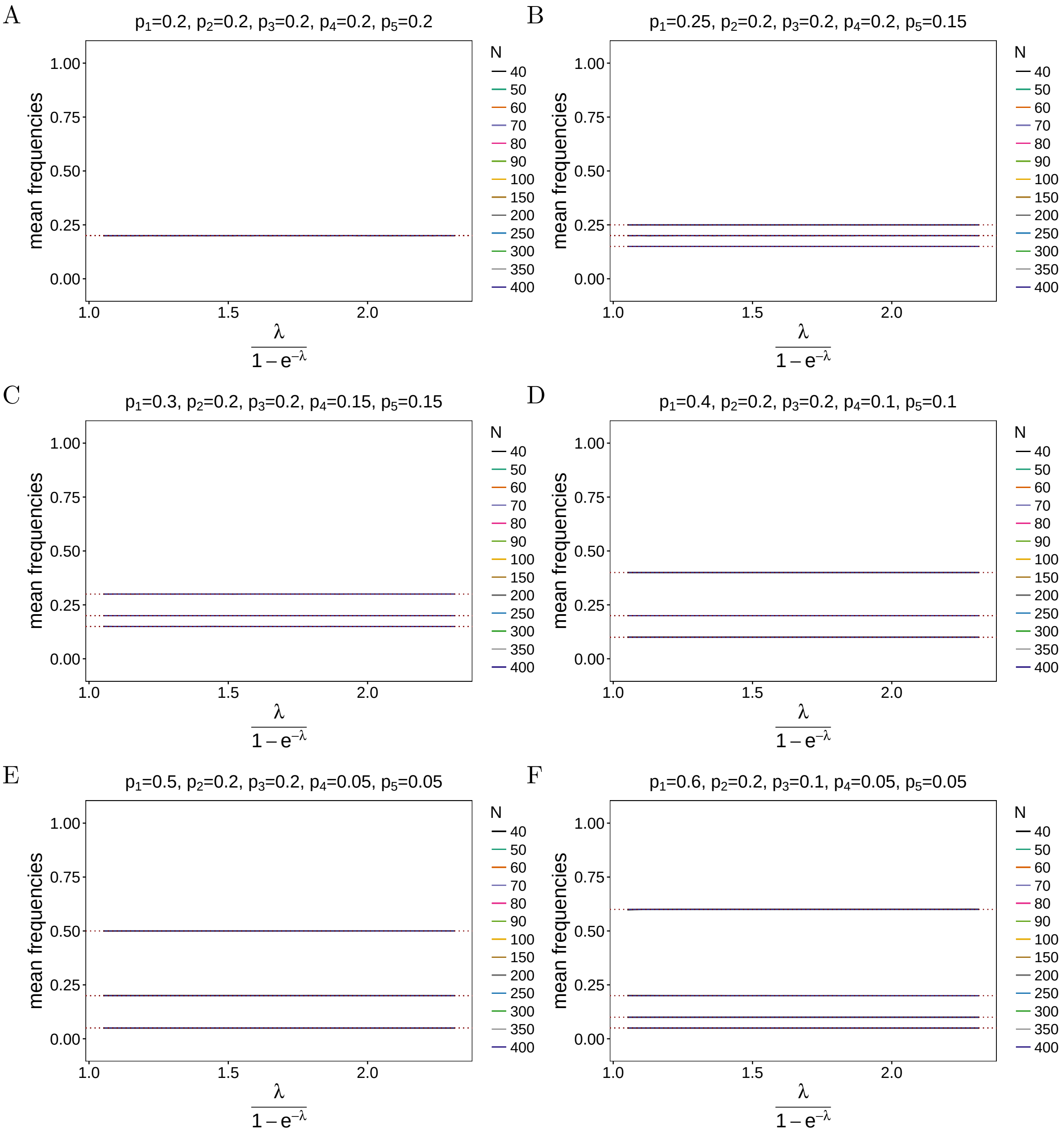

Figure 19: See Fig 16.

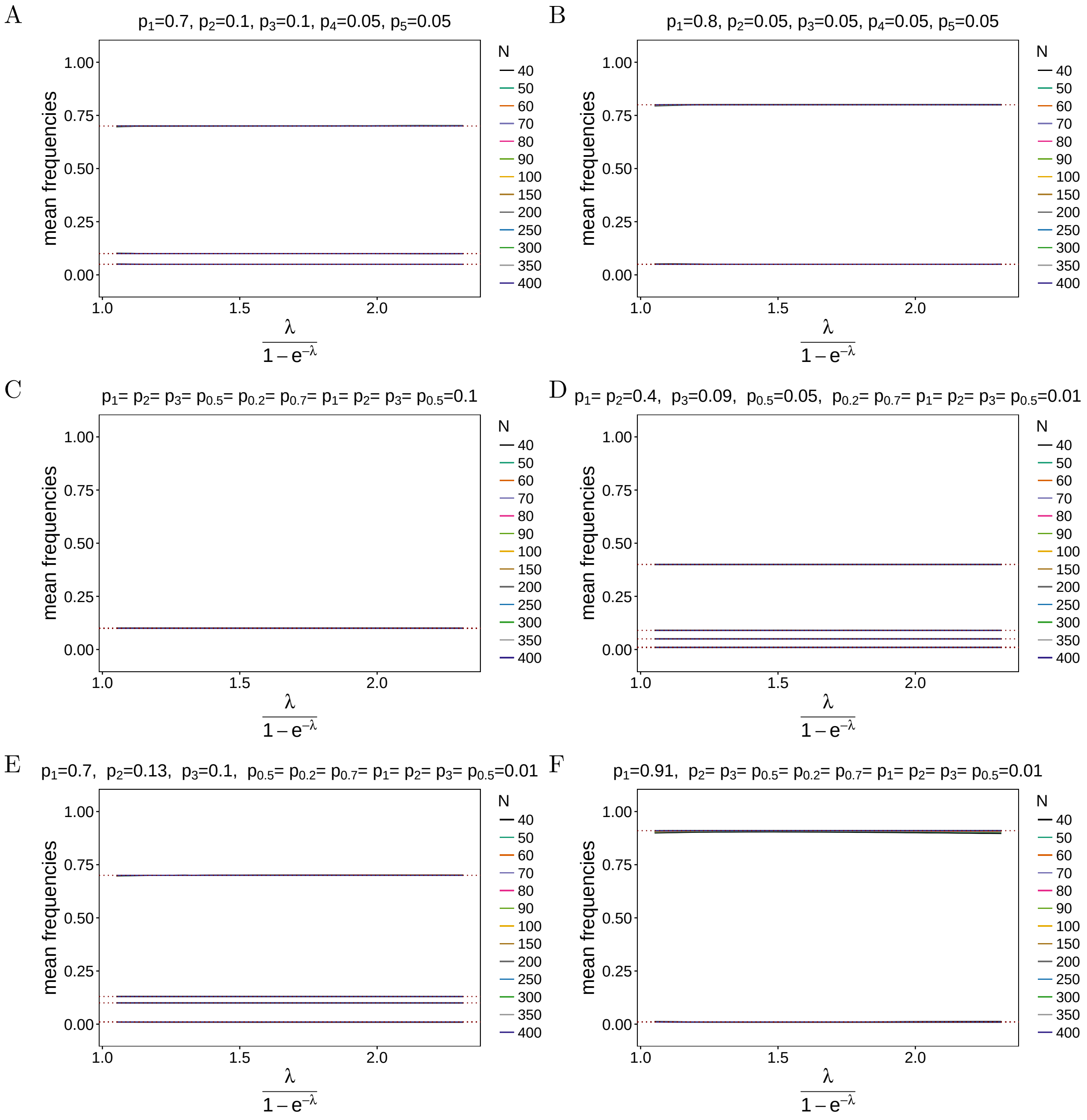

Figure 20: See Fig 16.

E Variance of frequency estimates

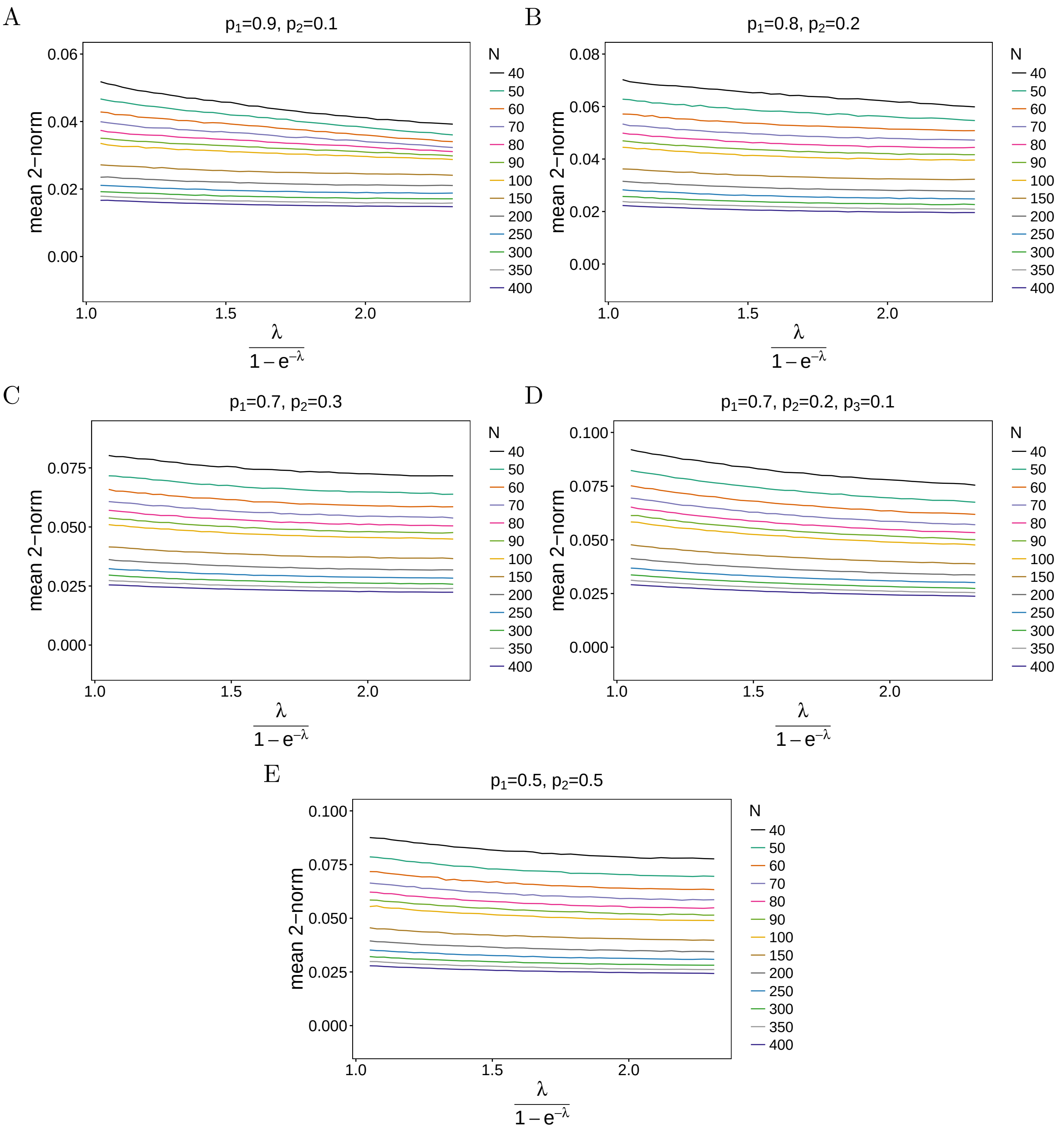

Figure 21: **Mean 2-norm of $\boldsymbol{p} - \hat{\boldsymbol{p}}$.** (A)-(E) Shown is the mean 2-norm of the difference $\boldsymbol{p} - \hat{\boldsymbol{p}}$ of the true frequencies and its MLEs based on simulated data created by the conditional Poisson model. For each parameter combination $K = 100\,000$ data sets were simulated. Each panel assumes different lineage-frequency distributions $\boldsymbol{p}$ shown at the top of each panel. Colored lines correspond to different sample sizes N.

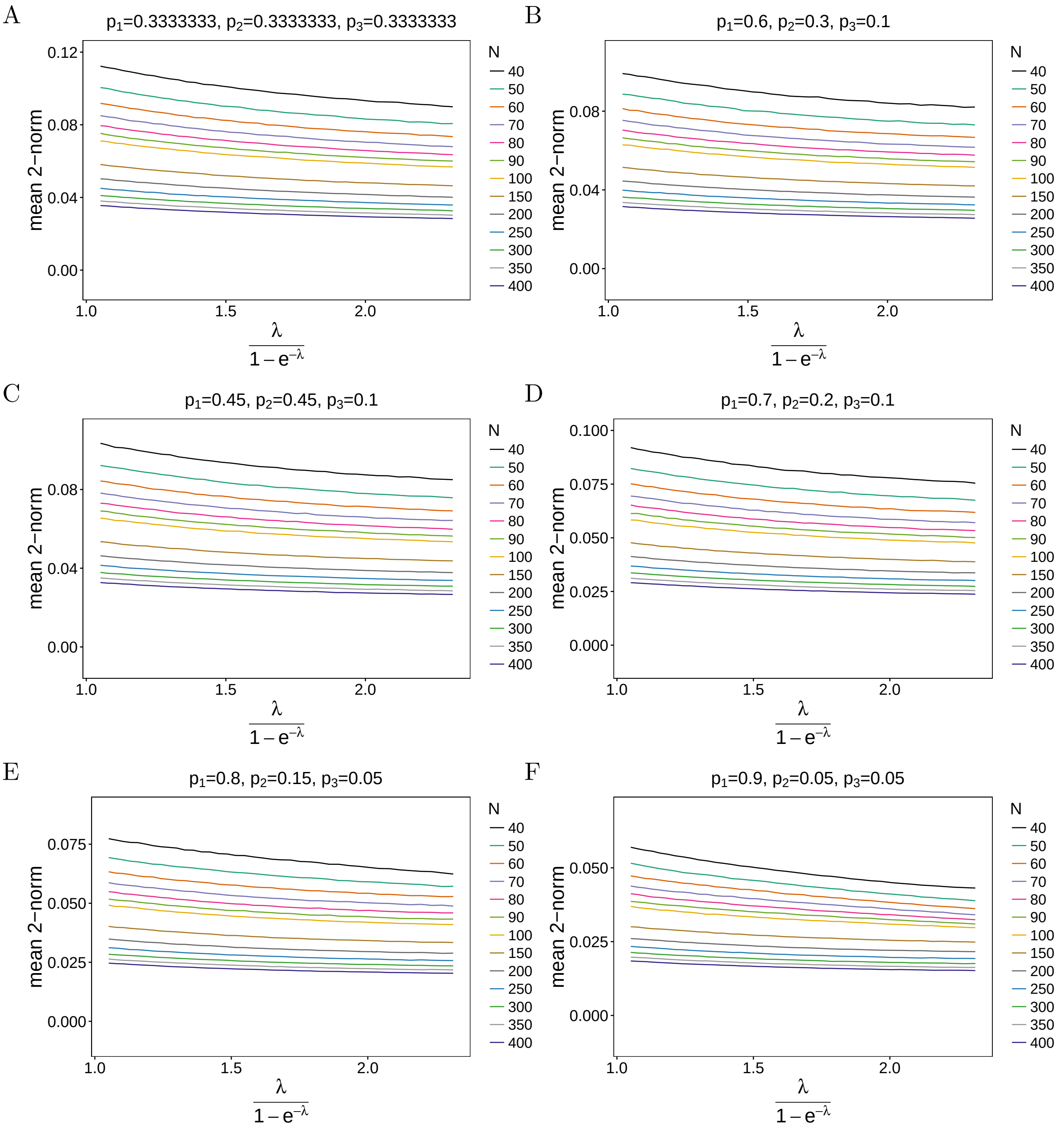

Figure 22: See Fig 21.

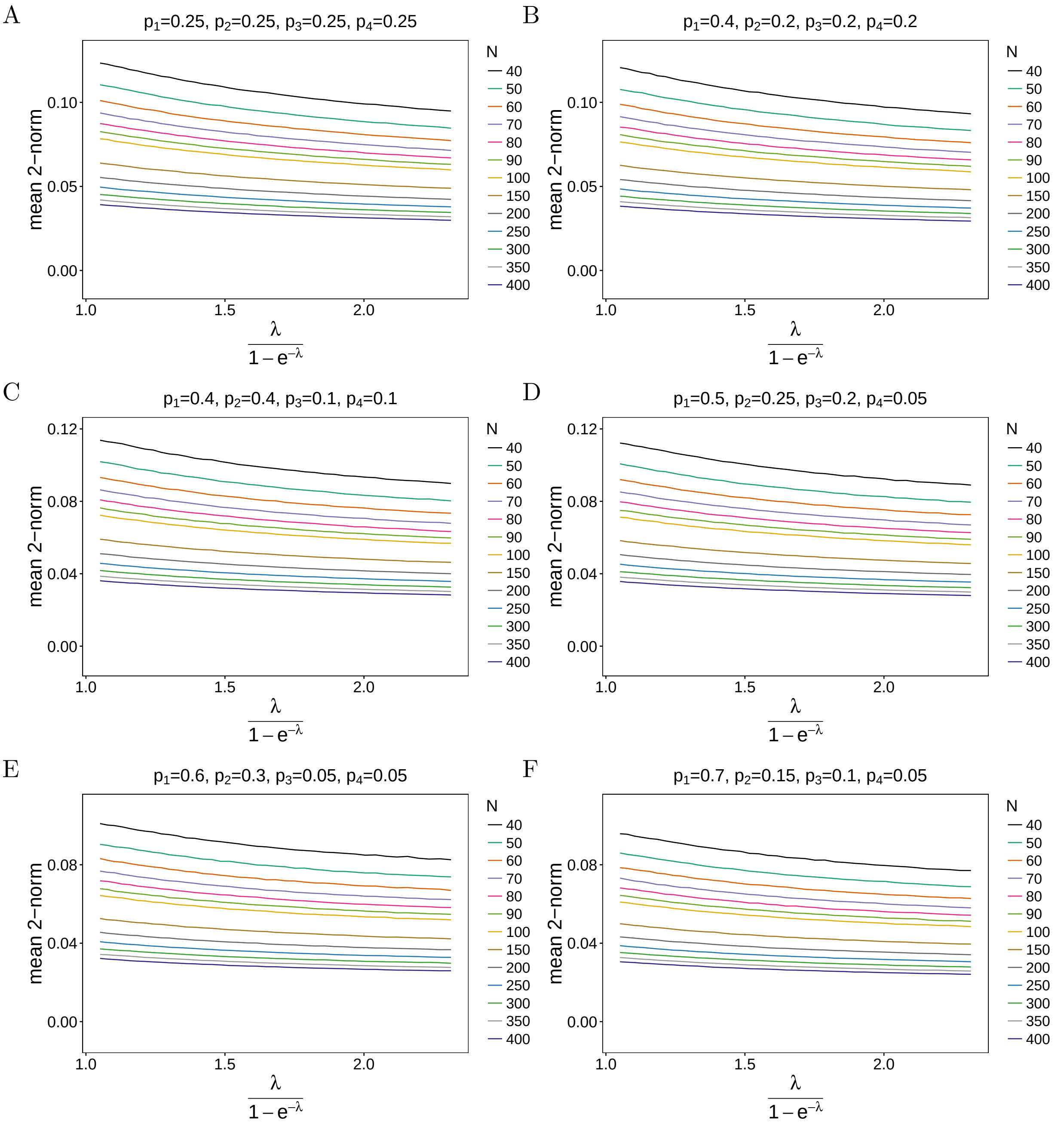

Figure 23: See Fig 21.

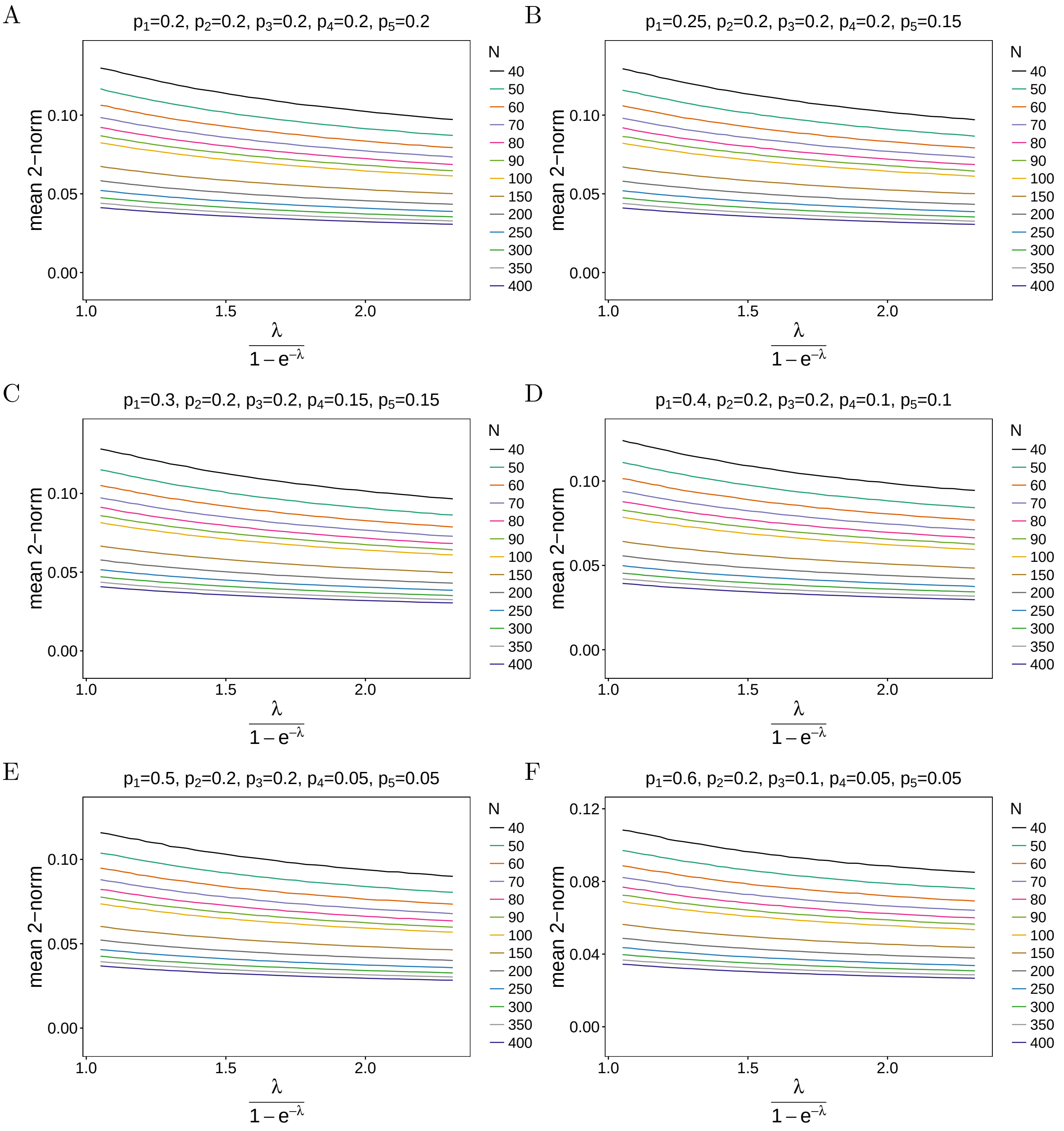

Figure 24: See Fig 21.

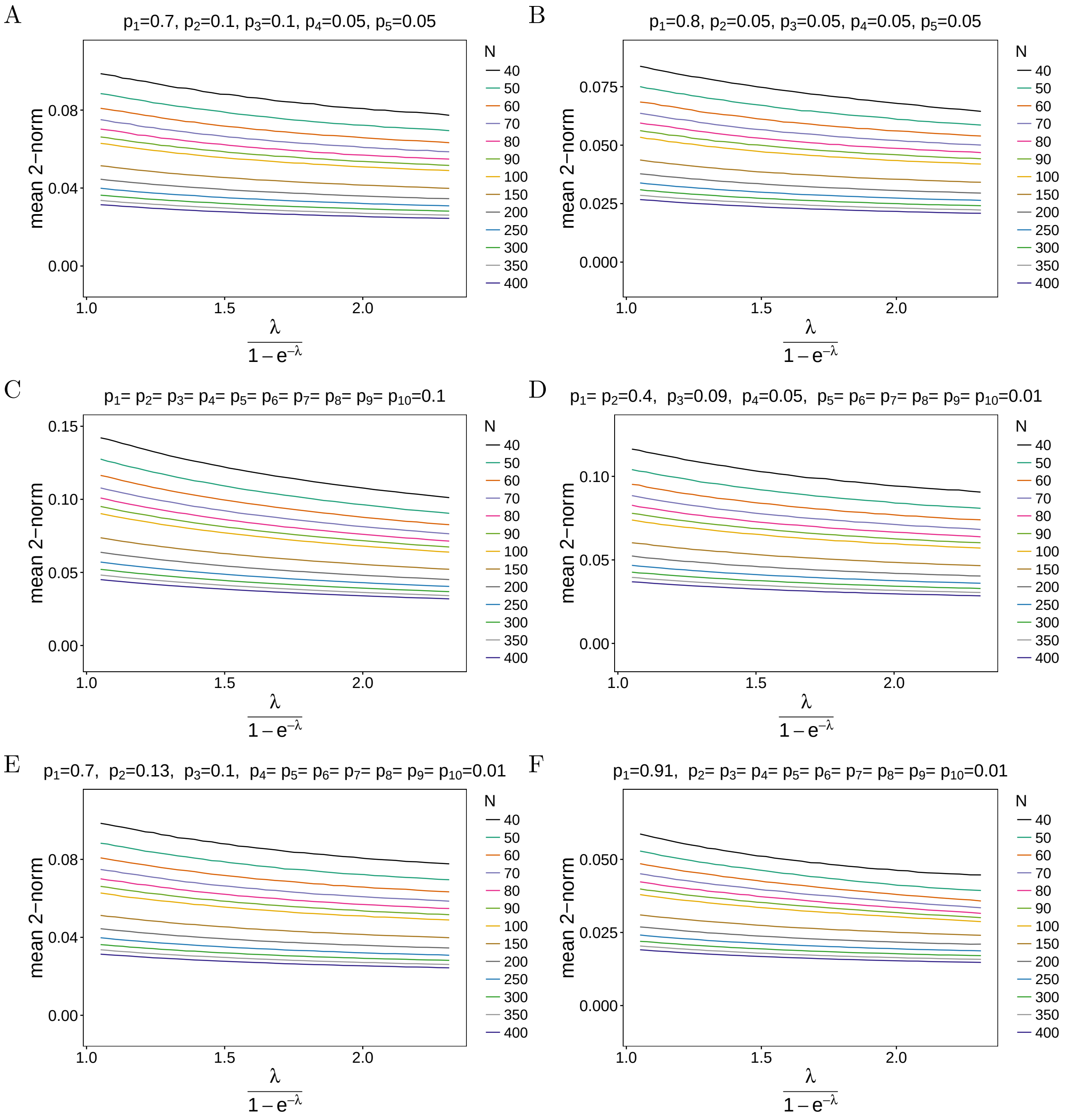

Figure 25: See Fig 21.

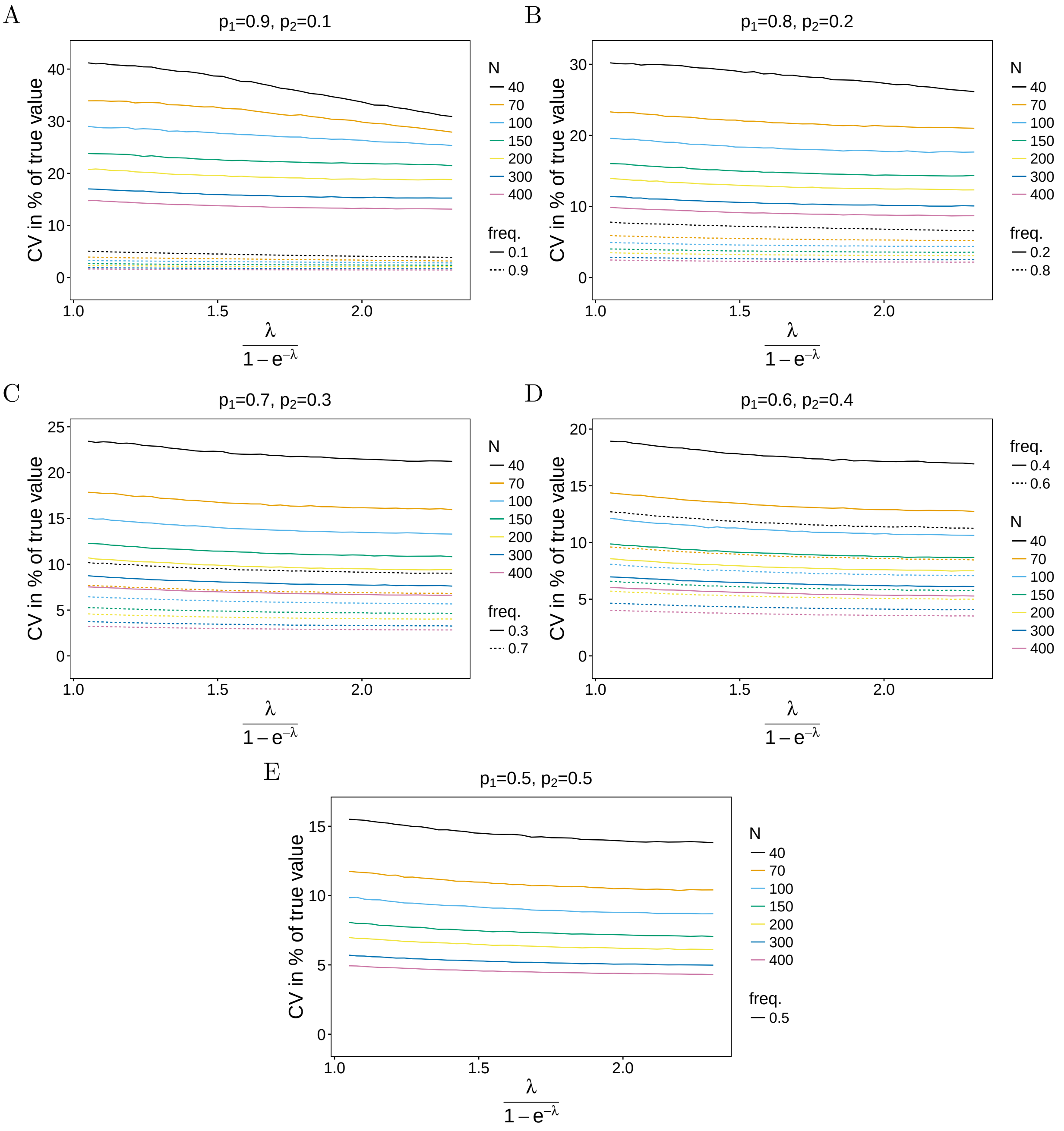

Figure 26: **Coefficient of variation.** (A)-(E) Shown are the coefficients of variation of the frequency estimates $\hat{p}_1, \ldots, \hat{p}_n$ in % of the true parameters based on simulations based on simulated data created by the conditional Poisson model. For each parameter combination $K = 100\,000$ data sets were simulated. Each panel assumes different lineage-frequency distributions $\boldsymbol{p}$ shown at the top of each panel. Colored lines correspond to different sample sizes N. Dashing corresponds to different frequencies.

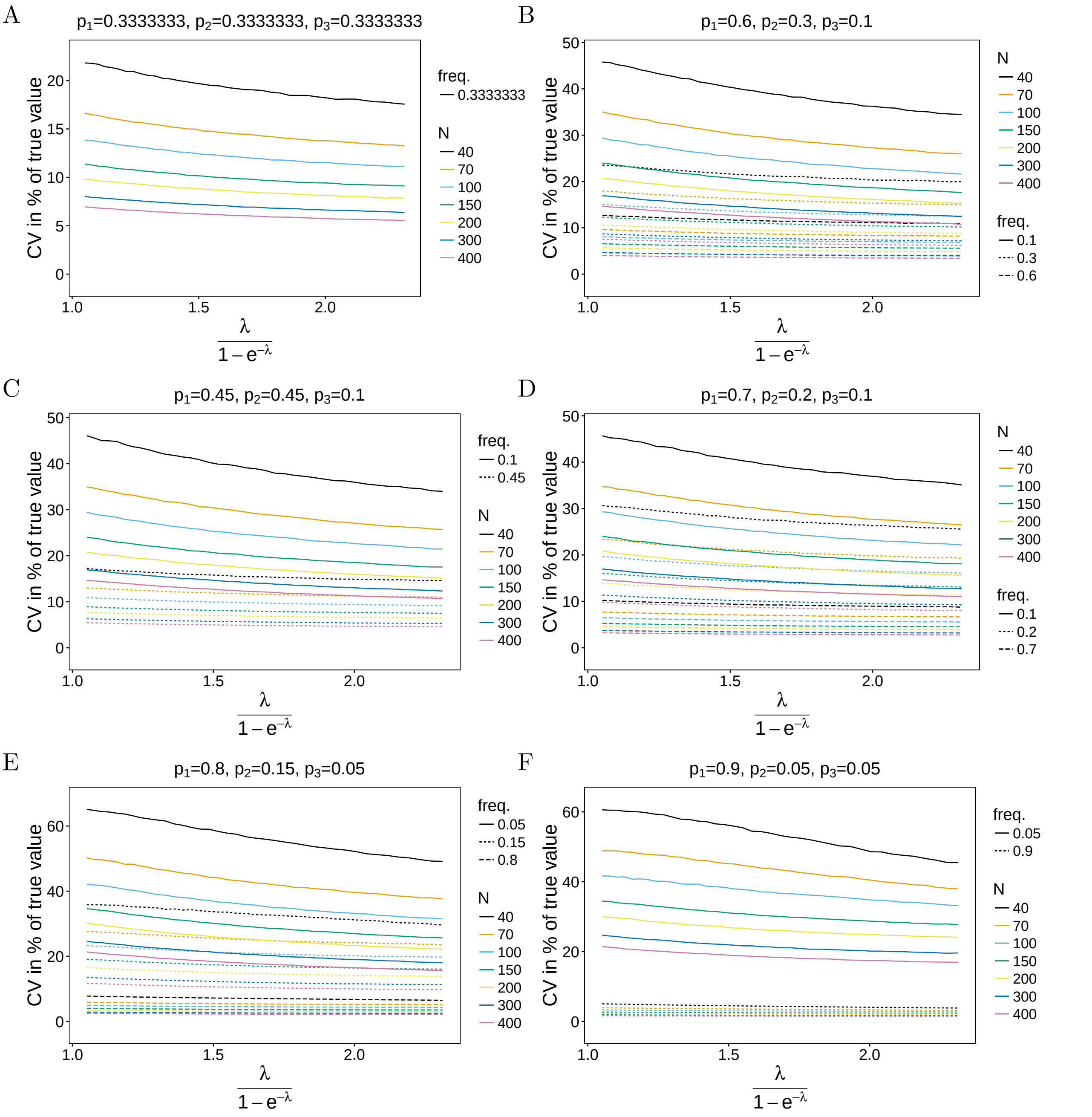

Figure 27: See Fig 26.

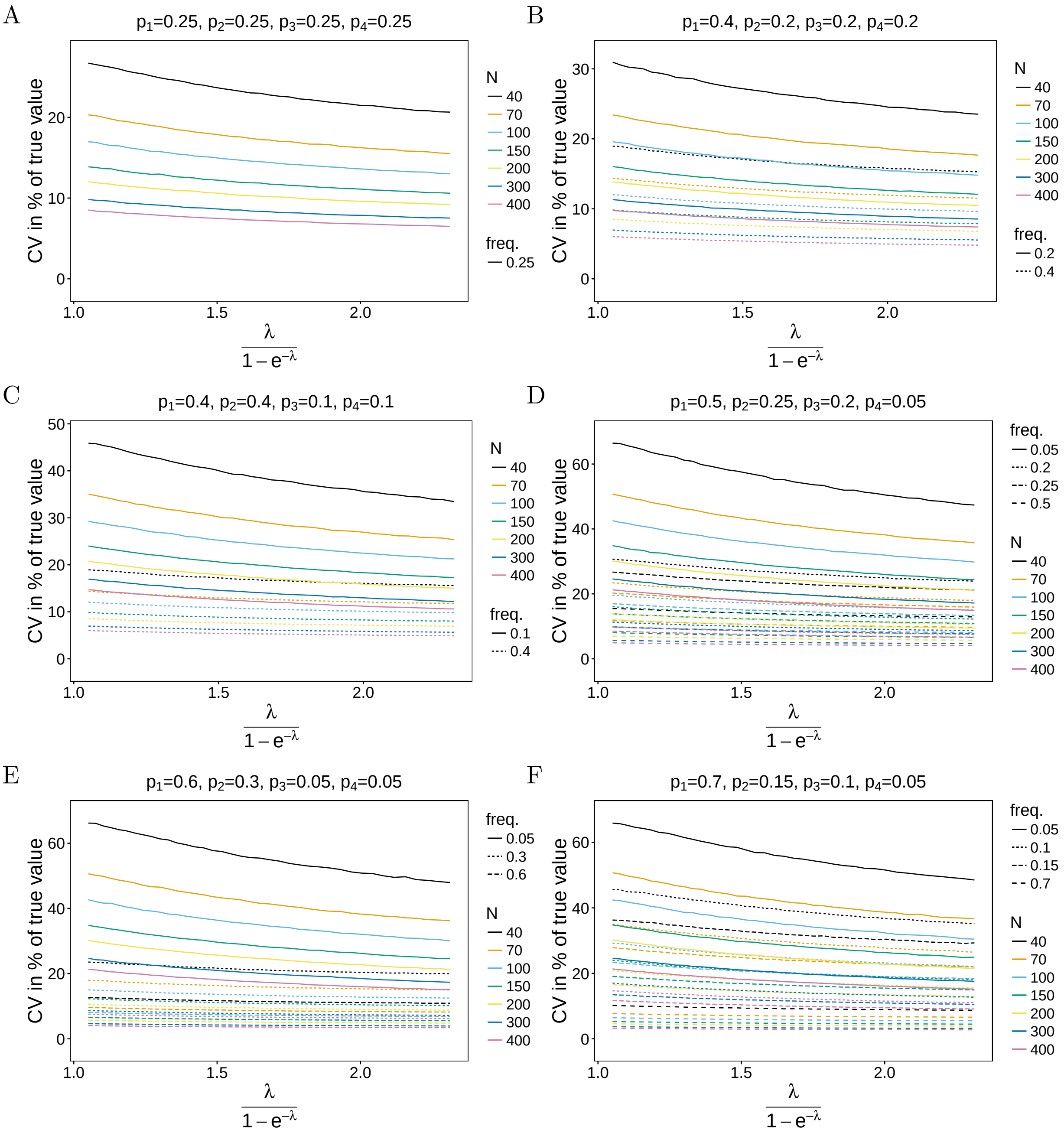

Figure 28: See Fig 26.

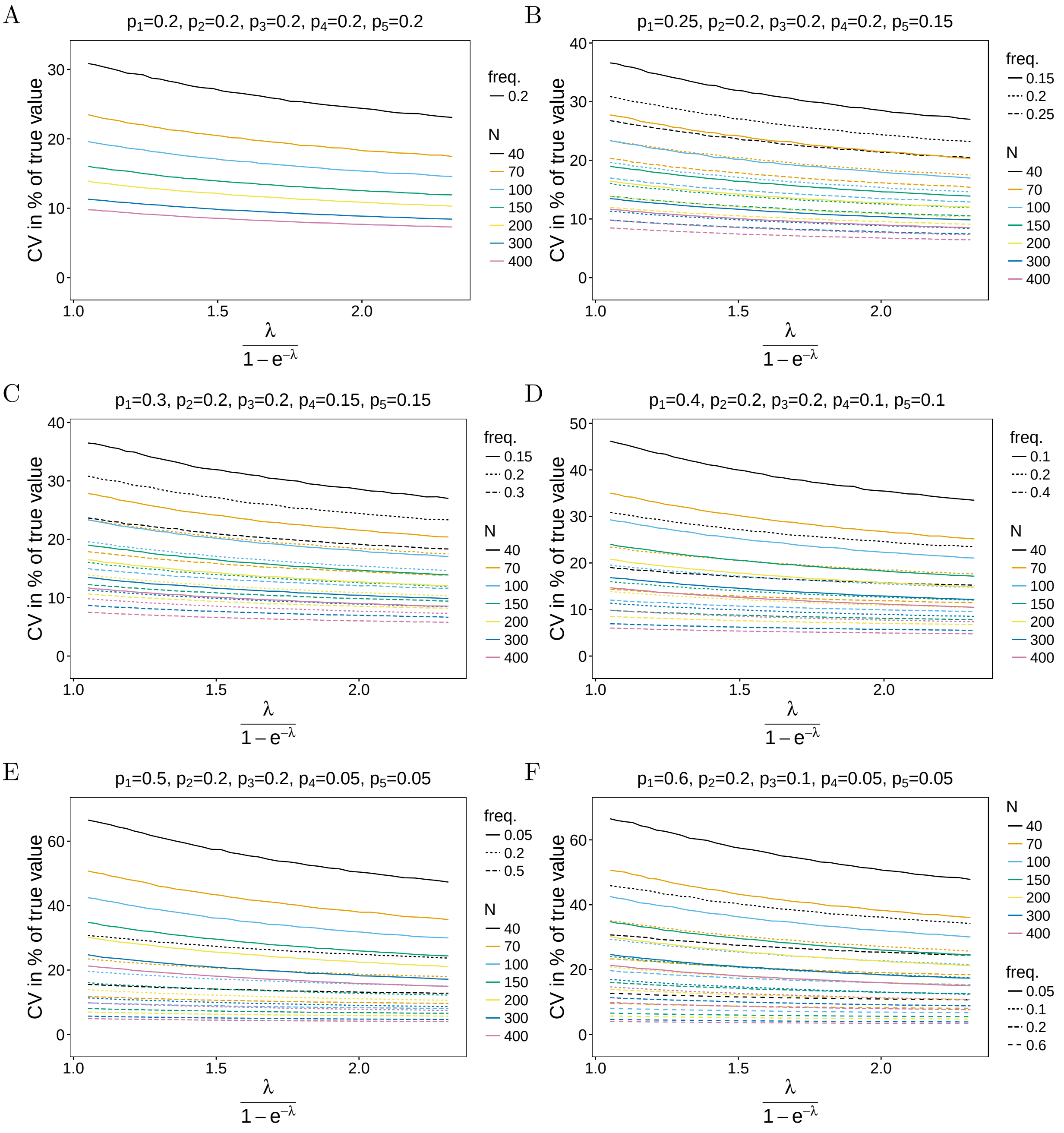

Figure 29: See Fig 26.

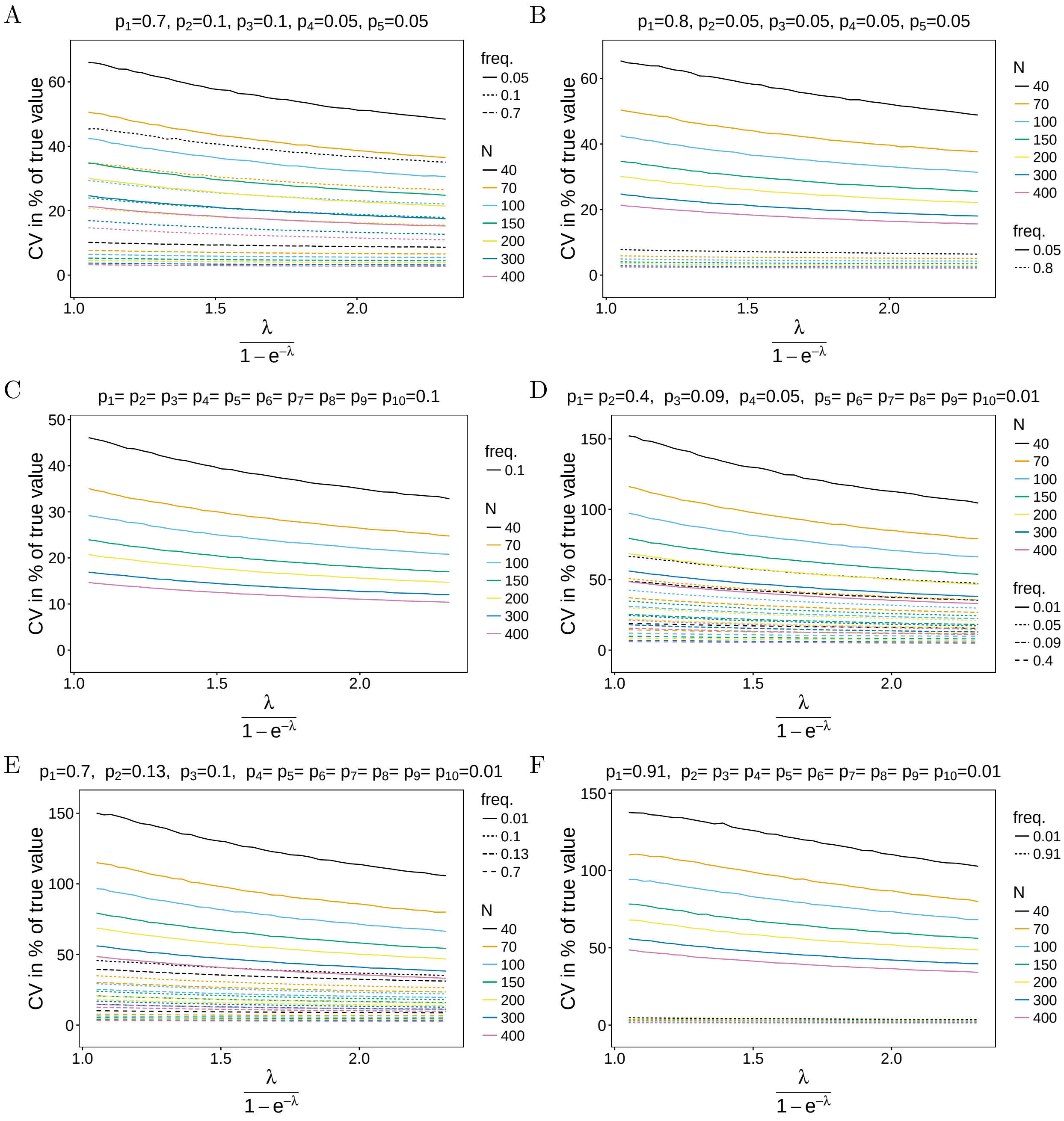

Figure 30: See Fig 26.

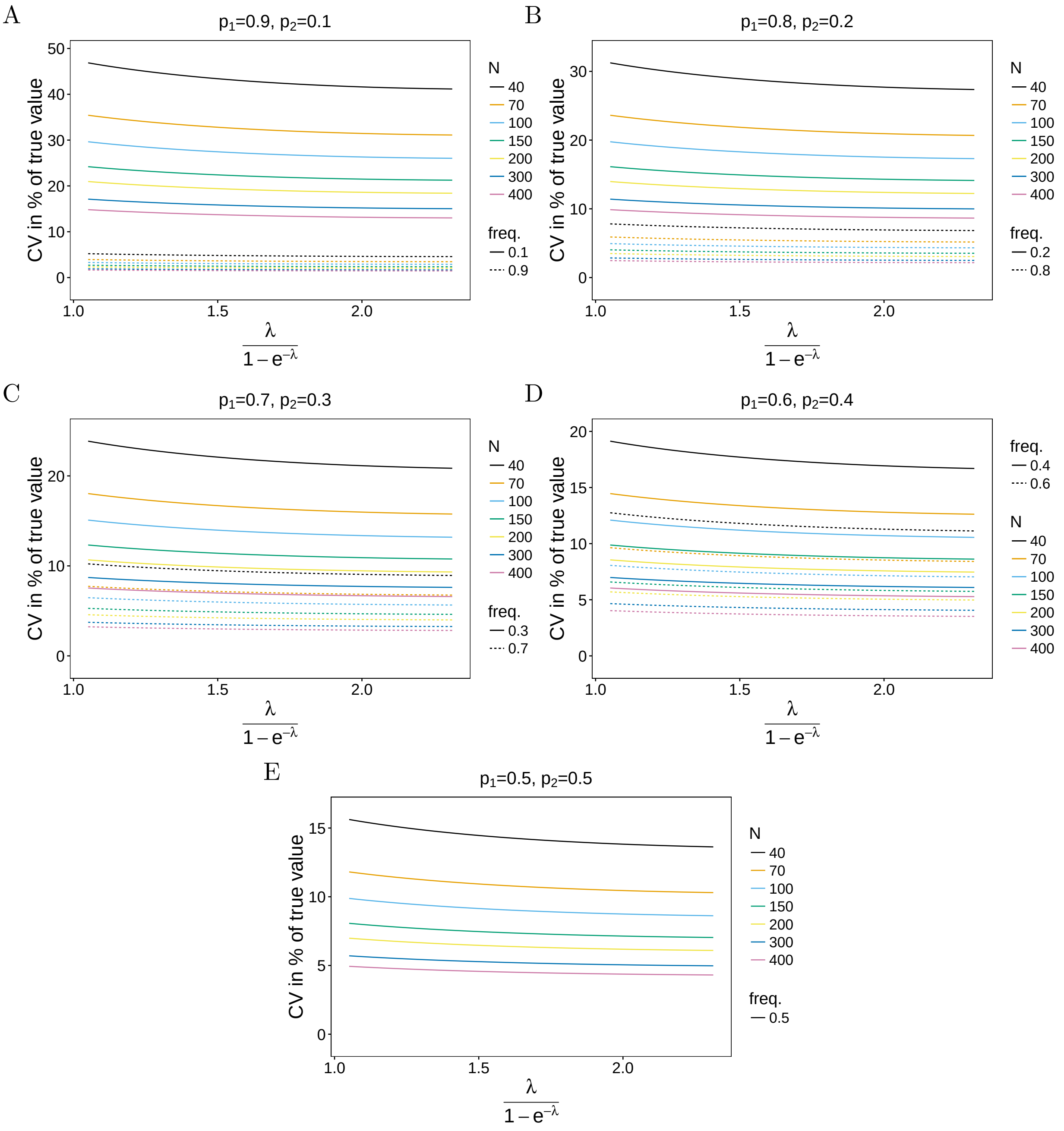

Figure 31: **Coefficient of variation.** (A)-(E) Shown are the theoretical predictions for the coefficients of variation of the frequency estimates $\hat{p}_1, \ldots, \hat{p}_n$ in % of the true parameters based the Cramér-Rao lower bounds. Each panel assumes different lineage-frequency distributions $\boldsymbol{p}$ shown at the top of each panel. Colored lines correspond to different sample sizes N. Dashing corresponds to different frequencies. Dashing corresponds to different frequencies.

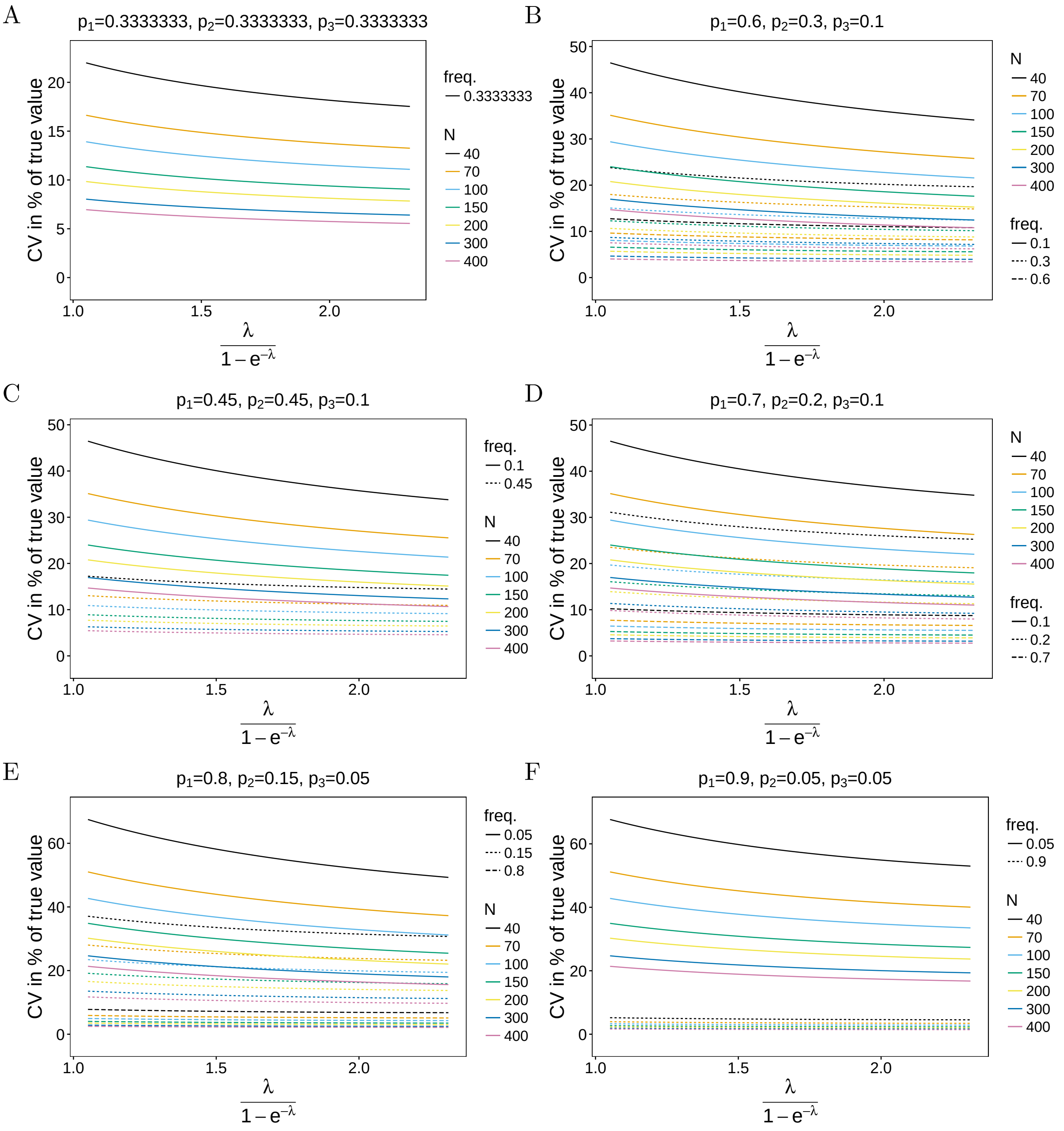

Figure 32: See Fig 31.

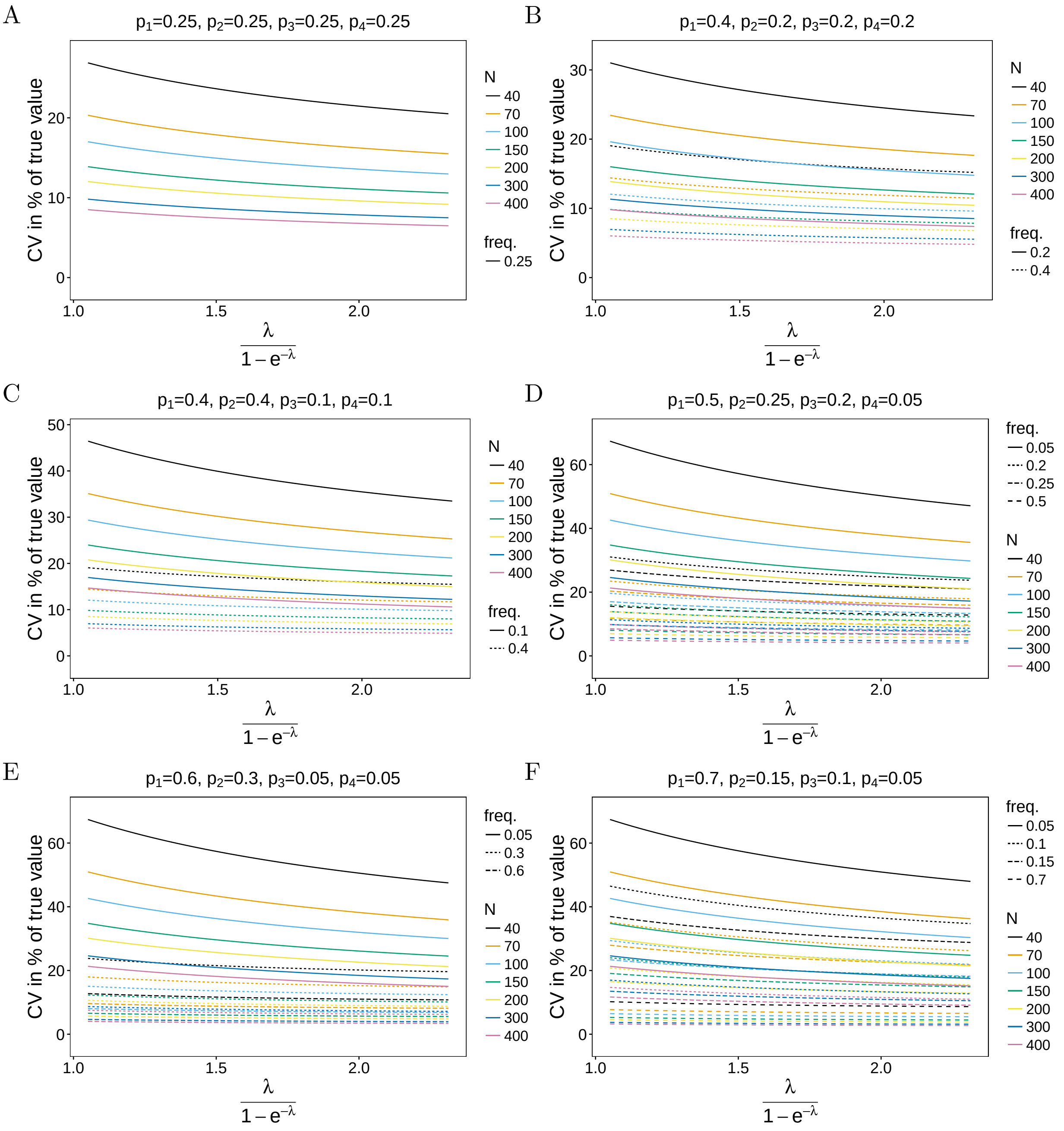

Figure 33: See Fig 31.

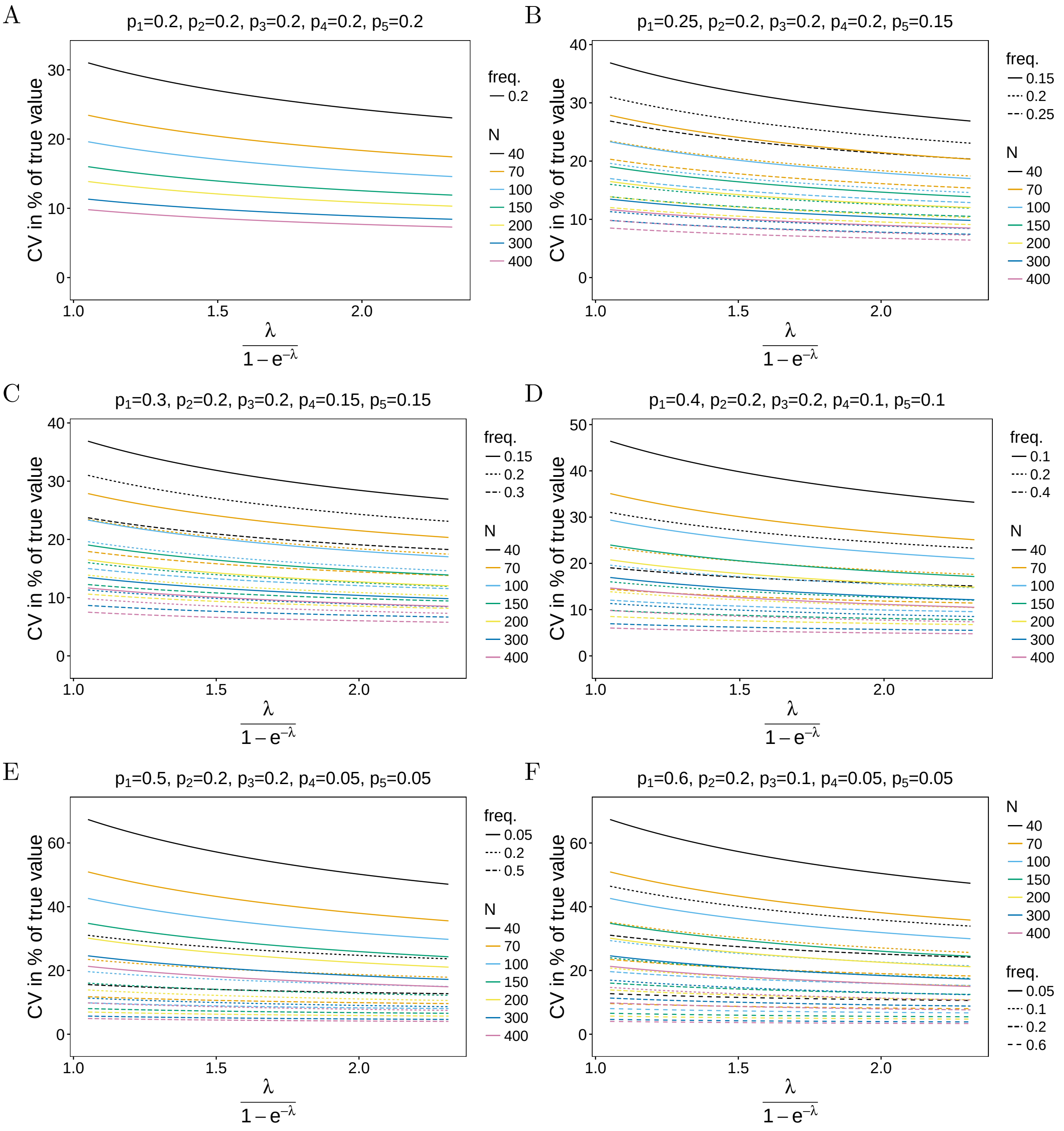

Figure 34: See Fig 31.

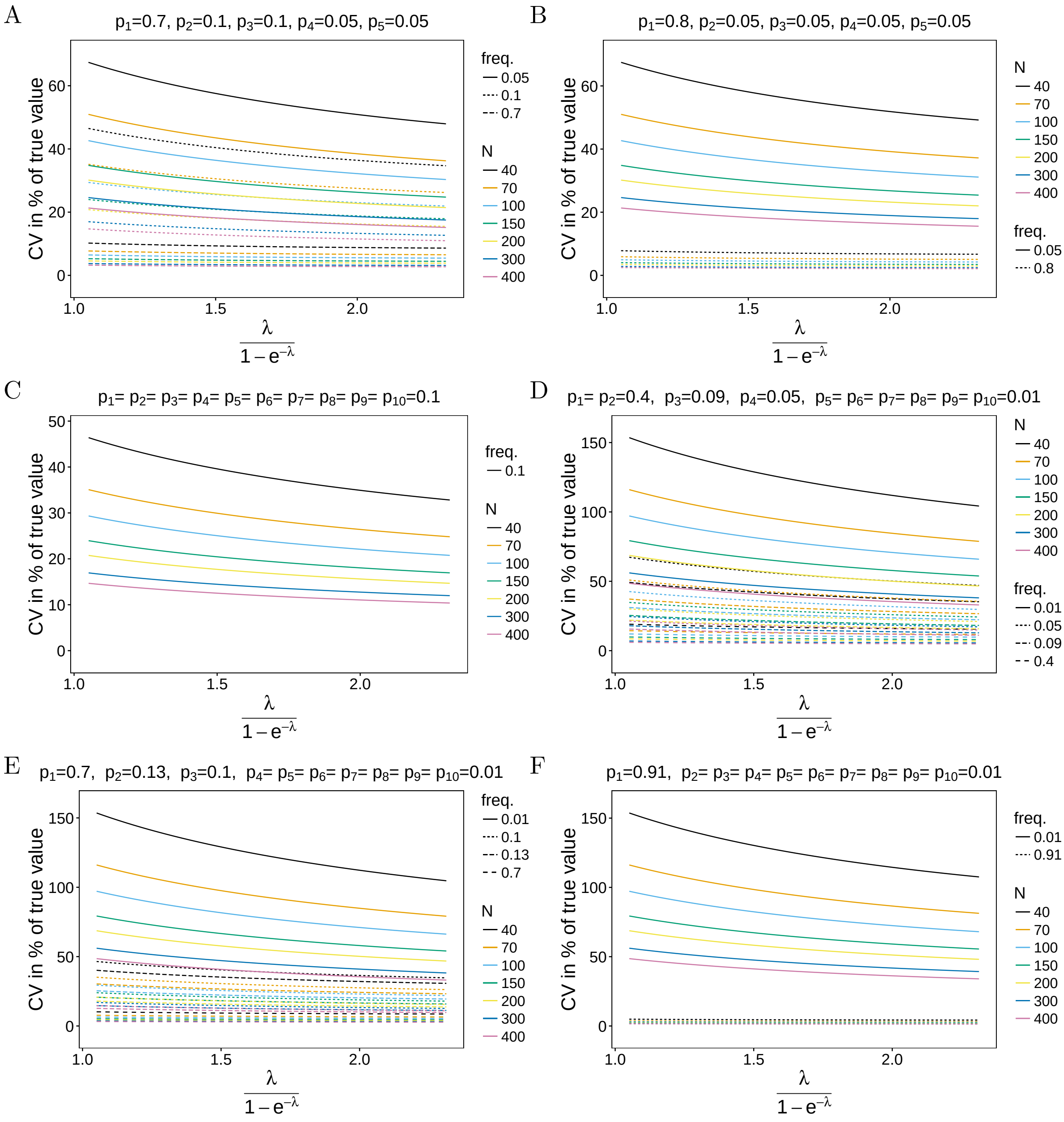

Figure 35: See Fig 31.

F Model violations

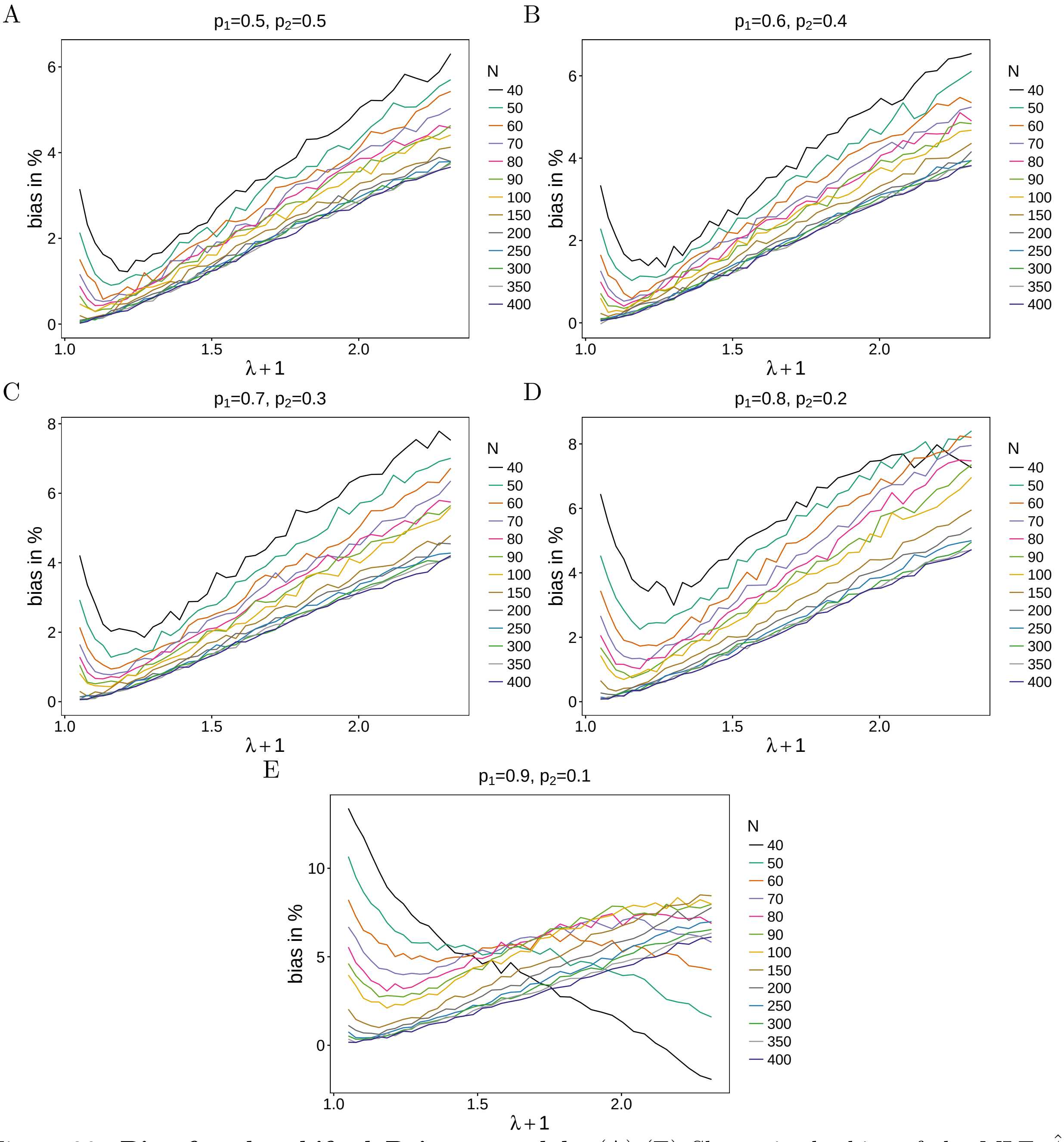

Figure 36: **Bias for the shifted Poisson model.** (A)-(E) Shown is the bias of the MLE $\hat{\psi}$ in percent of the true parameter $\psi = \lambda + 1$ as a function of the true parameter ψ based on simulated data created by the shifted Poisson model. For each parameter combination $K = 10\,000$ data sets were simulated. Each panel assumes different lineage-frequency distributions $\boldsymbol{p}$ shown at the top of each panel. Colored lines correspond to different sample sizes N.

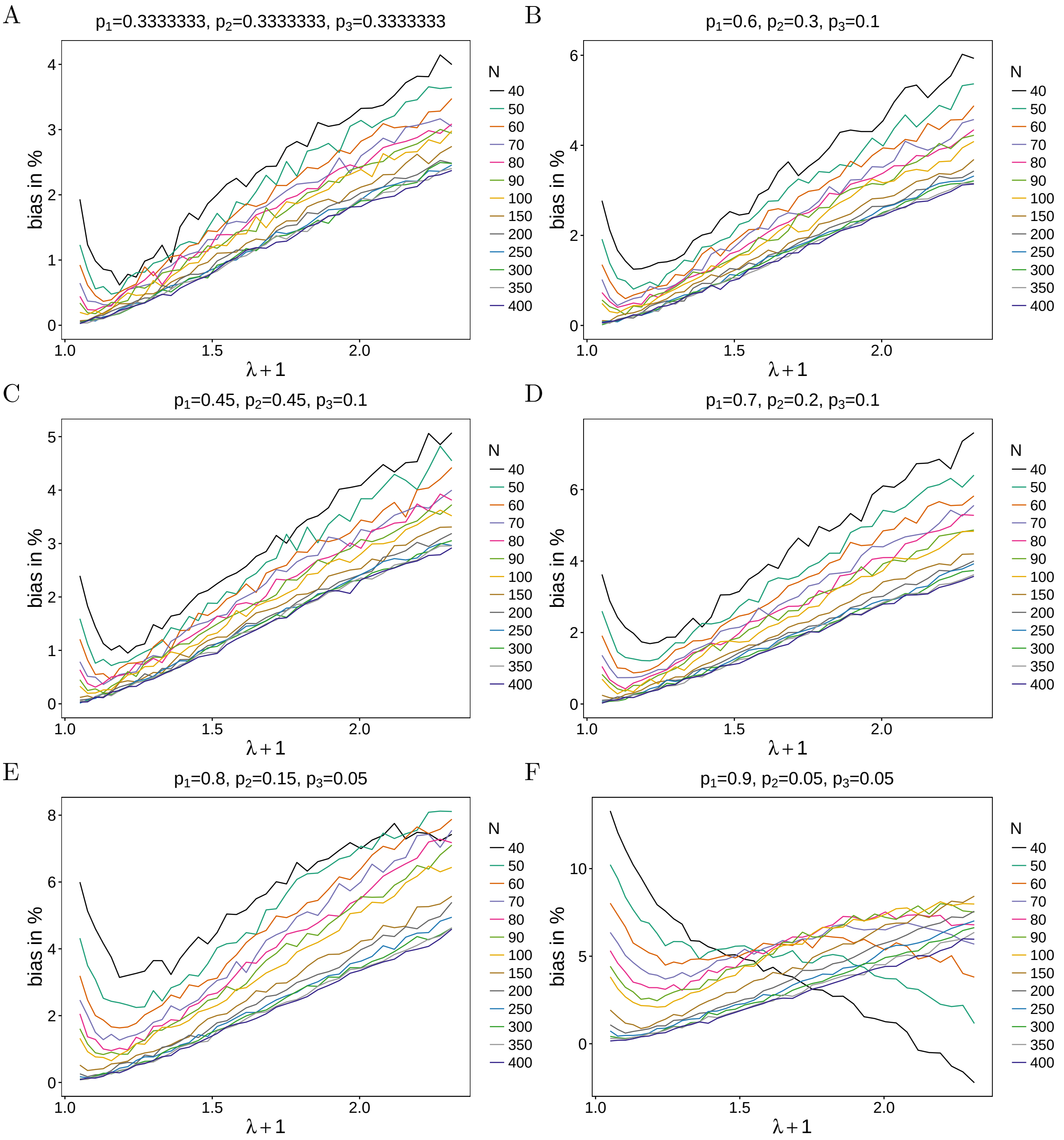

Figure 37: See Fig 36.

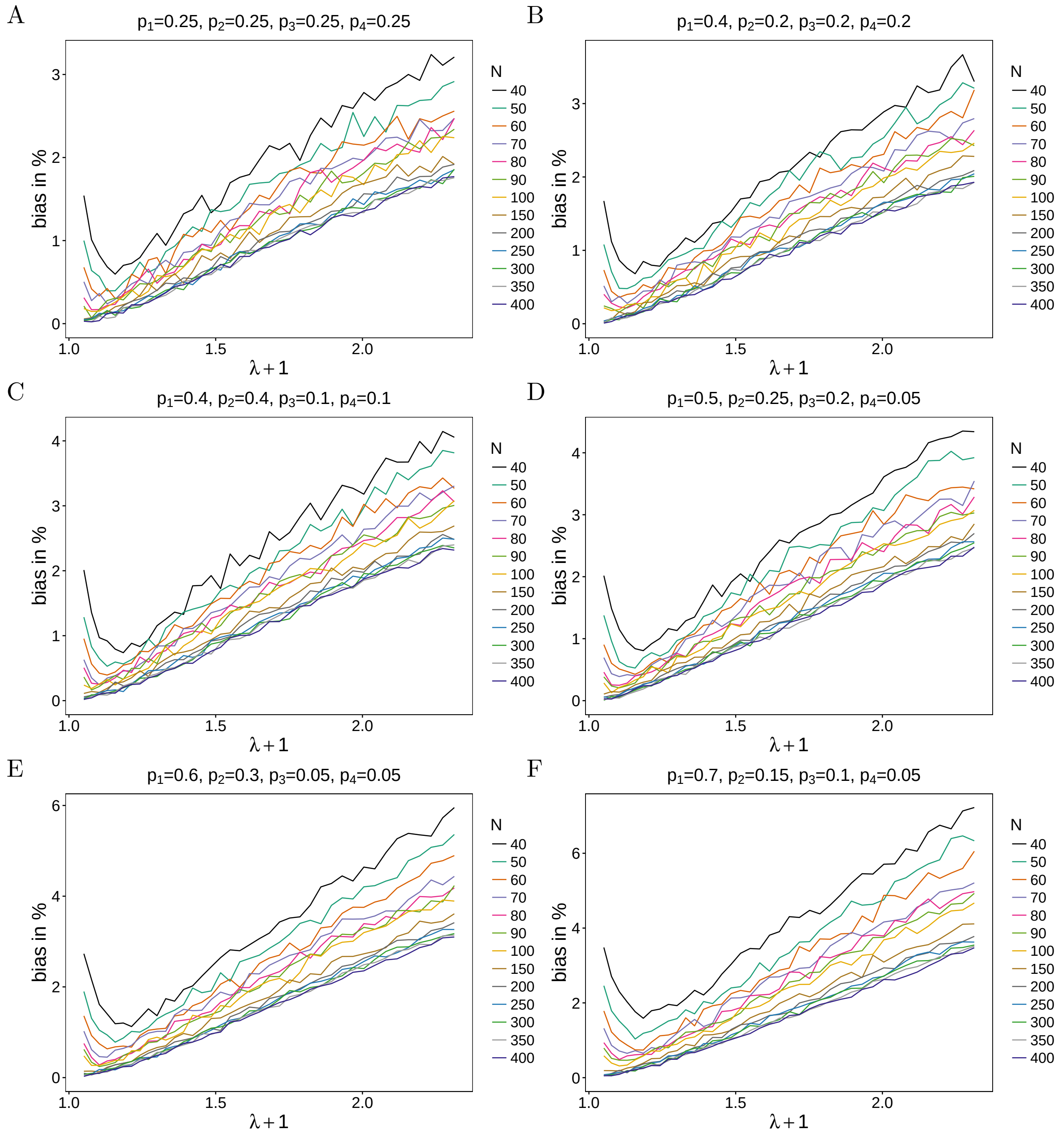

Figure 38: See Fig 36.

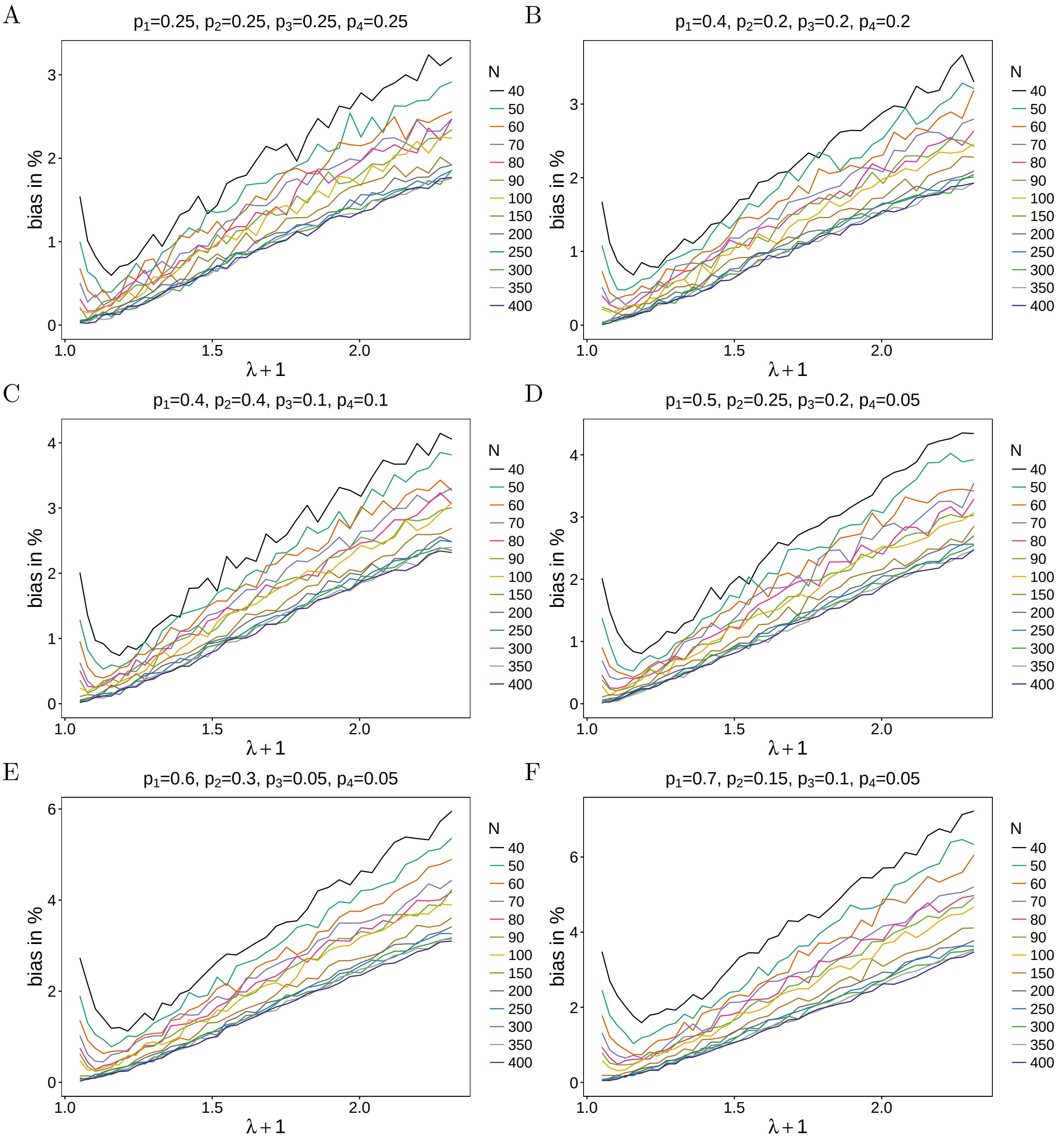

Figure 39: See Fig 36.

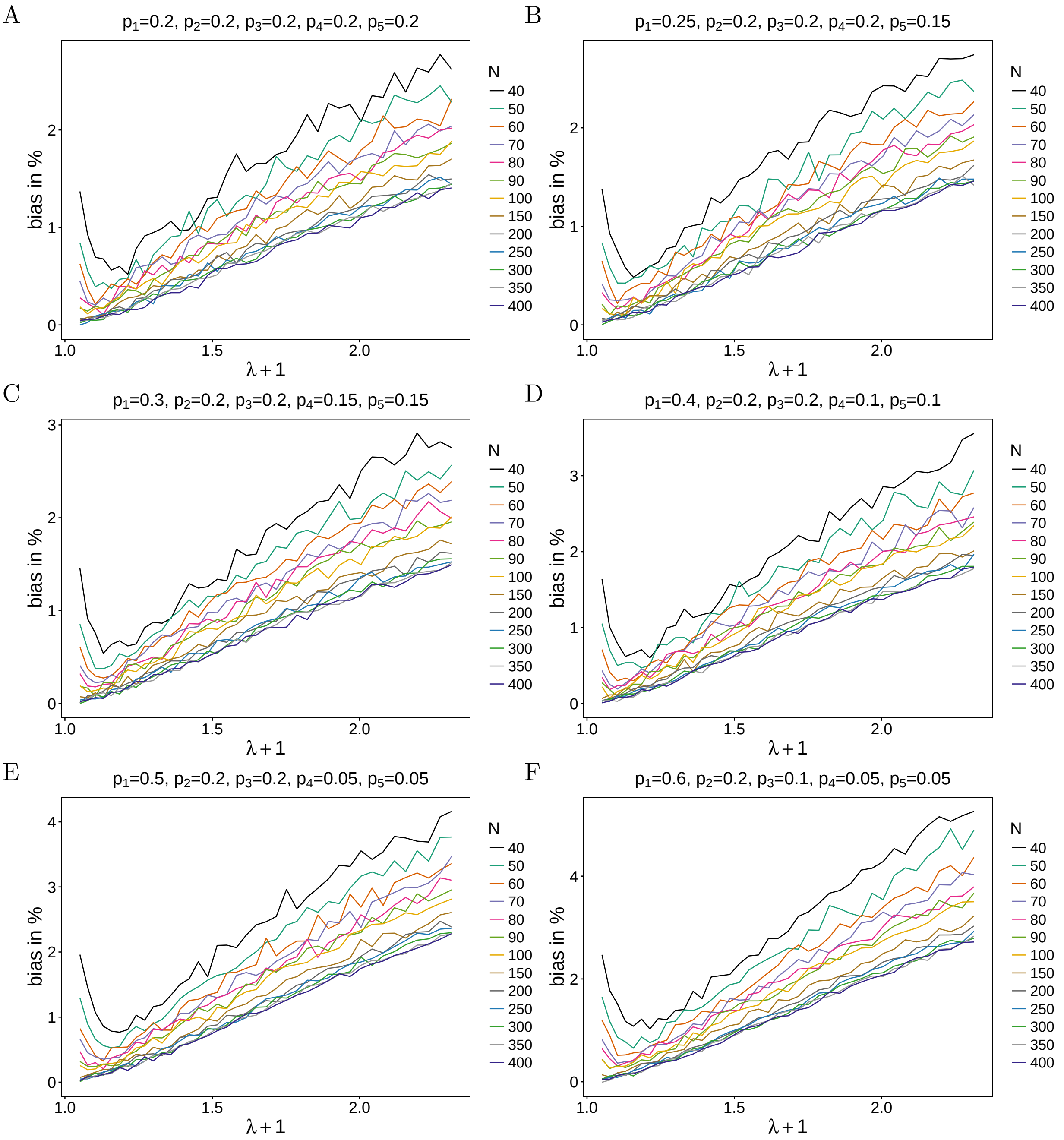

Figure 40: See Fig 36.

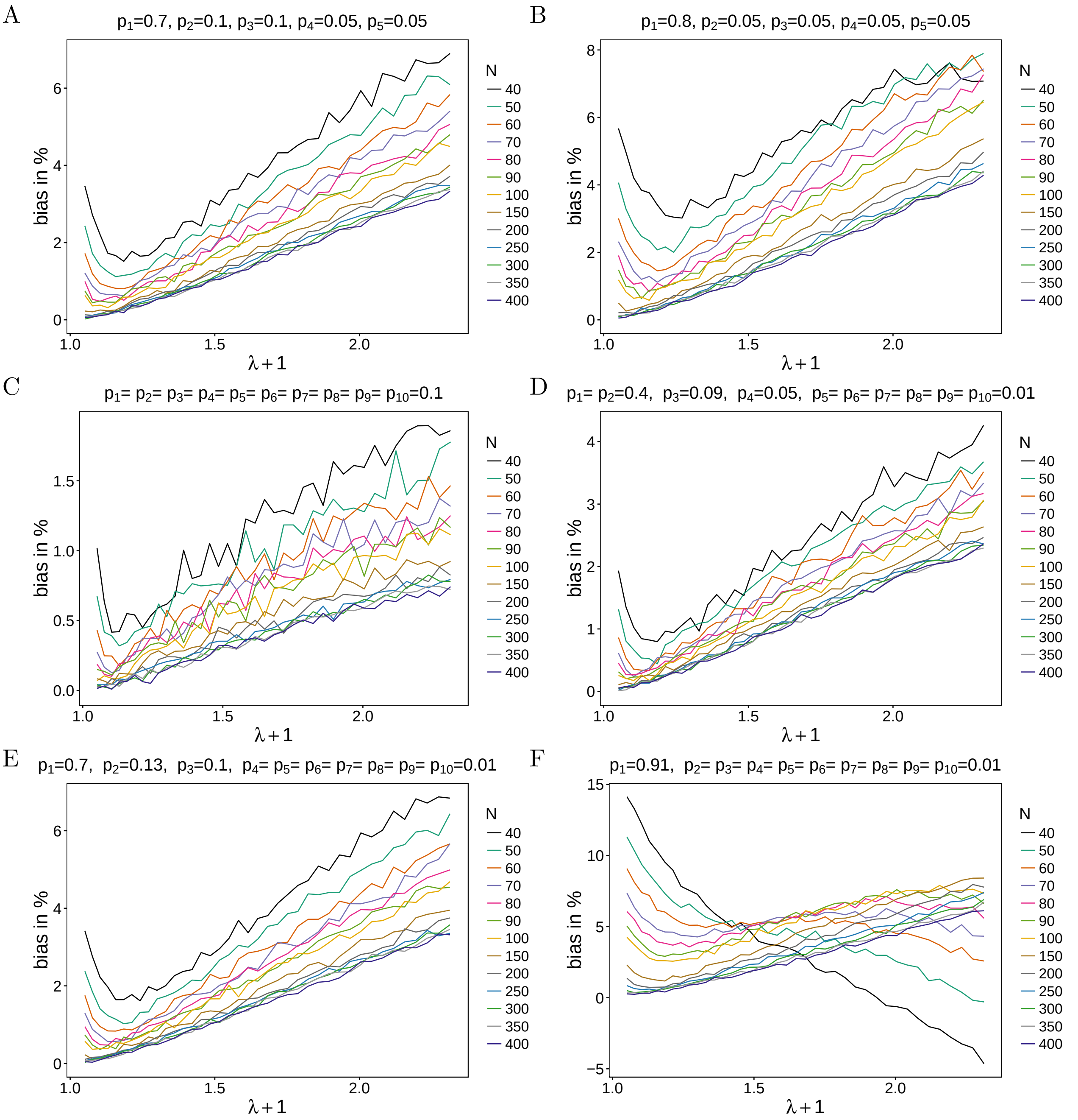

Figure 41: See Fig 36.

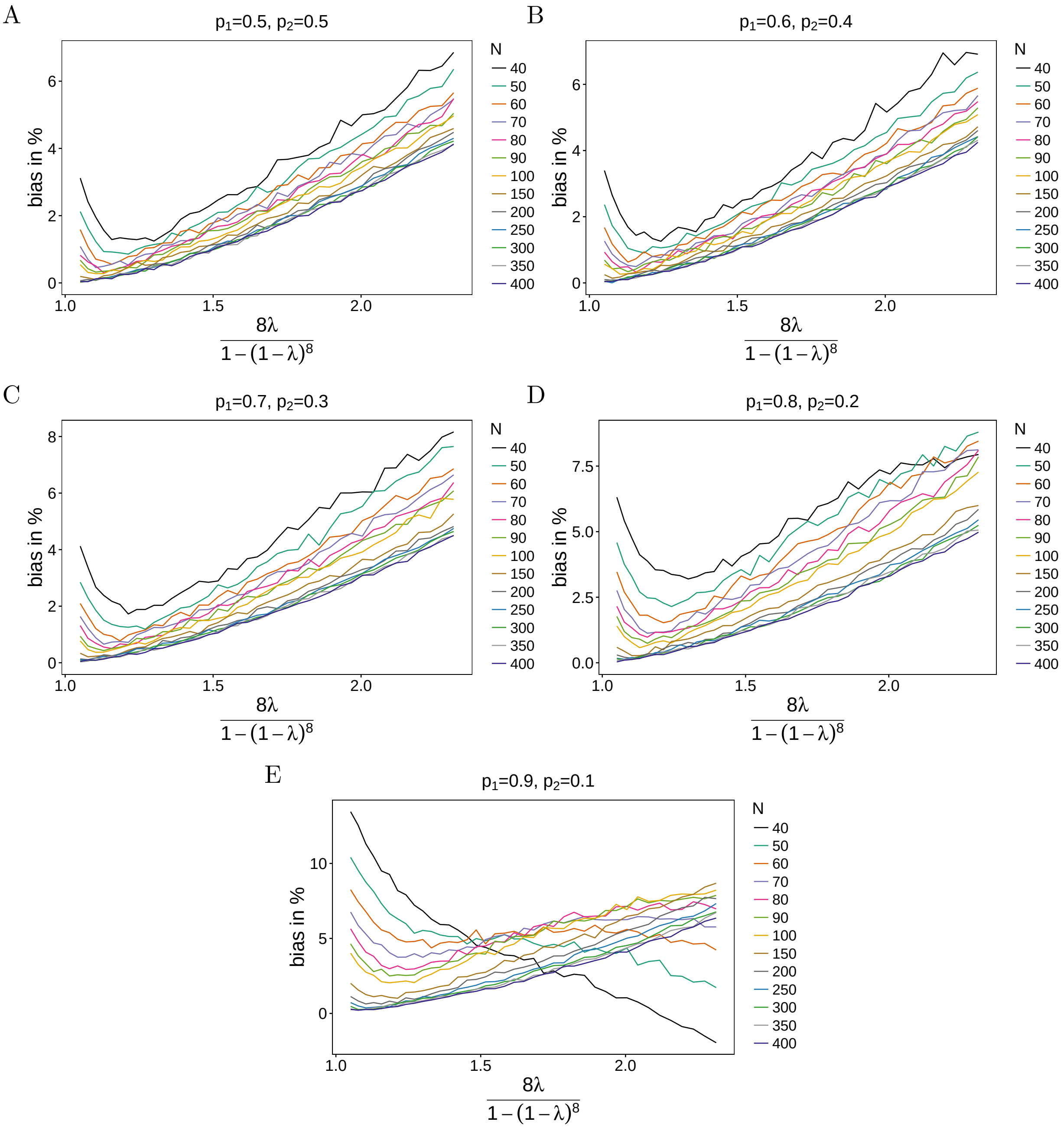

Figure 42: **Bias for the conditional binomial model.** (A)-(E) Shown is the bias of the MLE $\hat{\psi}$ in percent of the true parameter $\psi = \frac{8\lambda}{1-(1-\lambda)^8}$ as a function of the true parameter ψ based on simulated data created by the conditional binomial model. For each parameter combination $K = 10\,000$ data sets were simulated. Each panel assumes different lineage frequency distributions $\boldsymbol{p}$ shown at the top of each panel. Colored lines correspond to different sample sizes N.

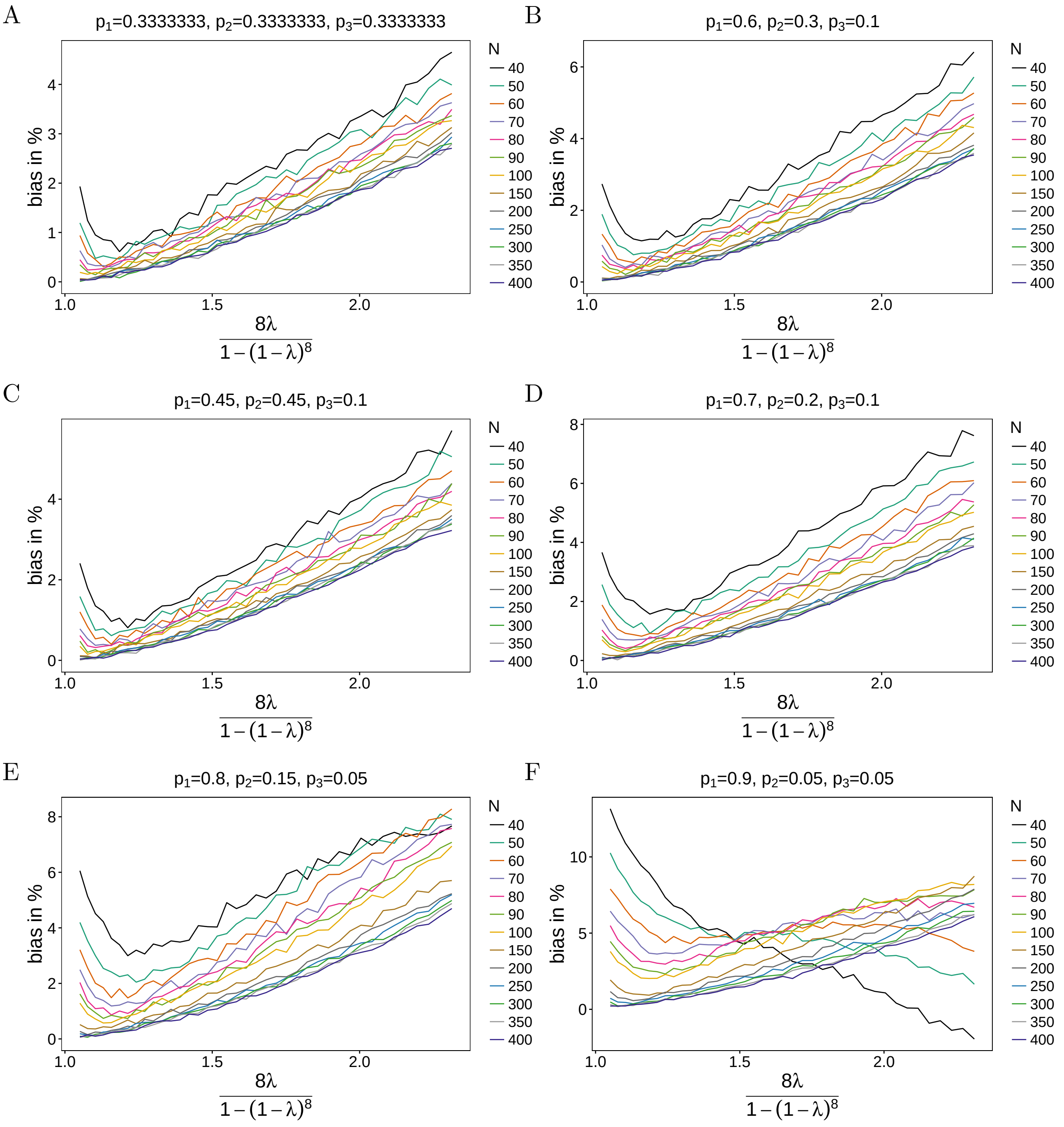

Figure 43: See Fig 42.

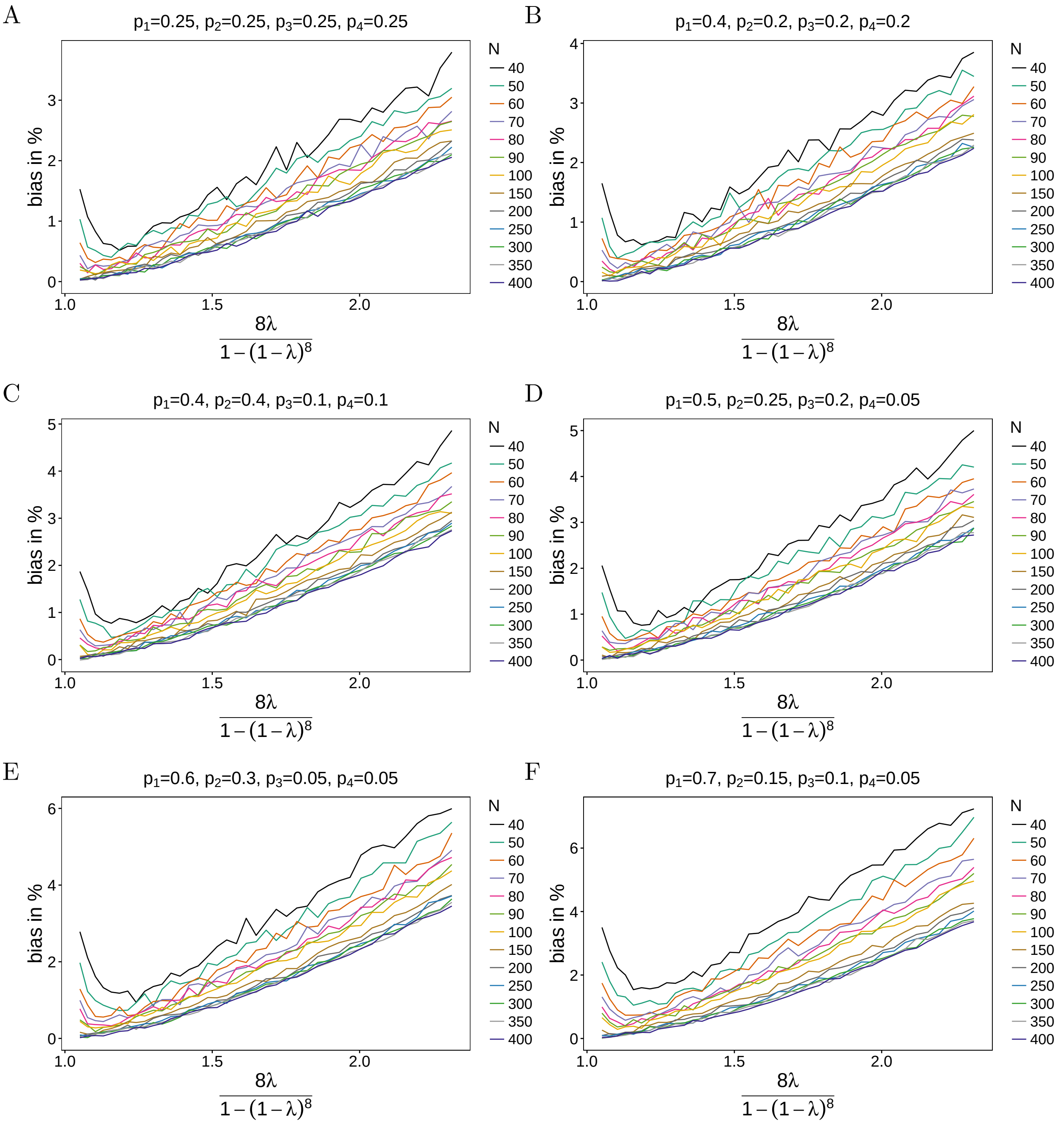

Figure 44: See Fig 42.

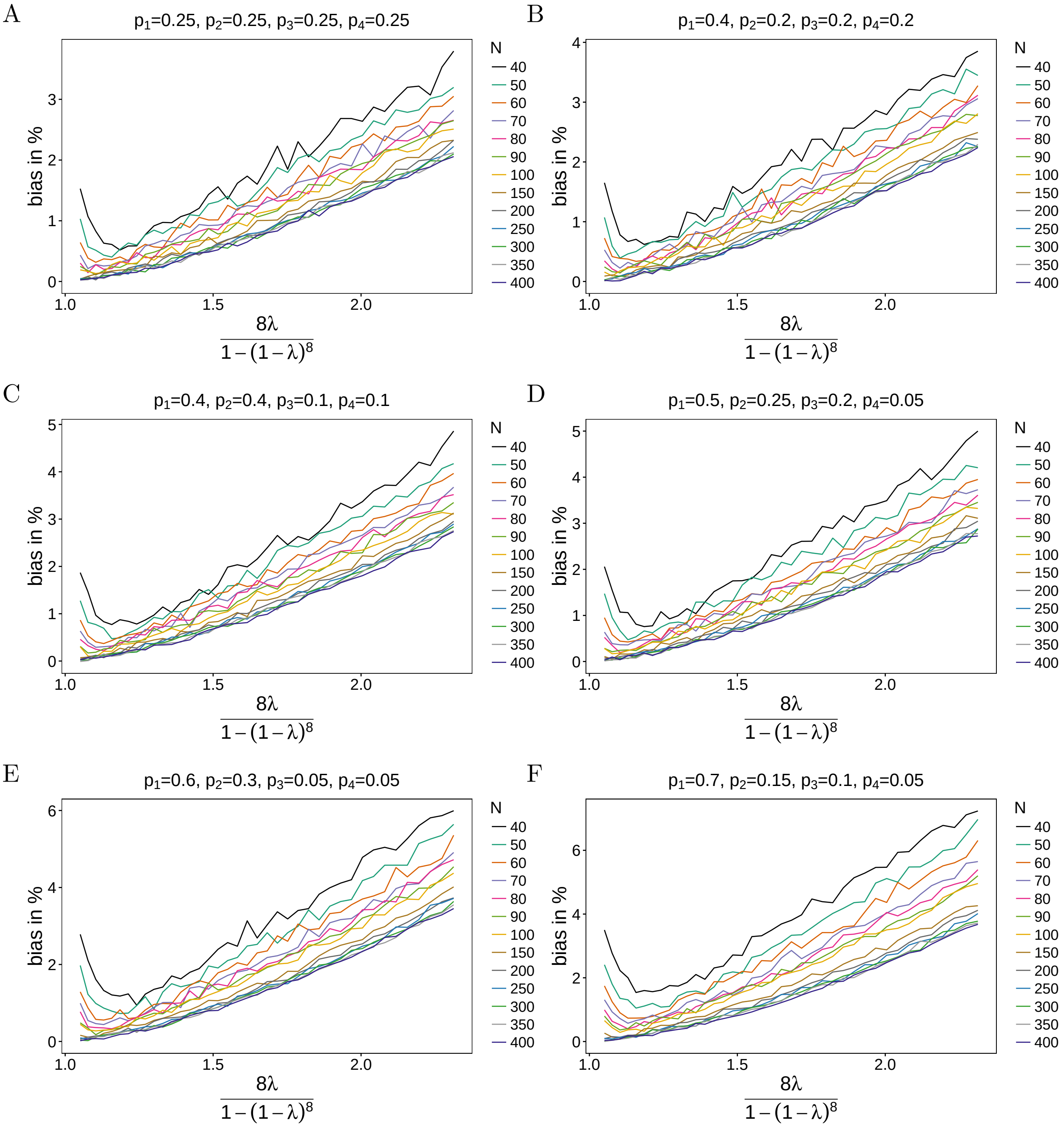

Figure 45: See Fig 42.

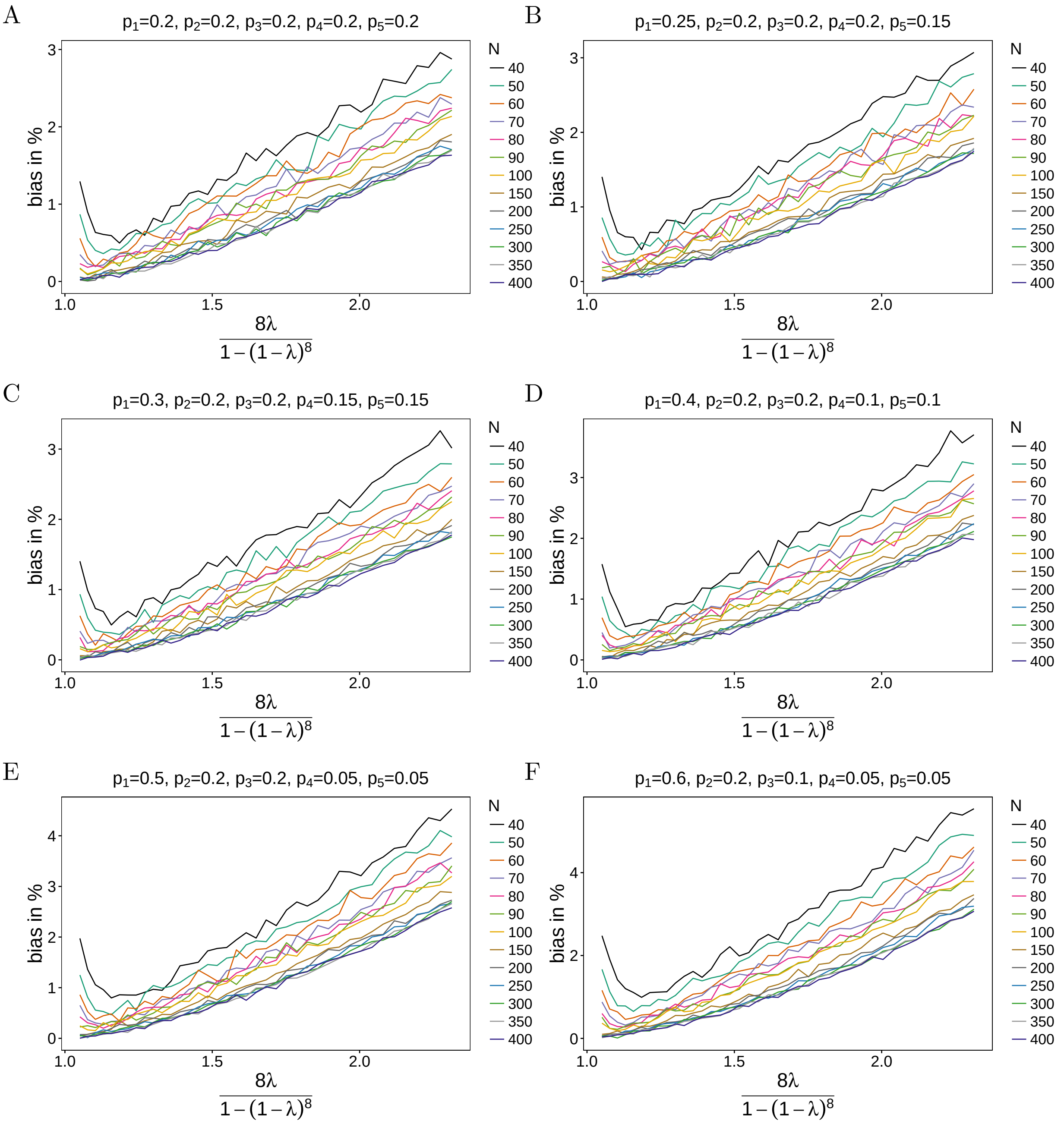

Figure 46: See Fig 42.

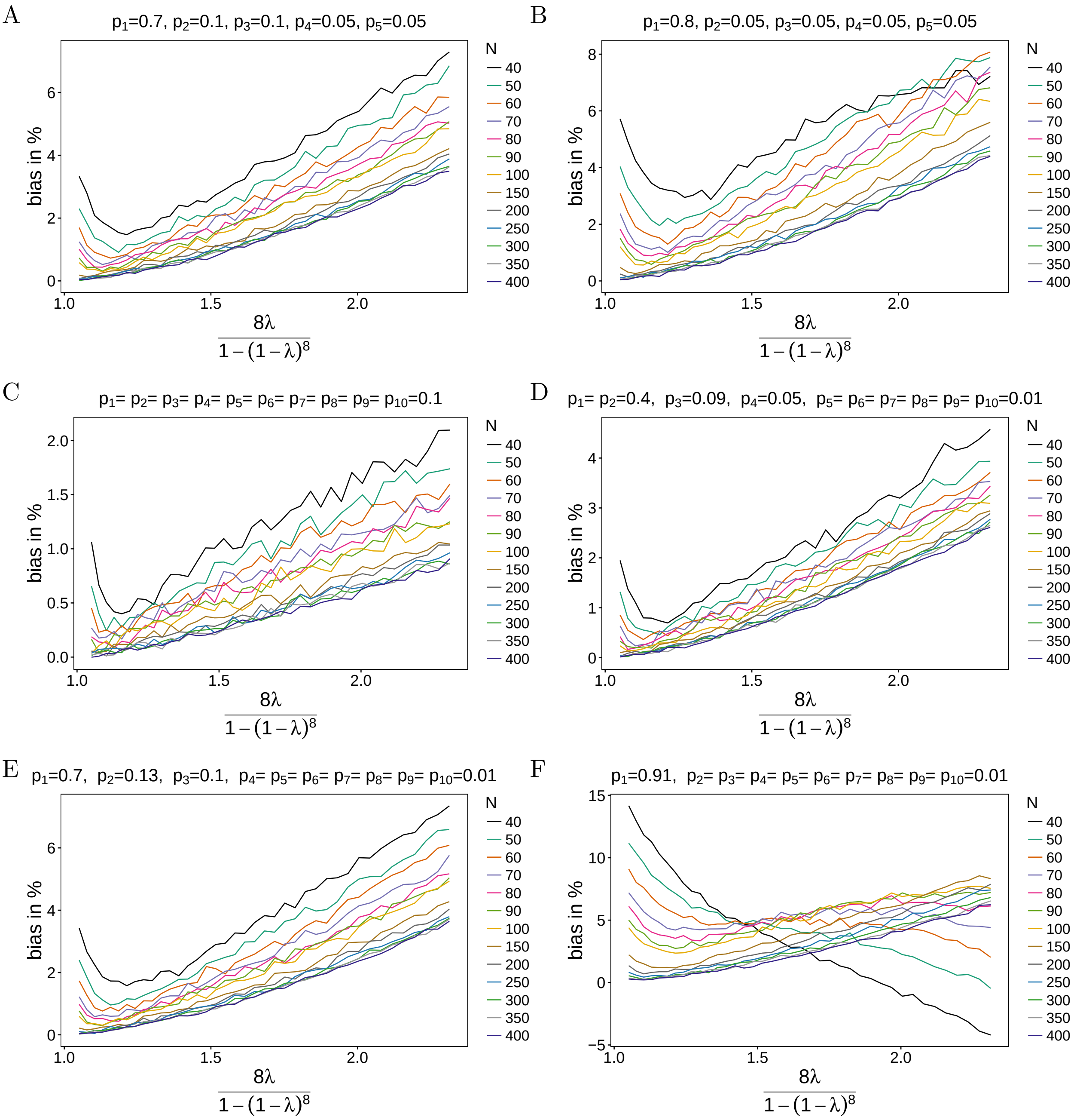

Figure 47: See Fig 42.

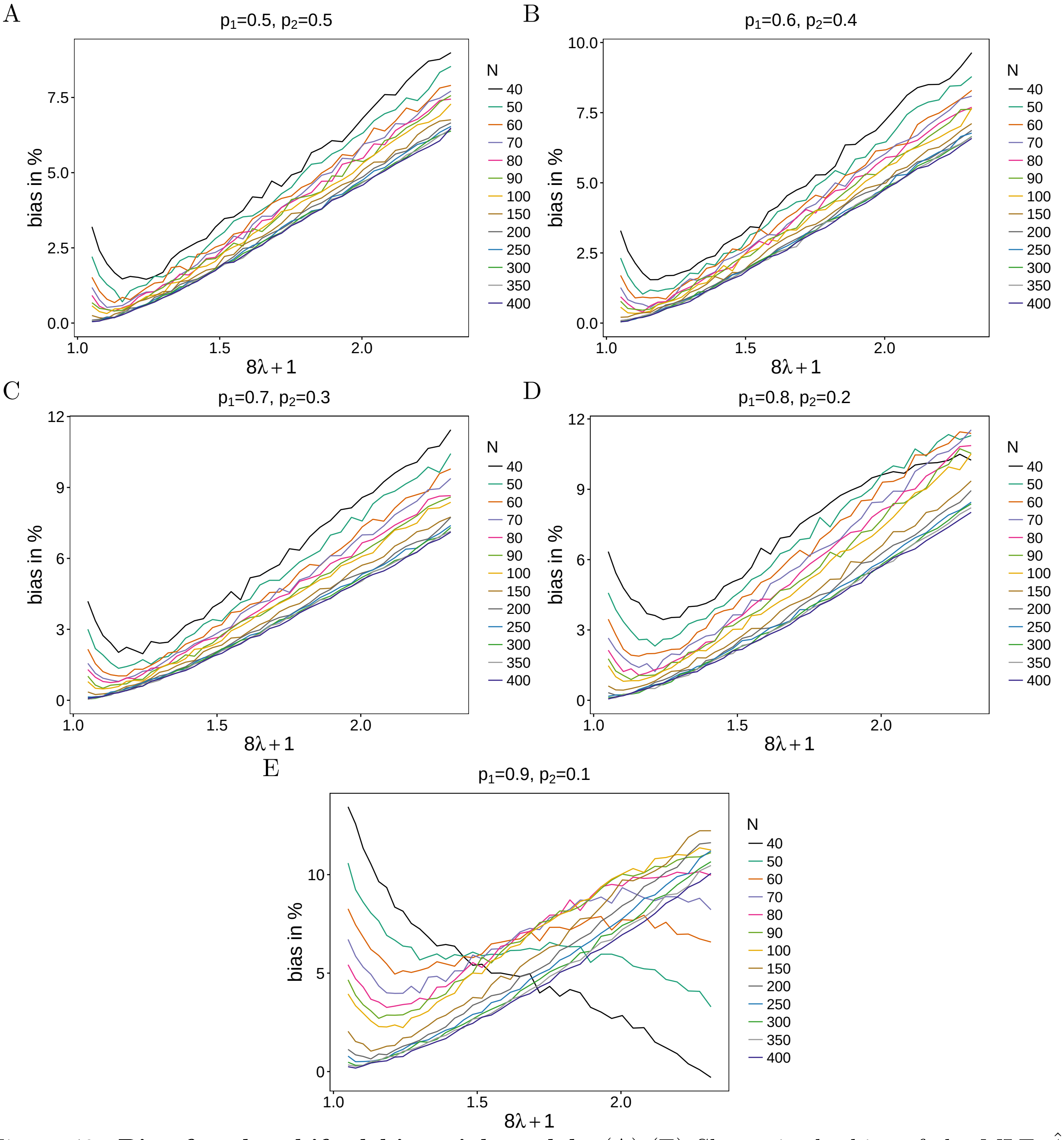

Figure 48: **Bias for the shifted binomial model.** (A)-(E) Shown is the bias of the MLE $\hat{\psi}$ in percent of the true parameter $\psi = 8\lambda + 1$ as a function of the true parameter ψ based on simulated data created by the shiftedl binomial model. For each parameter combination $K = 10\,000$ data sets were simulated. Each panel assumes different lineage frequency distributions $\boldsymbol{p}$ shown at the top of each panel. Colored lines correspond to different sample sizes N.

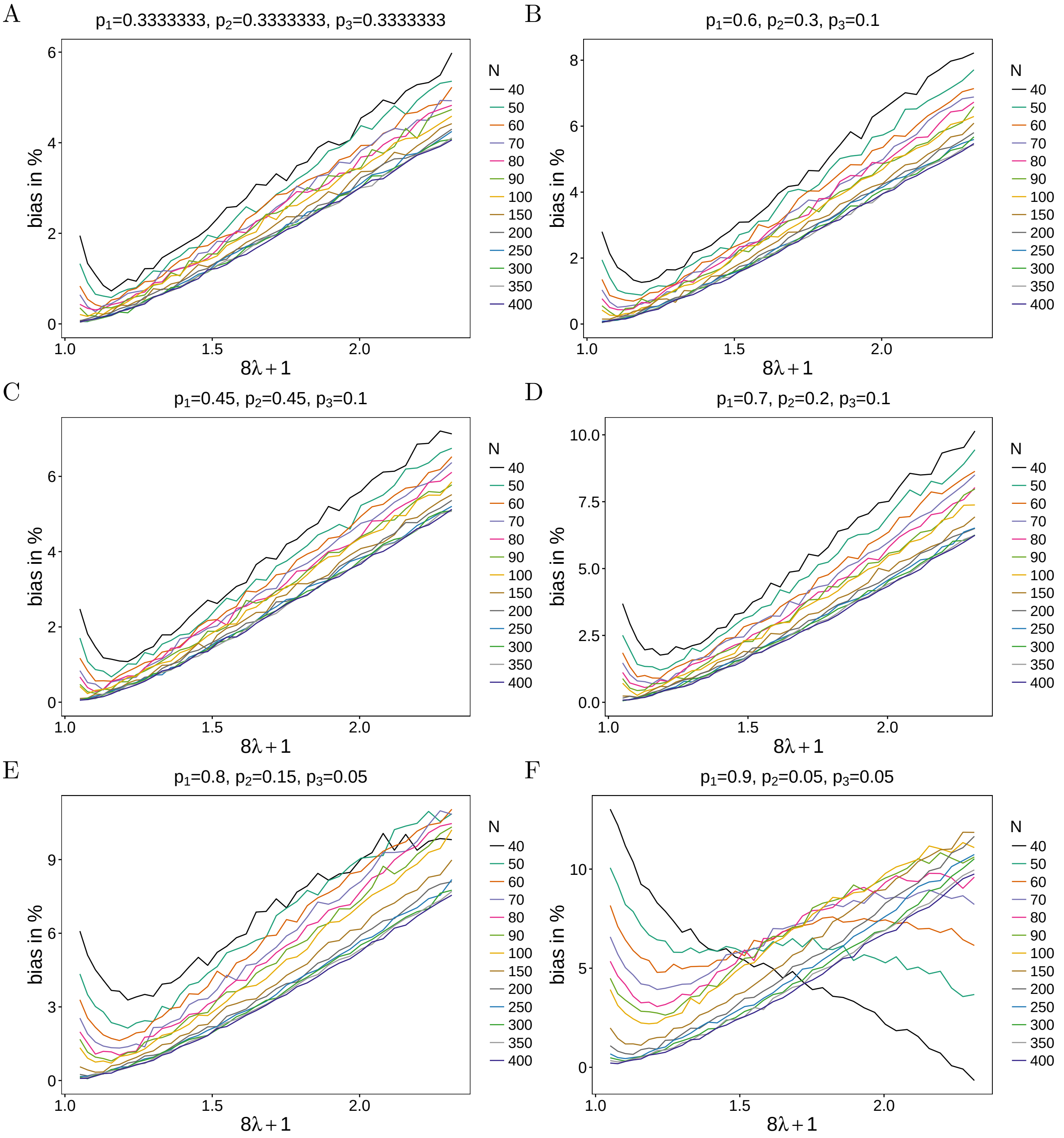

Figure 49: See Fig 48.

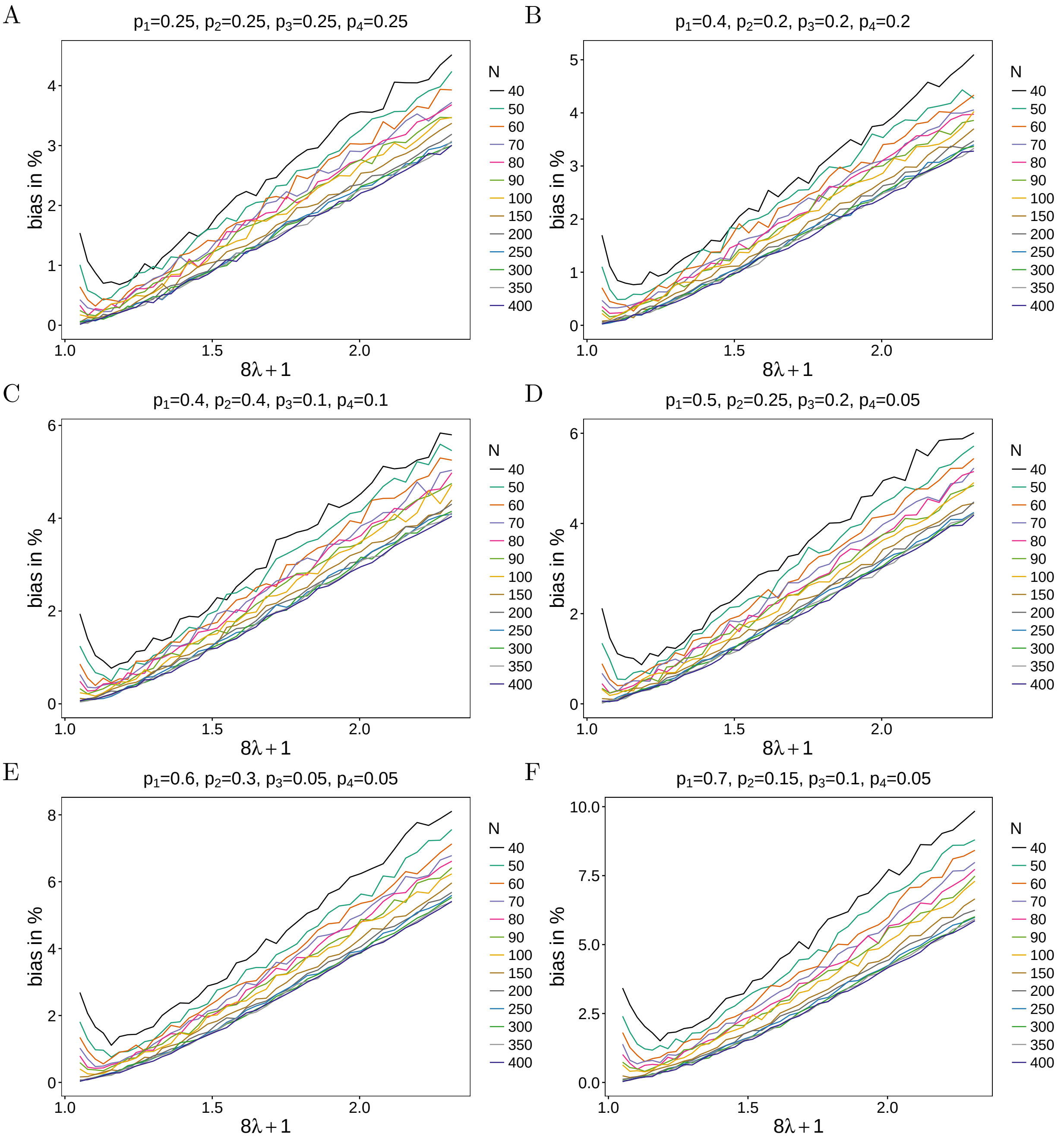

Figure 50: See Fig 48.

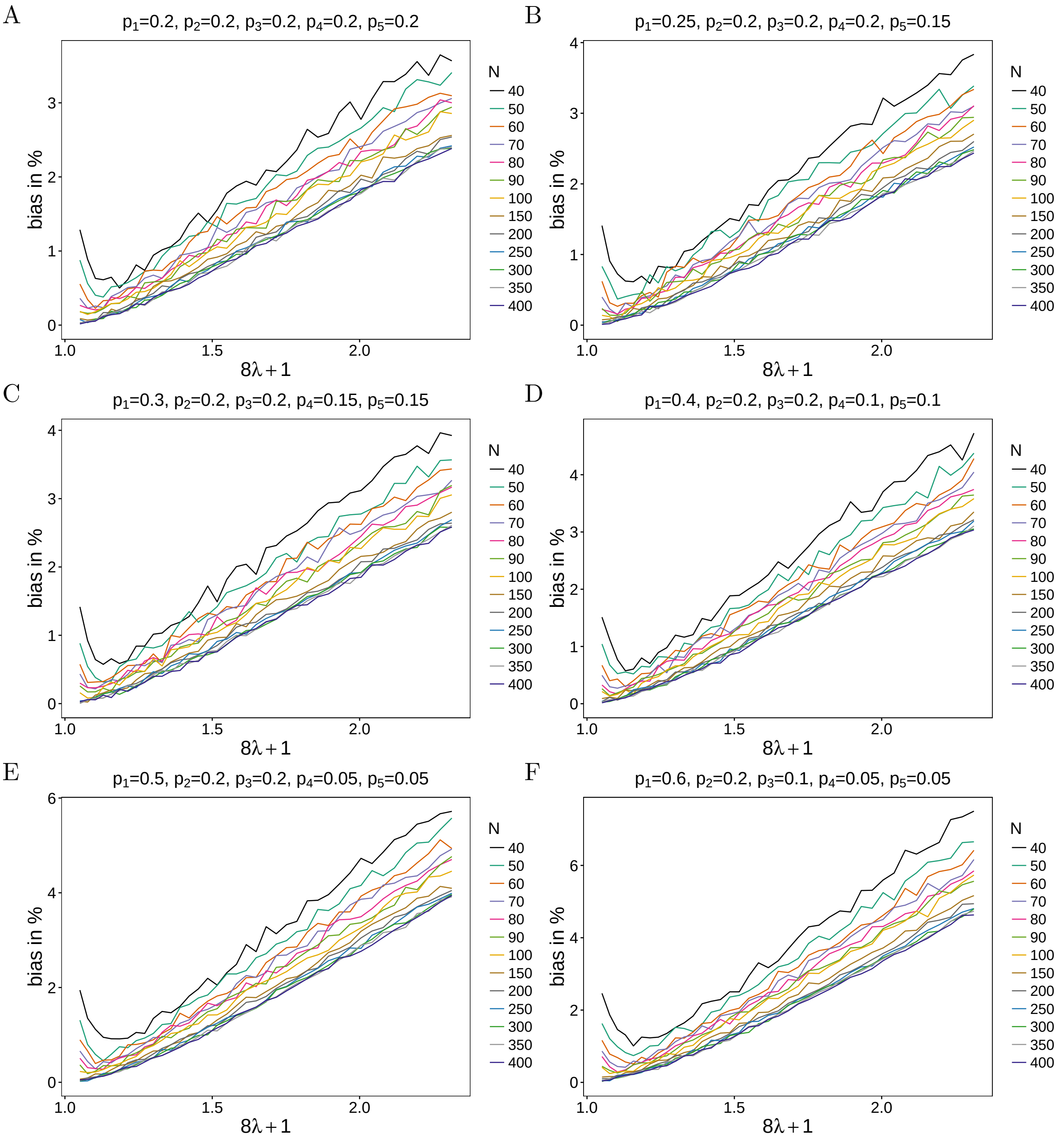

Figure 51: See Fig 48.

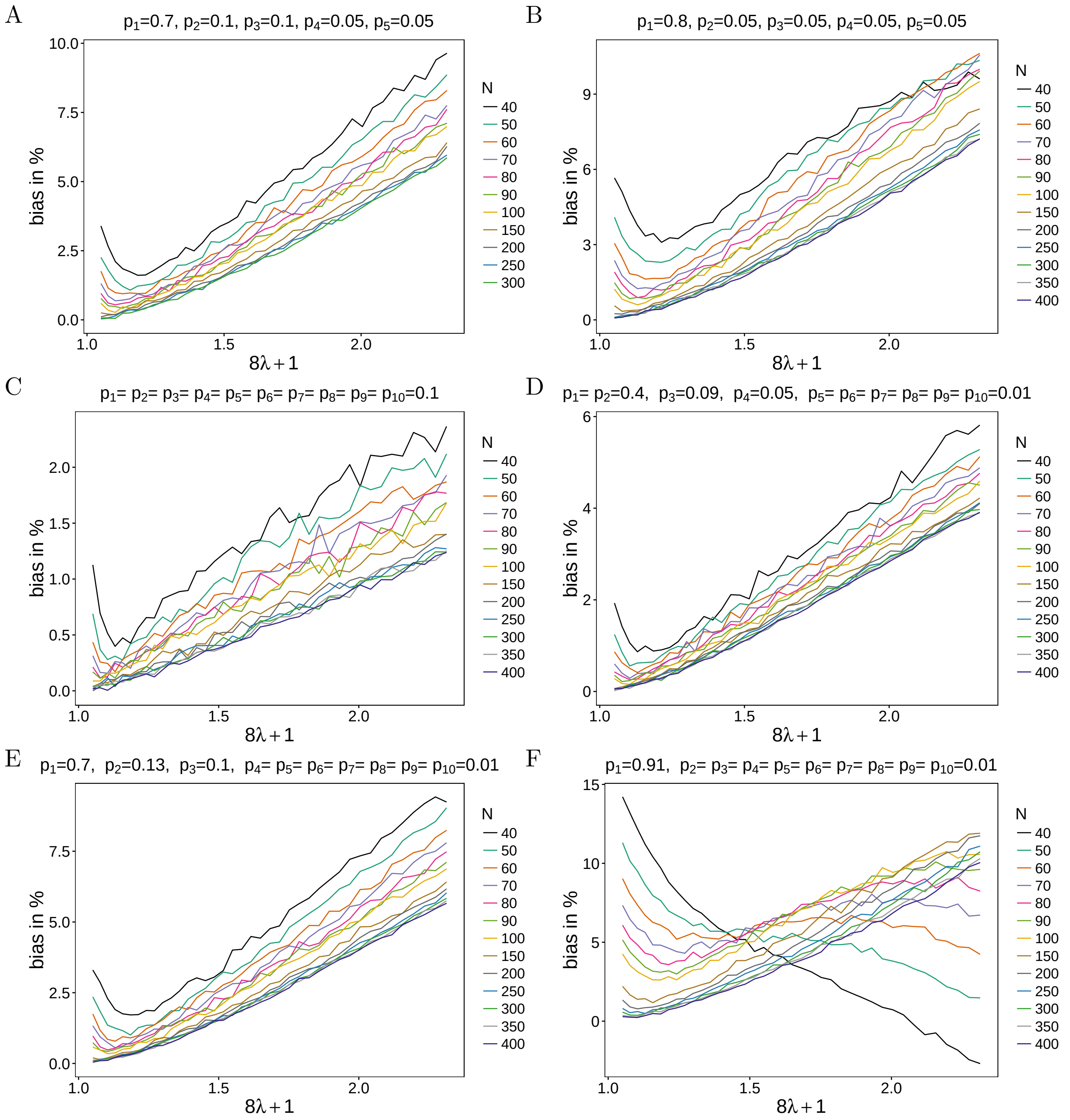

Figure 52: See Fig 48.

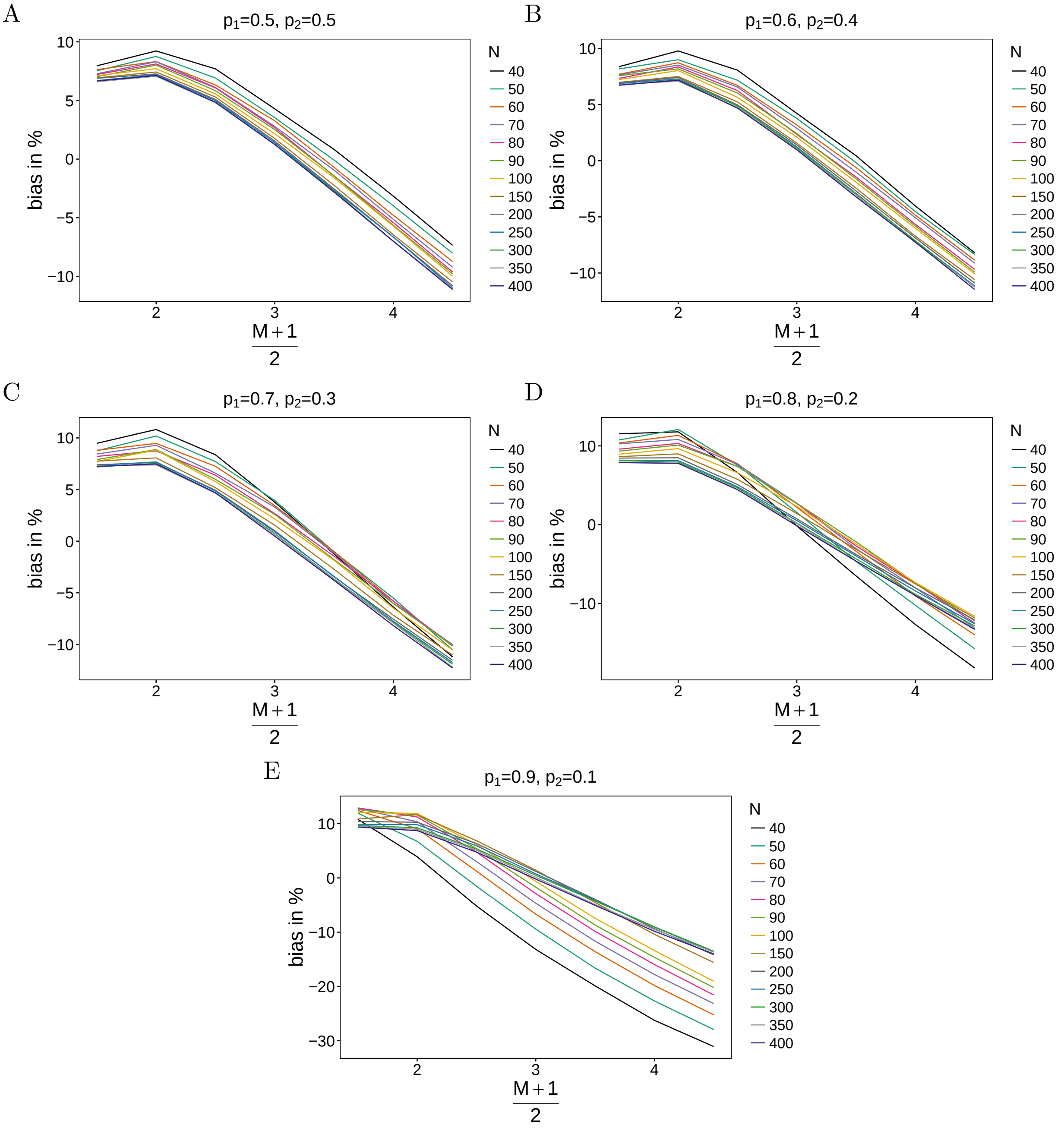

Figure 53: **Bias for the uniform model.** (A)-(E) Shown is the bias of the MLE $\hat{\psi}$ in percent of the true parameter $\psi = \frac{8\lambda}{1-(1-\lambda)^8}$ as a function of the true parameter ψ based on simulated data created by the uniform model. For each parameter combination $K = 10\,000$ data sets were simulated. Each panel assumes different lineage frequency distributions $\boldsymbol{p}$ shown at the top of each panel. Colored lines correspond to different sample sizes N.

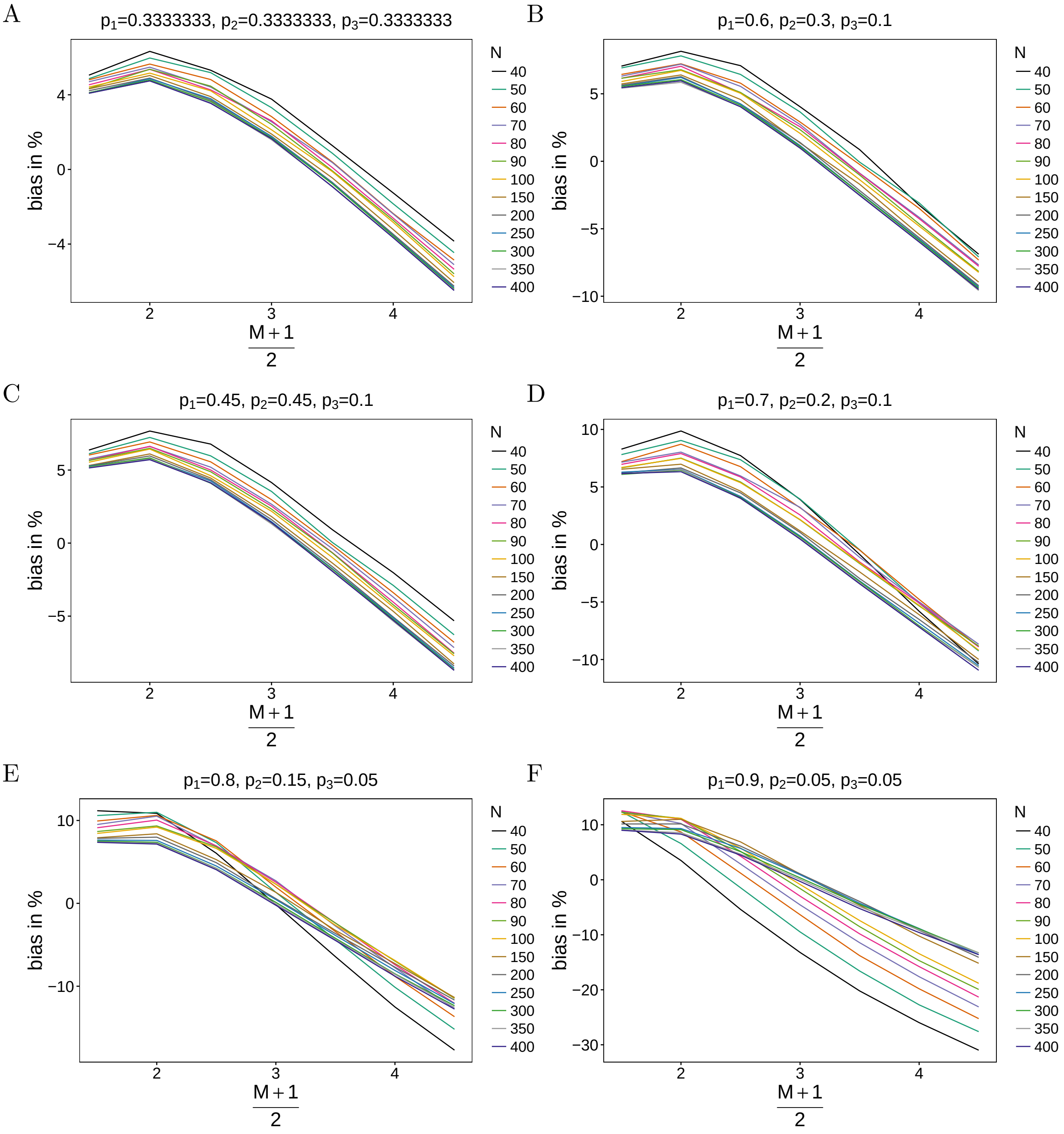

Figure 54: See Fig 53.

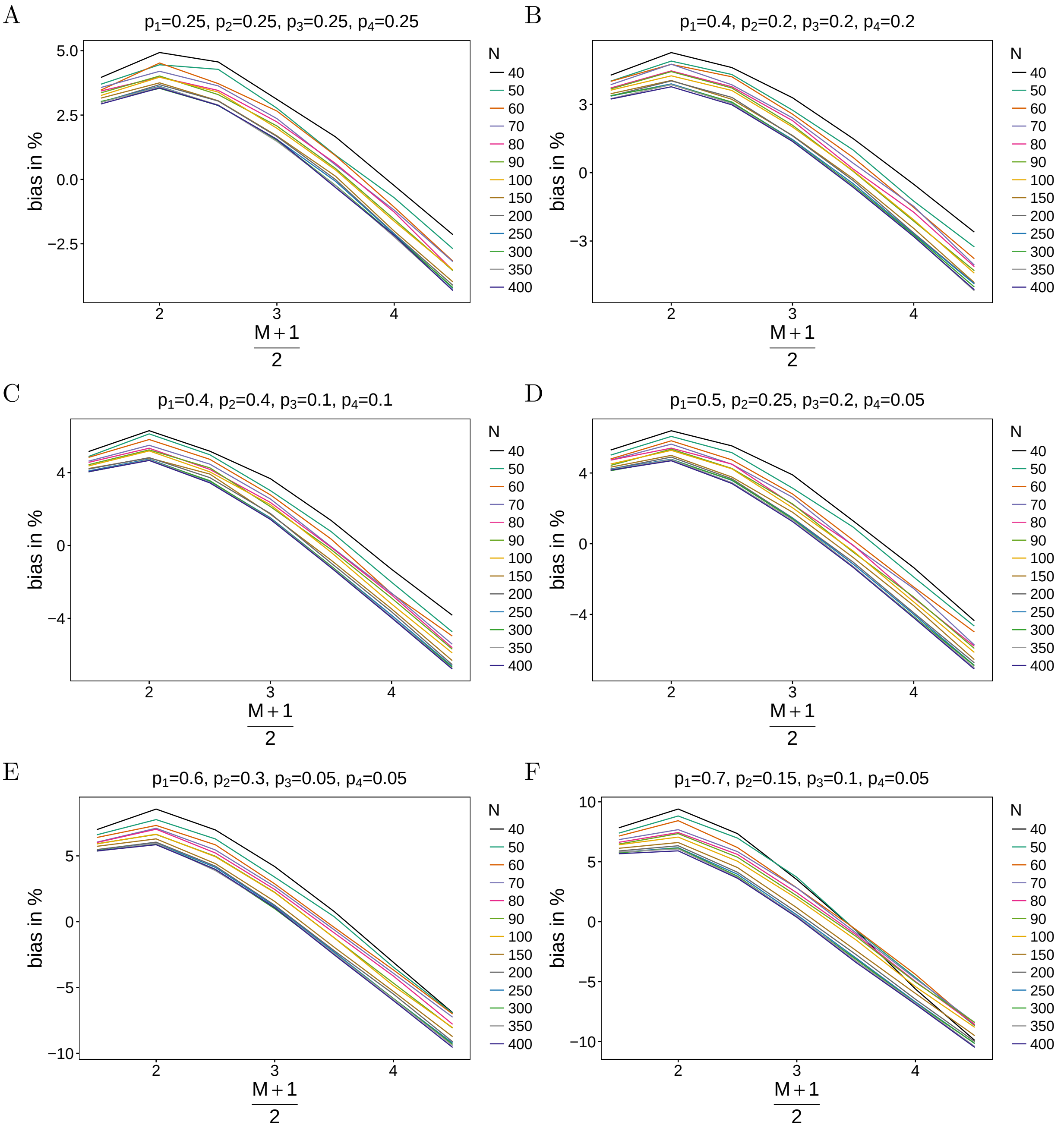

Figure 55: See Fig 53.

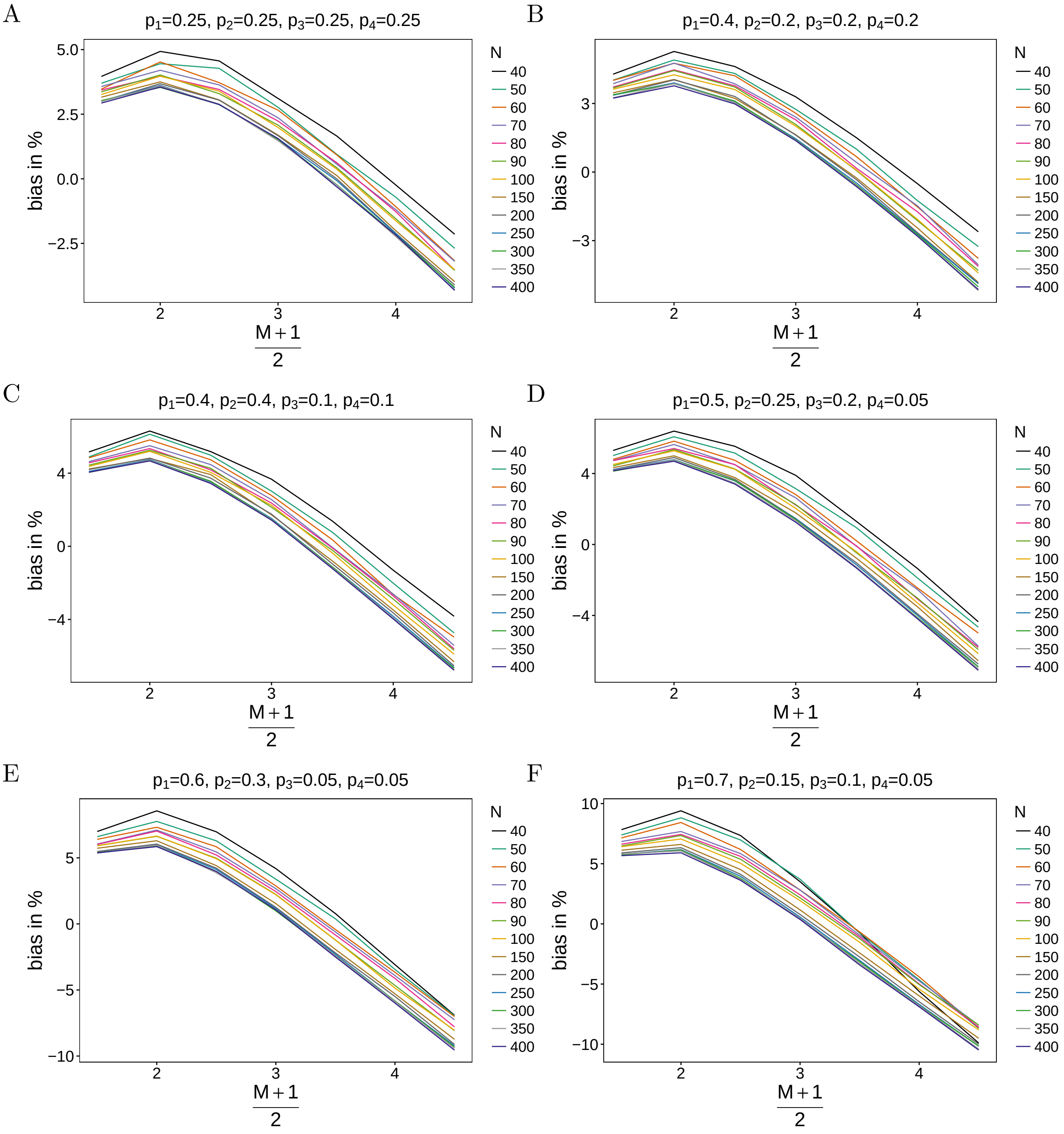

Figure 56: See Fig 53.

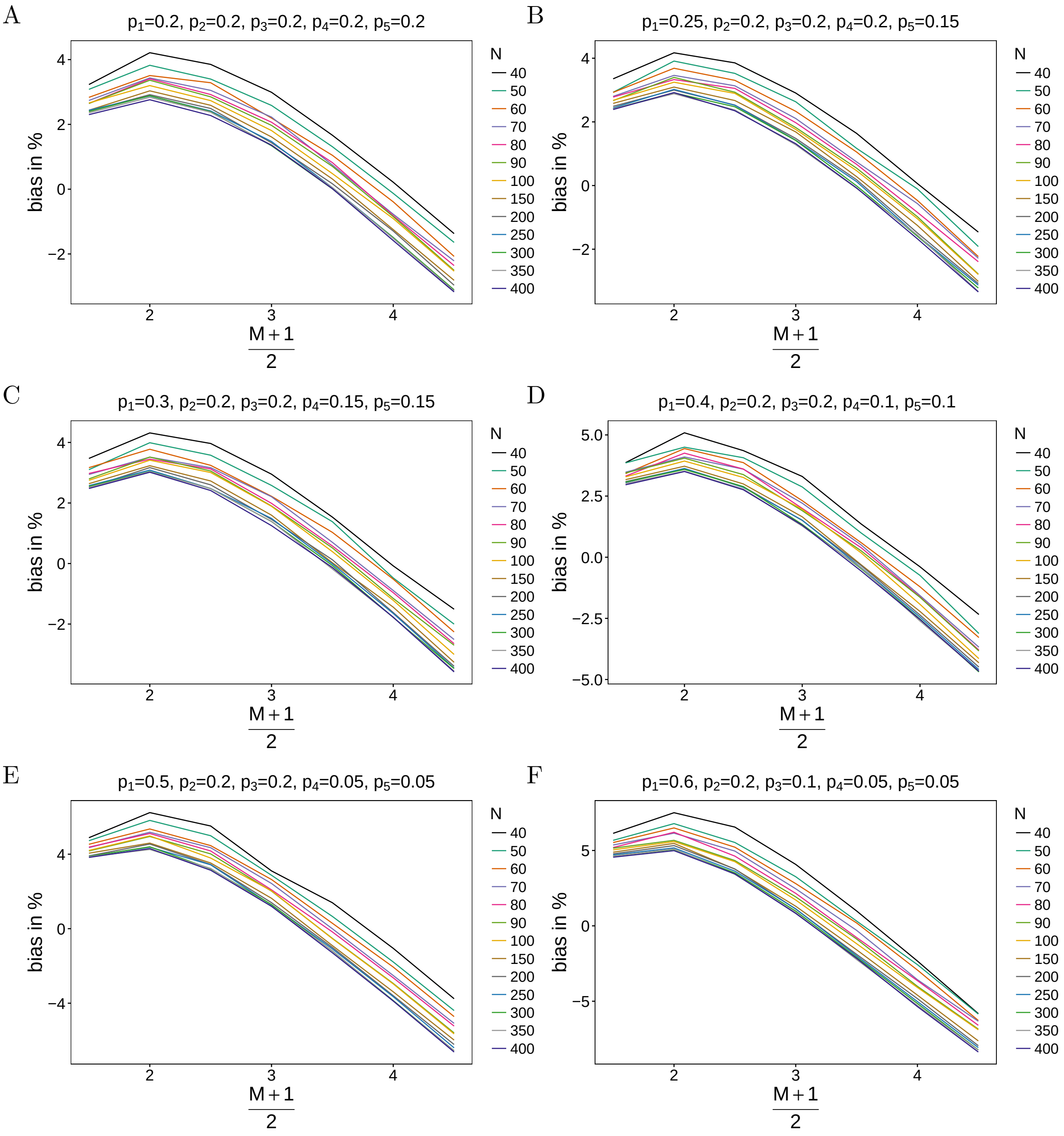

Figure 57: See Fig 53.

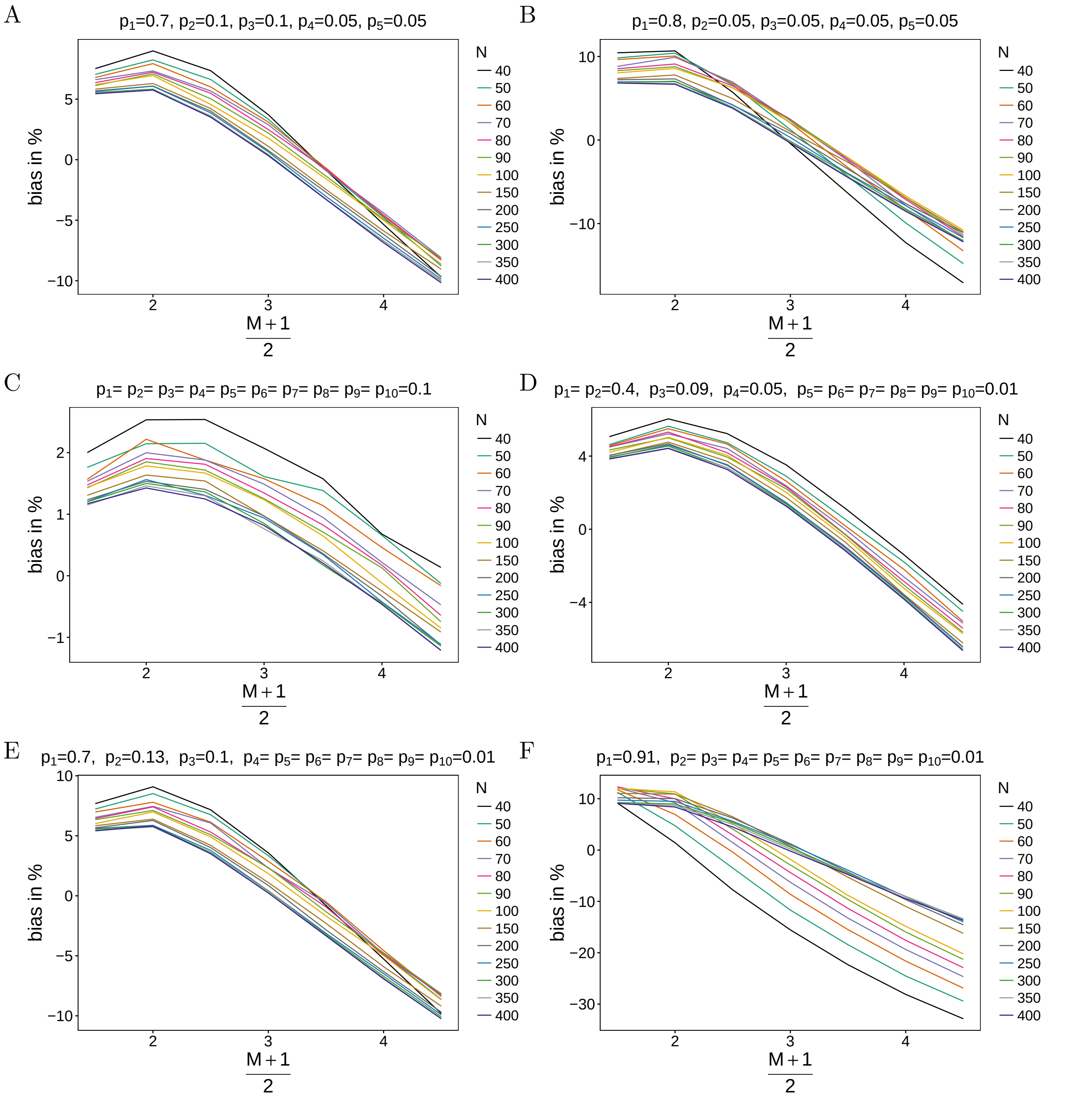

Figure 58: See Fig 53.
